# An effective method for the simultaneous extraction of 173 contaminants of emerging concern in freshwater invasive species and its application

**DOI:** 10.1007/s00216-023-04974-3

**Published:** 2023-09-30

**Authors:** Diana P. Manjarrés-López, Dyana Vitale, Sandra Callejas-Martos, Martí Usuriaga, Yolanda Picó, Sandra Pérez, Nicola Montemurro

**Affiliations:** 1 Environmental and Water Chemistry for Human Health (ONHEALTH) Group, Institute of Environmental Assessment and Water Research (IDAEA-CSIC), c/Jordi Girona 18-26, 08034 Barcelona, Spain; 2 Desertification Research Centre (CIDE) (CSIC-UV-GV), University of Valencia, Road CV-315 Km 10.7, Moncada, 46113 Valencia, Spain

**Keywords:** Aquatic contamination, Perfluoroalkyl substances, Pharmaceuticals, Aquatic fauna, LC-MS/MS, Ultrasound-assisted solvent extraction

## Abstract

A robust and efficient extraction method was developed to detect a broad range of pollutants of emerging interest in three freshwater invasive species: American red crab (*Prokambarus clarkii*), Asian clam (*Corbicula fluminea*), and pumpkinseed fish (*Lepomis gibbosus*). One native species, “petxinot” clam (*Anodonta cygnea*), was also evaluated. Invasive species are often more resistant to contamination and could be used in biomonitoring studies to assess the effect of contaminants of emerging concern on aquatic ecosystems while preserving potentially threatened native species. So far, most extraction methods developed for this purpose have focused on analyzing fish and generally focus on a limited number of compounds, especially analyzing compounds from the same family. In this sense, we set out to optimize a method that would allow the simultaneous extraction of 87 PhACs, 11 flame retardants, 21 per- and poly-fluoroalkyl substances, and 54 pesticides. The optimized method is based on ultrasound-assisted solvent extraction. Two tests were performed during method development, one to choose the extraction solvent with the best recovery efficiencies and one to select the best clean-up. The analysis was performed by high-performance liquid chromatography coupled to high-resolution mass spectrometry. The method obtained recoveries between 40 and 120% and relative standard deviations of less than 25% for 85% of the analytes in the four validated matrices. Limits of quantification between 0.01 ng g^−1^ and 22 ng g^−1^ were obtained. Application of the method on real samples from the Albufera Natural Park of Valencia (Spain) confirmed the presence of contaminants of emerging concern in all samples, such as acetaminophen, hydrochlorothiazide, tramadol, PFOS, carbendazim, and fenthion. PFAS were the group of compounds with the highest mean concentrations. *C. fluminea* was the species with the highest detection frequency, and *P. clarkii* had the highest average concentrations, so its use is prioritized for biomonitoring studies.

**Graphical abstract:**

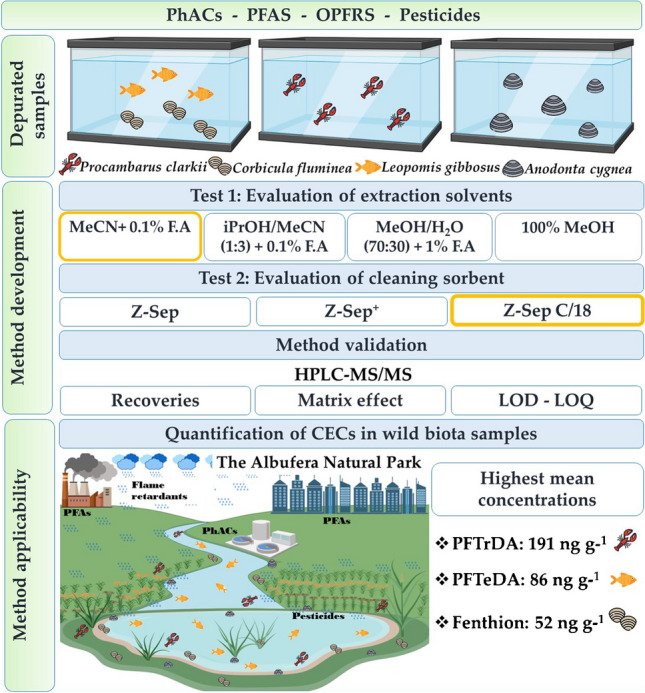

**Supplementary Information:**

The online version contains supplementary material available at 10.1007/s00216-023-04974-3.

## Introduction

The detection of contaminants of emerging concern (CECs) such as pharmaceutically active compounds (PhACs), pesticides, perfluoroalkyl substances (PFAS), and organophosphorus flame retardants (OPFR) in freshwater samples [1–13] has been gaining importance in recent years because their presence represents a risk to the environment [14]. These contaminants are diffusely released into the environment through runoff water or outlet water from wastewater treatment plants (WWTPs). Once in the water cycle, they can be taken up by aquatic organisms and may bioaccumulate in tissues. Their analysis in aquatic biota could provide more insight into pollution relationships than measuring discrete levels in water or sediment samples [3, 15].

The process of globalization, industrialization, deliberate introduction of exotic species for food production, and climate change facilitate the spread of species beyond their natural habitats where they encounter favorable conditions, e.g., due to the absence of predators. When invasive species easily adapt to a new environment, they can become dominant and displace the ecological niche of native fauna. This is the case of the American red crab (*Procambarus clarkii*), the Asian clam (*Corbicula fluminea*), and the pumpkinseed fish (*Lepomis gibbosus*), which have become invasive species in Mediterranean wetlands, including the Albufera Natural Park (Valencia, Spain). *P. clarkii* is a crayfish native to North America, which was introduced for commercial purposes in the 1970s in the Guadalquivir marshes (Southwest Spain). The economic benefits of its introduction encouraged its exploitation in areas with similar habitats. The climatic conditions of the southern Iberian Peninsula and the absence of natural predators allowed this species’ vast and rapid expansion [16, 17]. *C. fluminea* is a clam native to East Asia that has become a dominant invasive species in the Iberian Peninsula, mainly in tidally influenced lotic habitats [18]. *L. gibbosus* is native to North America and was introduced in Europe in 1880 along with other sport fishing species and as an ornamental species. The oldest documented presence in the Iberian Peninsula was between 1910 and 1913, identified in Lake Banyoles, northern Spain [19].

Invasive species could be used as integrative bioindicators to assess exposure to chemical in freshwater ecosystems. Due to their high adaptability, invasive species tend to have greater tolerance to environmental stresses. *P. clarkii*, for example, is more resilient and a better competitor than the native *Austropotamobius pallipes*, a highly endangered European freshwater crayfish. It can withstand lower oxygen levels, higher temperatures (30 °C), and high water pollution [16]. The use of invasive species in biomonitoring studies would reduce pressure on native endangered species. Invasive species populations are usually larger and their use for environmental analysis would not generate alterations in ecosystems. However, most of the active biomonitoring of CECs in freshwater ecosystems has been performed on fish and macroinvertebrates. Furthermore, most of the studies focus on a small number of compounds [20]. Due to the low expected levels of CECs and the complexity of the matrix, sensitive and robust analytical methods are essential. Ultrasound-assisted solvent extraction (UAE) is one of the most common extraction methods for CECs in aquatic biota [9, 12, 21–32]. Extraction of complex biological matrices typically features a clean-up step to ensure removal of co-extracted matrix components that otherwise may interfere in the quantification. The clean-up methods widely used in this type of analysis are solid-phase extraction (SPE) and dispersive solid-phase extraction (d-SPE) [20].

In this regard, we aimed to develop and validate a multiresidue method that allows for simultaneous extraction of four groups of CECs (87 PhACs, 54 pesticides, 21 PFAS, and 11 OPFRS) and their subsequent detection and quantification in three invasive species from the Albufera Natural Park (red swamp crayfish, Asian clam, and pumpkinseed fish) and the native species “petxinot” (*Anodonta cygnea*). The conditions of (a) sample extraction and (b) clean-up of the extracts were optimized in separate experiments. For the former, different extraction solvents were tested to maximize recovery efficiencies, while for the latter, different d-SPE materials were compared with respect to their effectiveness in reducing matrix effects (ME) in the LC-MS. The extraction solvents evaluated were **A** acetonitrile (MeCN) + 0.1% formic acid (F.A), **B** isopropanol/acetonitrile (iPrOH/MeCN) (1:3) + 0.1% F.A, **C** water/methanol (H_2_O/MeOH) (7:3) + 1% F.A, and **D** 100% MeOH. The d-SPE sorbents evaluated were as follows: **A** Z-Sep, **B** Z-sep^+^, and **C** Z-Sep/C_18_. Once the extraction method was validated, it was used to analyze real samples collected in the Albufera Natural Park.

## Materials and methods

### Chemicals and standards

The chemical standards (Table S1) as well as the isotopically labeled standards (IS) (Table S2) were purchased from Sigma-Aldrich (St. Louis, MO, USA), Sigma-Aldrich (Steinheim, Germany), Toronto Chemicals Research (Toronto, Ontario, Canada), CDN Isotopes (Pointe-Claire, Quebec, Canada), Santa Cruz Biotechnology (Dallas, TX), Wellington (Ontario, Canada), and Cymit Quimica S.L. (Barcelona, Spain). LC-MS grade MeCN (≥ 99.9%), iPrOH (≥ 99.9%), HPLC grade water, and MeOH (≥ 99.9%) used as extraction solvents were obtained from J.T.Baker (Fisher Scientific SL, Madrid, Spain). F.A (≥ 96%, ACS reagent) was obtained from Sigma-Aldrich. The d-SPE sorbents (Z-Sep/C_18_, Z-Sep^+^, and Z-Sep) were obtained from Supel TM QuE, Supelco/Sigma-Aldrich, St. Louis, MO, USA. MeCN and water (Optima™ LCMS Grade) used for the preparation of the mobile phases were obtained from Fisher Chemical. Ammonium acetate and ammonium fluoride were obtained from Sigma-Aldrich and Fisher Chemical (Fisher Scientific SL, Madrid, Spain). The standard mixtures used for the calibration curve were prepared in serial dilutions from a stock solution of 2 µg mL^−1^. A matrix-matched calibration curve was prepared by spiking the extracts to obtain ten points, from 0.1 to 500 ng g^−1^ (wet weight, w.w).

### Sample collection and pretreatment

The species used to develop, optimize, validate, and evaluate the applicability of the extraction method were collected at the Albufera Natural Park. This ecosystem is a coastal lagoon with marine water influences, as well as wastewater impacts, but at present, it is mainly a freshwater system. It is located 10 km south of Valencia (Spain) and covers an area of 210 km^2^. It constitutes a natural refuge for diverse biological species, and activities such as agriculture (mainly rice fields), fishing, hunting, urban, and industrial construction interact there [33].

To reduce costs and analytical time, a native species (*A. cygnea*) and an invasive species (*P. clarkii*) were selected for the development of the extraction method. The specimens were obtained from a fish farm located in the Natural Park. Of the analytes studied, PFAS have the longest half-life. This is why the samples were subjected to a 14-day cleanup process according to the depuration rates reported for PFAS in oysters [34] and aquatic invertebrates [35]. The depuration was carried out in 26-L tanks with a closed circuit of clean water and a filtration module to eliminate contaminants and provide the cleanest possible matrix. Once the analytical method was developed, it was validated for four species.

To demonstrate the method applicability, real samples of the three invasive species were collected: *C. fluminea* was collected in the irrigation channels of Rec de Tonyiner (351 specimens divided into groups of 50 to obtain seven pooles samples plus a sample of a single larger clam), *P. clarkii* was captured in the areas surrounding the rice paddies of “Sollana” and “El Palmar” (32 specimens divided according to their weight to obtain 17 pooled samples), and *L. gibbosus* from the “Sueca-El Palmar” canals and the fish farm settling ponds (13 specimens divided into seven samples according to their weight). In contrast to high population of invasive species, the native species was not sample to protect the existing population.

For the sample pretreatment, the soft tissue of each species was obtained, removing the shell in the case of clams and the exoskeleton in the case of crabs, while the whole organism was used in the case of fish. The tissues were homogenized using a knife mill with a stainless steel grinding chamber (Grindomix GM 200, Retsch GmbH, Haan, Germany) and stored at −40 °C until extraction.

### Method development

The extraction protocol implemented was based on a method previously reported by [26] for fish tissues. In this case, the extraction efficiency of four solvents was evaluated: **A** MeCN + 0.1% F.A facilitates the recovery especially of more polar compounds; **B** iPrOH/MeCN (1/3) + 0.1% F.A tends to improve the recovery of less polar compounds; **C** H_2_O/MeOH (7/3) +1% F.A has shown satisfactory recoveries under acidic conditions; and **D** 100% MeOH tends to obtain good recoveries, especially of more lipophilic compounds. We evaluated solvents under acidic conditions because most compounds ionize in positive mode. Only PFAS and 18 PhACs ionize in negative mode (Table S1). However, according to [36], PFAS had satisfactory recoveries with acidified solvents. Nevertheless, to favor the ionization of negative compounds, ammonium fluoride was used in the mobile phases during chromatography, as shown in Table 1.

**Table 1:** Chromatographic and analytical conditions by compounds group

Compounds	Conditions	LC-MS/MS	Elution gradient				Reference
			Time (min)	Aqueous phase (%)	Organic phase (%)	Flow (mL min^−1^)	
PhACs	*Column:* EVO C18 KINETEX (100 × 2.1 mm; 2.6 µm. Phenomenex). *Mobile phases:* H_2_O 5mM ammonium acetate + 0.1% F.A and MeCN for ESI (+) H_2_O 2mM NH_4_F and MeCN for ESI (-)	Waters Acquity UPLC system (Waters. Milford. MA). Orbitrap Q-Exactive™ mass spectrometer (Thermo Fischer Scientific, San Jose, CA,USA)	0.0	95	5	0.2	[26]
			0.3	95	5	0.2	
			10	70	30	0.2	
			13.3	35	65	0.2	
			15.5	0	100	0.2	
			17.3	0	100	0.2	
			17.7	95	5	0.2	
			19.0	95	5	0.2	
OPFRs	*Column:* KINETEX XB-C18 (50.0 × 2.1 mm. 1.7 µm. Phenomenex. Torrance. USA) *Trap Column:* ZORBAX Eclipse Plus C18 (4.6 × 30 mm; 3.5 µm. Agilent. Santa Clara. USA). *Mobile phases:* H_2_O + 0.1% F.A and MeOH +0.1% F.A	Thermo Scientific™ Vanquish™ UHPLC system. Thermo Scientific™ Orbitrap Exploris 120™ mass spectrometry	0.0	70	30	0.4	[37]
			0.5	79	30	0.4	
			12	2	95	0.4	
			18	2	98	0.4	
			18.1	70	30	0.4	
			28	70	30	0.4	
Pesticides	*Column:* Luna C18 (15.0 cm × 0.21 cm. 3 μm. Phenomenex. Torrance. USA). *Mobile phases:* H_2_O 5mM ammonium formate + 0.1% F.A MeOH 5mM ammonium formate + 0.1% F.A		0.0	50	50	0.4	[38]
			10.0	17	83	0.4	
			12.0	17	83	0.4	
			12.5	2	98	0.4	
			15.5	2	98	0.4	
			15.6	50	50	0.4	
			25.6	50	50	0.4	
PFAS	*Column:* Kinetex^®^ EVO C18 KINETEX (100 × 2.1 mm; 2.6 µm. Phenomenex. Torrance. USA). *Mobile phases:* H_2_O + 4 mM NH_4_F and MeOH + 4 mM NH_4_F for ESI (-)	UHPLC Agilent 1260 HPLC (degasser, quaternary pump, and column oven) Eksigent ULC 100 HTC-xt autosampler. Sciex TRIPLE QUAD 6500+ mass (Sciex, Concord, Ontario, Canada)	0.0	95	5	0.3	[39]
			0.5	70	30	0.3	
			12	5	95	0.3	
			18	5	95	0.3	
			30	95	5	0.3	

In brief, for the extraction 500 mg of homogenized soft tissue was weighed into a 2-mL Eppendorf tube. Then, 50 µL of the working solution containing the analytes (200 ng mL^−1^) was added to the sample to obtain a concentration of 10 ng mL^−1^ in the final extract, and allowed to stand under the fume hood for 30 min. Samples were homogenized again by placing two stainless steel beads (5 mm Ø) into the Eppendorf and operating a TissueLyzer LT sample disruptor (Quiagen, Germany) at 50 Hz for 90 s, and allowed to stand for 15 min. One milliliter of the extraction solvent to be evaluated was added (A, B, C, or D); the samples were vortexed for 90 s, sonicated for 10 min (Fisherbrand® FB15064, Waltham, MA, USA), and centrifuged for 12 min at −9 °C and 20854 G-force (Eppendorf AG 5810 R Hamburg, Germany). Finally, 500 µL of the supernatant was transferred to an HPLC vial, evaporated to dryness (TurboVap® LV, Biotage. Uppsala, Sweden), and reconstituted in 500 μL 5mM ammonium acetate in water/MeCN (9/1).

After selecting the extraction solvent with the most efficient recoveries and the lowest relative standard deviation (RSD), a second series of experiments were performed in which different d-SPE sorbents were evaluated to clean the extracts and remove interfering co-eluents from the matrix. The Z-Sep sorbent has previously demonstrated its utility for removing matrix components from samples containing a large number of both polar and non-polar analytes [12, 36, 40]. The common sorbent that all Z-Sep variants have is silica gel and zirconium dioxide, which is an amphoteric oxide that presents multiple interaction pathways with matrix components. The zirconium-coated silica particles selectively remove fat and pigments from sample extracts. Three different sorbents of this type were tested. **A** The standard Z-Sep sorbent is a mixture of two sorbents, C_18_ and zirconium dioxide-coated silica. The ratio of SiO_2_+ZrO_2_:C_18_ is 2:5. Z-Sep is used for hydrophobic analytes. Lipid retention is based on Lewis acid-base interactions between hydroxyl groups and zirconium. **B** Z-Sep^+^ sorbent consists of zirconia and double-bonded C_18_ on the same silica particles and is suggested for cleaning samples with more than 15% fat content, and **C** the Z-Sep/C_18_ sorbent is a combination of Z-Sep and Discovery® DSC-18 particles. Its use is recommended for analyzing samples with less than 15% fat content. Lipid retention is based on hydrophobic interactions between triglycerides and C_18_.

For the development of the second experiment, the same protocol was followed, and a clean-up step was added to the procedure in which 750 μL of the supernatant was transferred to a vial containing the d-SPE to be evaluated (A, B, or C). It was vortexed for 1 min and centrifuged for 6 min at 20854 G-force and −2 °C. Five hundred microliters of the supernatant was transferred to an HPLC vial, evaporated, and reconstituted as described above. All samples were prepared in triplicate.

### LC-HRMS/MS analysis

Information related to the chromatographic separation, the mobile phases used for the positive and negative ionization modes, the elution gradient, the source conditions, and any detailed information regarding the methodology LC-HRMS/MS are summarized in Table 1. Data acquisition for method validation was performed with Thermo Scientific Xcalibur 4.1.31.9. Software (Thermo Fisher Scientific). Quantification was performed with a matrix-matched approach and following the internal standard method. Samples were spiked with 12.5 µL of IS (2 µg mL^−1^) used as surrogates before the extraction for quantification purposes. We used Trace Finder 5.1 software (Thermo Fisher Scientific) to quantify the analytes in the real samples. The presence of the precursor ion was confirmed by applying the following criteria: signal-to-noise ratio >10, mass accuracy of 5 ppm, equivalent retention time of 30 s compared to the reference standard, and presence of the product ion with the highest relative abundance in the MS/MS spectrum.

### Validation protocols

According to the results obtained for solvent extraction and clean-up sorbent experiments, the method with the best recoveries for most of the compounds of the four groups of analytes was selected (MeCN + 0.1% F.A + the Z-Sep/C_18_ sorbent). The method was validated at two concentration levels (10 ng g^−1^ and 100 ng g^−1^). The validation was performed for the four species: *C. fluminea*, *P. clarkii*, *L. gibbosus*, and *A. cygnea*. The performance of the extraction method was expressed in terms of recovery (%), precision (RSD), limits of detection and quantification (LOD and LOQ), and matrix effects (ME) for each specie. The accuracy and precision of the method were calculated based on the analysis of three replicates.

Recoveries (*R*, %) were determined by comparing the peak area of the analytes in the samples spiked with standards before the extraction with the peak area of analytes in the samples spiked after the extraction. The values were normalized by the area of corresponding IS, according to Eq. (1).

1 $$R \left(\%\right)=100\left(\frac{(\mathrm{ABE}-\mathrm{Blanck\, extract})/\mathrm{AIS}}{(\mathrm{AAE}-\mathrm{Blanck\, extract})/\mathrm{AIS}}\right)$$

where **ABE** is the area of the analyte before extraction, **AAE** is the area of the analyte after extraction, and **AIS** is the area internal standard.

To evaluate the ME on the analyte signal in the MS, blank extracts were spiked at the same concentration as the recovery studied (10 ng g^−1^ and 100 ng g^−1^). The relative ME was calculated by comparing the peak area of the analyte spiked after the extraction, with its peak area of a solution prepared in aliquots of 0.5 mL of injection solvent: 5mM ammonium acetate in water/MeCN (9/1) according to Eq. (2).

2 $$ME \left(\%\right)=100 x\left(\frac{\mathrm{AAE}-\mathrm{Blank\, extract}}{\mathrm{Area\, in\, solvent\, solution}}-1\right)$$

We used the matrix-matched calibration curve approach to determine the LOD and LOQ of the method by calculating the standard deviation of the calibration curve and dividing it by the slope, according to Eqs. (3) and (4).

3 $$LOD \left(ng {g}^{-1}\right)=\left(\frac{3 . {S}_{b}}{\mathrm{b}}\right)$$

4 $$LOQ \left(ng {g}^{-1}\right)=\left(\frac{10 . {S}_{b}}{\mathrm{b}}\right)$$

where ***S***_**b**_ is the standard deviation of calibration curve and **b** is the slope.

The calibration series was prepared at ten concentration levels from 0.1 to 500 ng g^−1^, and 12.5 µL of IS (1 µg mL^−1^) was added to each calibration level. Linearity was evaluated by calculating the coefficient of determination (*R*^2^). The calibration curve was constructed by weighted least squares linear regression (1/*x* as weighting factor) and was also used for quantification purposes.

### Quality assurance and quality control (QA/QC)

Samples used for development, method validation, and preparing the matrix-matched calibration curves underwent a depuration process for 14 days in fish tanks with clean water to remove contamination. The stainless steel beads used in the soft tissues’ homogenization step were washed with ethanol and acetone and placed in an ultrasonic bath for 15 min for each solvent to avoid the contamination of samples. The other materials used during the extraction process were single-use. Additional blank extracts spiked with reference standards (100 ng g^−1^) were tested, as quality control after every eight samples to evaluate method performance. Similarly, solvent blanks (MeOH) were analyzed in every four samples to verify the absence of carryover effect in the column. An interval of 30 s was an acceptable difference between the chromatographic retention times of the calibration curve and those of the samples. Moreover, a mass error of <5 ppm has been established to quantify the compounds detected in the real samples. The *R*^2^, IS, and linearity range for each compound used for quantification purposes are shown in Table S2.

## Results and discussion

### Extraction solvent optimization

**Fig. 1:**
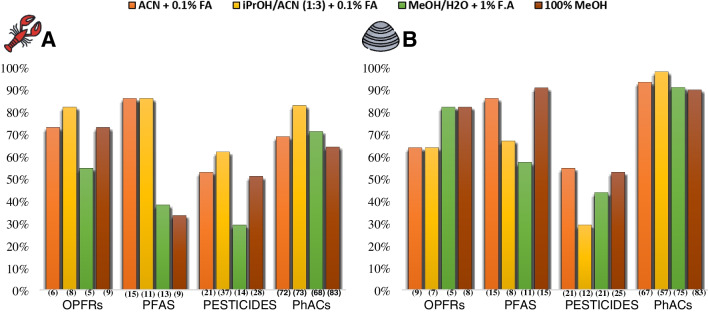
Solvent extraction test results. **A** *P. clarkii.* **B** *A. cygnea.* Bars indicate the percentage of compounds with an extraction efficiency between 40 and 120%. The percentage was calculated according to the total number of analytes in each compound group: OPRFs (11), PFAS (21), pesticides (54), and PhACs (87). (Numbers in brackets): analytes with RSD ≤ 20%.

The method development was based on previous work to analyze PhACs in complex matrices such as fish tissues/organs [12, 30]. The expected result of the present method was to extend the simultaneous extraction of CECs (87 PhACs, 54 pesticides, 21 PFAS, and 11 OPFRS) in matrices with differences in the percentage of lipid content. Considering the variability of the physicochemical properties of the analytes, we initially tested four mixtures of extraction solvents: **A** MeCN + 0.1% F.A, **B** iPrOH:MeCN (1:3) + 0.1% F.A, **C** H_2_O:MeOH (7:3) + 1% F.A, and **D** 100% MeOH. The results of the extraction efficiencies for the four groups of compounds spiked at a concentration level of 20 ng g^−1^ (w.w.) for one of the invasive species (*P. clarkii*) and the native species (*A. cygnea*) are shown in Fig. 1. The results of extraction efficiency for each compound according to each extraction solvent are reported in Table S3.

The results obtained show variability in the extraction efficiency by solvent and group of compounds. Considering an acceptable recovery between 40 and 120% as a reference, the PhACs group obtained the best extraction efficiencies, achieving the recovery of 84 analytes with solvent **B** and 80 with solvent **A** in the *A. cygnea* and 71 analytes with solvent **B** and 59 with solvent** A** in the *P. clarkii* respectively. In the case of PFAS, the same extraction efficiency was obtained with solvent** A** and solvent** B** for the *P. clarkii*, recovering 18 analytes. In contrast, for the *A. cygnea*, the highest number of PFAS was recovered with solvent** D** (19 analytes), followed by solvent **A** (18 analytes) and solvent **B** (14 analytes). For OPFRS, the best extraction efficiencies were obtained with solvents **C** and **D** (nine analytes each) in the *A. cygnea* and with solvents **B** (nine analytes) and **A** (eight analytes) for *P. clarkii*. Finally, pesticides obtained the lowest extraction efficiency. In the *P. clarkii*, recovery of 33 analytes was achieved with solvent B and 28 analytes with solvent **A**, whereas for the *A. cygnea*, solvents **A** and **D** recovered the highest number of analytes (27 each one). In general terms, solvent **A** and **B**´s extraction efficiencies are acceptable for most compounds assessed. In this sense, we decided to select solvent **A** (**MeCN + 0.1% F.A**) to continue with the clean-up test because the calculated RSDs for most of the analytes are below 20% for the two matrices. In contrast, solvent **B** showed higher extraction uncertainty among the triplicates evaluated.

### Clean-up sorbent optimization

**Fig. 2:**
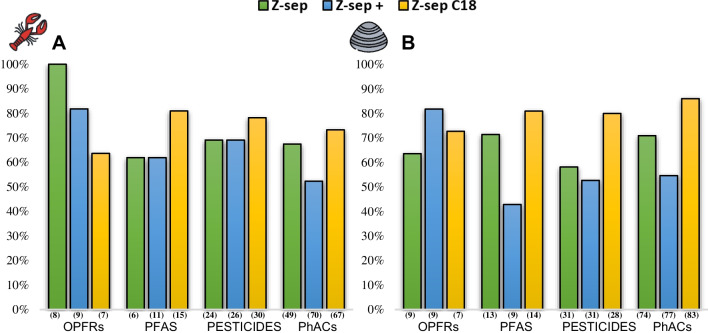
Clean-up step test results. **A** *P. clarkii.* **B** *A. cygnea.* Bars indicate the percentage of compounds with an extraction efficiency between 40 and 120%. The percentage was calculated according to the total number of analytes in each compound group: OPRFs (11), PFAS (21), pesticides (54), and PhACs (87). (Numbers in brackets): analytes with RSD ≤ 20%.

One of the main challenges during extraction method development for complex biota matrices is the co-extraction of matrix compounds such as lipids, proteins, and other compounds that may interfere with the signal of the target analytes. A clean-up step was added after the extraction stage to avoid these interferences during analysis. Three different sorbents were tested: **A** Z-Sep, **B** Z-Sep^+^, and **C** Z-Sep/C_18_. The percentage of compounds for which an acceptable recovery efficiency (40 to 120%) was obtained is shown in Fig. 2. For PFAS, pesticides, and PhACs, most compounds were recovered after the cleaning step with sorbent **C**, in both *P. clarkii* and *A. cygnea*. For OPFRs, there were differences. In *P. clarkii*, the best results were obtained with sorbent **A**, whereas in *A. cygnea*, the best results were with sorbent **B**. However, satisfactory recoveries were obtained with sorbent **C**, with RSD below 20% (Table S3). The number of compounds recovered within an acceptable range was increased by including the extracts clean-up with the sorbent **C**, mainly for the pesticide group, where 12 more analytes were retrieved in the *P. clarkii* and 15 more in the *A. cygnea*.

During this experiment, we evaluated the percentage of matrix removed by each Z-sep tested. For this, we compared the amount of matrix remaining after extraction with MeCN + 0.1% F.A with the amount removed after the cleaning step. Thus, sorbent **A** reduced 14% of the matrix interferents in *A. cygnea* and 24% in *P. clarkii*. Sorbent **B** reduced 55% of the matrix in both species, and finally, sorbent **C** was able to eliminate 42% of interferents in *A. cygnea* and 17% in *P. clarkii*. The higher percentage obtained with sorbent **B** is probably due to the fact that this sorbent also favored the elimination of the pigments present in the extracts.

Overall, the use of cleanup generated a reduction in enhancement of several compounds during solvent testing by increasing the number of compounds within the ME range considered acceptable (>−40% and <40%) (Fig. S1). In the case of OPFRS, this effect was greater with sorbent **B** in *P. clarkii* and sorbent **C** in *A. cygnea*. Likewise, for the PhACs, the cleanup with sorbent **B** increased the number of compounds within the acceptable range of ME, going from 53 analytes in the solvent test to 63 for *P. clarkii* and 59 to 85 in *A. cygnea*. PFAS and pesticides obtained the best results with sorbent **C** for both species. Sorbent** C** included three more compounds in *P. clarkii* within the acceptable range and nine more in *A. cygnea.*

Another essential criterion for choosing the cleaning sorbent depends on the fat content of the matrix to be analyzed. We calculated the fat percentage of the four species according to [41]. Thus, we obtained that the fat rate (w.w) for *A. cygnea* was 2.2%, 2.3% for *L. gibossus*, 3% for *P. clarkii*, and 0.7% for *C. fluminea*. Relating to the results obtained, we decided to include Z-sep/C_18_ in the cleaning step because the sorbent allowed the recovery of the highest number of compounds in all matrices. Additionally, Z-sep/C_18_ obtained the lowest RSD values when calculating the recovery efficiencies and allowed the reduction of the matrix interferences without compromising the recoveries. The satisfactory results obtained for the Z-sep/C_18_ sorbent coincide with the expectations, considering that the percentage of fat calculated for the species is less than 15%.

### Method validation

Validation of the optimized extraction method, i.e., using **MeCN + 0.1% F.A** as extraction solvent and **Z-Sep/C**_**18**_ sorbent during the clean-up step, was performed to confirm whether the procedure allows reliable analytical results. Validation was performed by evaluating the accuracy, linearity, precision, LOD, and LOQ defined in the “Validation protocols” section. The method was validated for the four species of interest: *P. clarkii, A. cygnea, C. fluminea*, and *L. gibbosus*. Accuracy and precision were calculated from three repeated injections for each kind of matrix spiked at 10 ng g^−1^ and 100 ng g^−1^ (w.w).

#### Recoveries and robustness

**Fig. 3:**
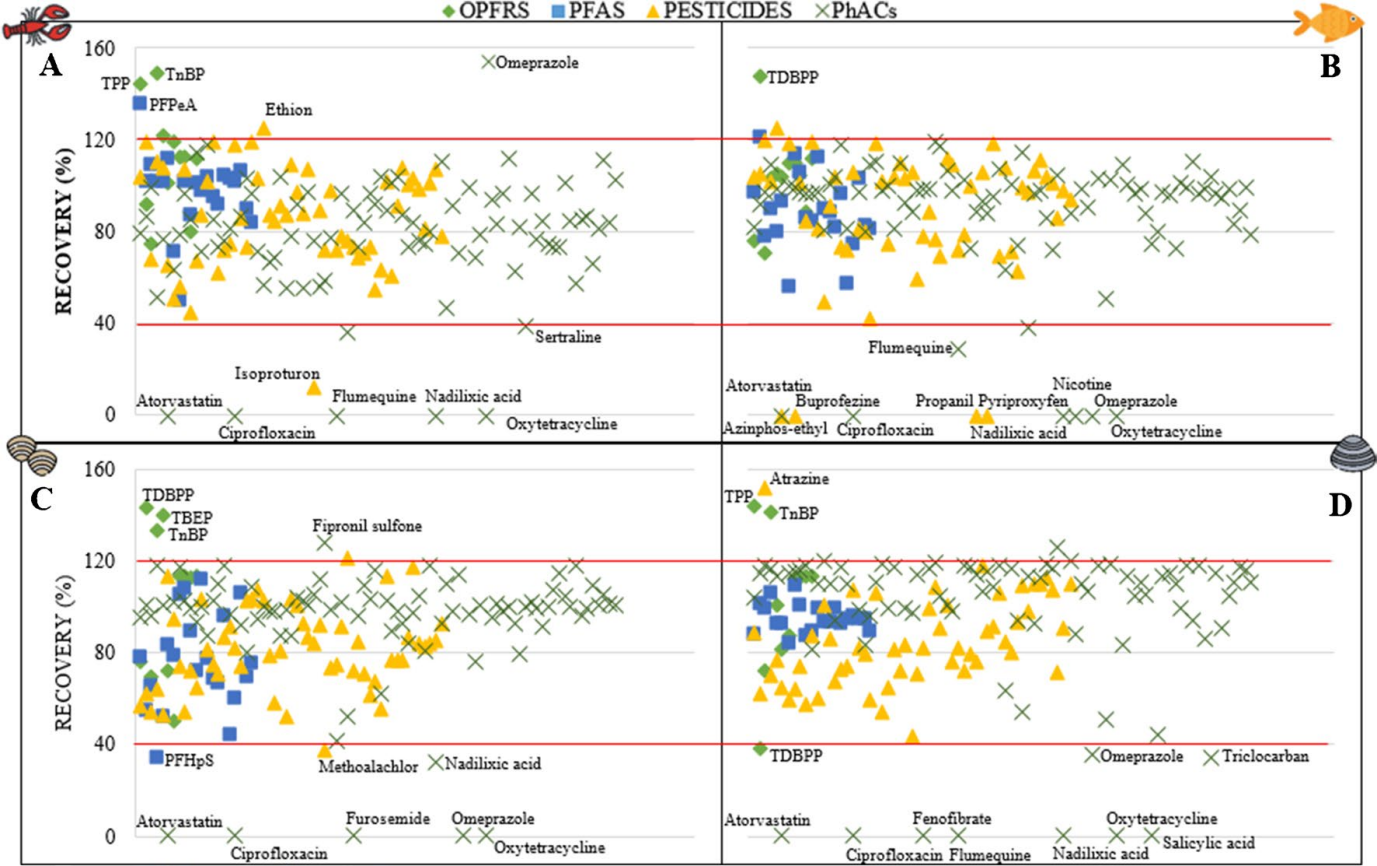
Recoveries in all matrices for level 2: 100 ng g^−1^. **A*** P. clarkii*, **B** *L. gibossus,* **C** *C. fluminea*, **D** *A*. *cygnea*. Compounds between the red lines obtained recoveries within the acceptable range (40–120%). The names of the compounds outside the acceptable range at the two validation levels are shown.

To define the compounds that can be reliably detected with the developed extraction method, acceptability criteria were established, based on the recovery and RSD values calculated during validation. Thus, detecting an analyte was considered reliable if the recovery was between 40 and 120% and its RSD was <25% in at least one of the validation levels. Table S4 shows the calculated values. Fig. 3 summarizes the recoveries by compound group and matrix.

**Fig. 4:**
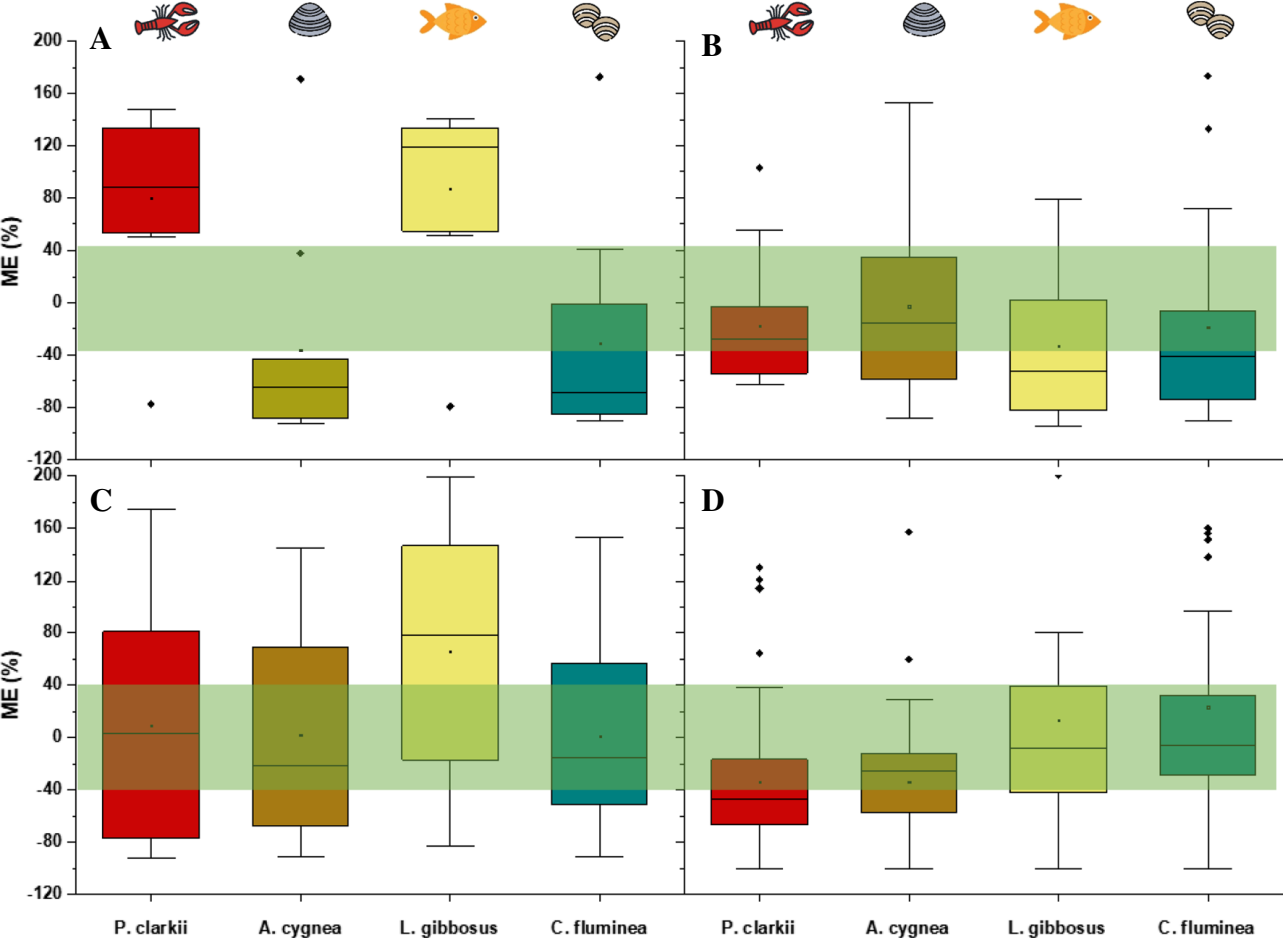
Matrix effects for level 1 of validation (10 ng g^−1^). **A**. OPFRs, **B**. PFAS, **C**. Pesticides, **D**. PhACs. The green band shows the acceptable ME range (-40% to 40%).

Seven of the 11 validated OPFRS (green diamonds) were recovered with the established acceptability criteria in all four species. The other four OPFRS are outside the criteria in at least one species, mainly because their recovery is higher than 120% (TPP, the TnBP, the TDBPP, and the TBEP). Recoveries higher than the established maximum are probably due to an enhancement of the analyte response due to co-extractants (matrix effect). This effect is more marked in *P. clarkii* and *L. gibossus* (Fig. 4). Similar results have been obtained for the TPP in eel [25].

Of the 21 validated PFAS (blue boxes), only two are outside the acceptability criteria: PFPeA in *P. clarkii* with a recovery greater than 120% and PFHpS in *C. fluminea* with a recovery less than 40%. We consider that these two particular cases depend exclusively on the interaction of the matrix with the analyte at the time of ionization since their recoveries were satisfactory in the other species. In the case of PFPeA, the calculated ME in *P. clarkii* was an outlier in the data series (146%). The increase of its signal after ionization may have induced its high recovery (136%). The opposite effect occurred for PFHpS in *C. fluminea* (*R*, 34% and ME, −52%).

Regarding the pesticides (yellow triangles), 46 of the 54 compounds validated were within the range of acceptability in all species. Eight pesticides were outside the field, but each for a specific species. Ethion and isoproturon obtained recoveries greater than 120% and less than 40%, respectively, only in *P. clarkii.* The recovery of atrazine was above the acceptable range for *A. cygnea* and metolachlor below the range for *C. fluminea*. In *L. gibossus*, the buprofezine, azinphos-ethyl, propanil, and pyriproxyfen were not recovered at either validation level.

Finally, 75 of the 87 validated PhACs (green crosses) met the quality criteria. Five PhACs were not recovered in any species: atorvastatin, ciprofloxacin, nalidixic acid, oxytetracycline, and omeprazole. Flumequine was only recovered within the acceptable range in *C. fluminea*. Sertraline and nicotine recovered below 40% in *P. clarkii* and *L. gibossus*, respectively. Fipronil sulfone and furosemide were not validated in *C. fluminea*. Fenofibrate and salicylic acid were not recovered in *A. cygnea*. The zero-recovery rate during validation of ciprofloxacin, nalidixic acid, and oxytetracycline was related to their high polarity (log *P*, −0.87, 1.01, and −4.87, respectively) (Table S1). This condition allowed them to be recovered with greater than 50% efficiency with H_2_O:MeOH (7:3) + 1% F.A in *P. clarkii* and *C. fluminea* during the solvent tests (Table S4). However, prioritizing MeCN + 0.1% F.A due to its better results for most of the analytes, the recovery of these latter compounds was compromised.

#### Matrix effects

During the analysis, matrix components present in the extracts can affect the quantification of the target analytes by increasing or suppressing their signal under ESI conditions in biological sample extracts [42]. The water content of *P. clarkii*, *A. cygnea*, *C. fluminea*, and *L. gibbosus* is close to 90% of its body weight with a fat content of 11.8%, 10.9%, 6.2%, 9.4% on a dry matter basis, respectively. In addition, their tissues, especially clams, are rich in proteins, carbohydrates including mucoproteins, glycoproteins, glycolipids or sphingolipids, and acid mucopolysaccharides, and pigments which carry out an intense chemical and biological activity. Unfortunately, all of these matrix components are inevitably co-extracted and present in the final extracts and are prone to altering the ionization efficiency of the analytes in the ion source. Although in most cases, this leads to suppression of the signal, compared to that obtained for a standard solution at the same concentration, in the case of OPFRS in *P. clarkii*, and in *L. gibbosus*, it is possible to observe an enhancement. According to [42], the higher ME could be related to the coelution of lipophilic matrix components, which apparently had not been completely removed during cleanup procedure. The pesticides showed a greater dispersion of the ME calculated in all matrices. In this case, no clear trend is evident in either ion polarity. Furthermore, this variation in ME values could be attributed to the relatively high log *P* variability of the pesticides (0.13–7.05). On the contrary, PFAS and PhACs tended to have more similar values despite the large log *P* variability especially of PhACs (−4.87 to 5.62). In these two groups, there was a higher percentage of analytes with an ME between −40 and 40% in most matrices, which implies a lower sensitivity to matrix interferences. Forty-three percent of all validated compounds obtained ME values between −40 and 40% in the *C. fluminea*, which means that in this species, the influence of the matrix tends to be lower than in the other species analyzed. ME calculated for each group of compounds and in each of the matrices are summarized in Fig. 4 whereas they are individually detailed in Table S4.

#### Linearity, LOD, and LOQ

The LOD and LOQ of the method were calculated from the calibration curve of each matrix using Eqs. (3) and (4). Pesticides were the compounds with the highest LOQ values for some of the compounds (>14 ng g^−1^), while for PFAS, the calculated limits reached lower concentrations (<9 ng g^−1^) for most of the analytes. The LODs of OPFRs ranged from 0.04 to 4 ng g^−1^ and LOQs ranged from 0.1 to 12 ng g^−1^. The PhACs obtained LOD values between 0.01 and 7 ng g^−1^, and LOQ between 0.02 and 22 ng g^−1^. The LOD and LOQ for each group of compounds in each matrix are shown in Table S4.

### Comparison with other extraction methods

Table 2 shows the LOQs, recoveries, and RSDs of PhACs, pesticides, OPFRs, and PFASs obtained with different extraction methods based on UAE. The analytical values obtained with the method optimized by us are comparable with those previously reported for analysis in aquatic biota. However, the advantage offered by our method is the possibility of extracting a larger number of compounds from different chemical families with the same extraction solvent. The clean-up used does not compromise the recoveries of the analytes and it is a fast and simple step. Additionally, our method meets the requirements of green chemistry by using only 1 mL of solvent while most reported methods use more than 5 mL. We have successfully developed a single extraction method for the simultaneous analysis of CECs in four different matrices.

**Table 2:** Comparison with other UAE methods

Solvent and clean-up used in UAE method	Matrix	Validated compounds	LOQ (ng g^−1^, w.w)	Recovery (%)	RSD (%)	Reference
Solvent: MeCN/MeOH + acetic acid-ammonium acetate (1 mL) Clean-up: HybridSPE®-phospholipid cartridge	Fish homogenate	72 PhACs and Personal care products	0.02–4.3	38–120	0.2–20	[29]
Solvent: Ice-cold MeCN/MeOH (1/1) (5 mL) Clean-up: Oasis HLB cartridge	Plasma, liver, brain fish	17 PhACs and Personal care products	0.02–8.7	90–110	1.2–10	[31]
Solvent: MeCN/H_2_O (8:2) (8 mL) Clean-up: Oasis PRiME HLB cartridge	Fish mussel and whole-body shellfish	34 PhACs and Personal care products	0.03–5.6	43–127	-	[32]
Solvent: n-heptane + MeCN (9 mL) Clean-up: silica gel SPE cartridge	Fish muscle and liver	63 PhACs and their metabolites	0.1–48.2	69–133	-	[22]
Solvent: MeCN/H_2_O (3/1) + 0.1% acetic acid (2 mL) Clean-up: Strata Alumina-N cartridge coupled to an Oasis HLB cartridge	Crustacean	67 PhACs and pesticides	0.1–25	43–100	7–22	[28]
Solvent: EtAct/MeCN (1/1) + 10 g anhydrous sodium sulfate (20 mL) Clean-up: GPC clean-up and d-SPE	Fish muscle	33 pesticides	0.001–0.04	57–110	2–20	[23]
Solvent: hexane/acetone (1/1) (15 mL) Clean-up: none	Fish	14 OPFRs	0.07–7	47–98	2.4–16	[24]
Solvent: MeOH (10 mL) Clean-up: STRATA-X cartridge	Fish homogenate	9 OPFRs	0.02–0.3	47–123	1–23	[25]
Solvent: hexane:acetone (1/1) (15 mL) Clean-up: SPE (tandem basic alumina with C18)	Dried fish	16 OPFRs	0.34–12	45–115	1–25	[43]
Solvent: H_2_O/MeCN (1/9) (1.5 mL) Clean-up: HybridSPE® Phospholipid Ultra cartridge	Mussels, clams, fish	11 PFAS	0.1–0.9	40–133	4–19	[27]

### Quantification of CECs in real biota samples

The developed method was successfully applied to analyze the target analytes in three invasive species captured in the Albufera Natural Park (Valencia, Spain). We quantified using matrix-matched calibration curves for each matrix and the internal standard method described in the “LC-HRMS/MS analysis” section. Only compounds with reliable values during validation, i.e., those with recoveries between 40 and 120% and RSD < 25%, were included in the analysis (Table S4).

Table 3 shows the CECs detected in at least one of the samples analyzed. Thirty-five PhACs, 21 pesticides, 20 PFAS, and two OPFRs were detected. The compounds with the highest mean concentrations were the PFAS perfluorotridecanoic acid (PFTrDA) (191 ng g^−1^ in P*. clarkii*) and perfluoro-n-tetradecanoic acid (PFTeDA) (86 ng g^−1^ in *L. gibbosus*). The pesticide fenthion had the highest concentration in *C. fluminea* (52 ng g^−1^). PhACs were detected at concentrations <10 ng g^−1^ (w.w).

**Table 3:** Compounds detected in the invasive species analyzed. Concentrations are expressed in ng g^−1^ (w.w). *n*, number of samples analyzed by species; *<LOQ*, less than limit of quantification; *Mean*, average concentration was calculated based on positive samples; for samples with data <LOQ, the value of the calculated limit of quantification divided by 2 was used; *Max*, maximum concentration calculated; *DF*, detection frequency

CECs		*P. clarkii* *n=17*				*L. gibbosus* *n=7*				*C. fluminea* *n=8*			
		Mean (ng g^−1^)	Median (ng g^−1^)	Max (ng g^−1^)	DF	Mean (ng g^−1^)	Median (ng g^−1^)	Max (ng g^−1^)	DF	Mean (ng g^−1^)	Median (ng g^−1^)	Max (ng g^−1^)	DF
PhACs	5-Methyl-1H-Benzotriazole	<LOQ	0.5	<LOQ	76%	<LOQ	<LOQ	<LOQ	100%	<LOQ	<LOQ	<LOQ	100%
	Acetaminophen	<LOQ	1.1	10	100%	2	0.3	8	100%	<LOQ	<LOQ	<LOQ	100%
	Benzotriazole	<LOQ	1.1	<LOQ	12%	<LOQ	<LOQ	<LOQ	100%	<LOQ	<LOQ	<LOQ	100%
	Benzoylecgonine	-	-	-	-	-	-	-	-	<LOQ	<LOQ	<LOQ	38%
	Bisphenol-A	-	-	-	-	<LOQ	<LOQ	<LOQ	86%	5	4	11	100%
	Caffeine	6	1	25	88%	<LOQ	<LOQ	<LOQ	100%	<LOQ	0.5	<LOQ	100%
	Carazolol	<LOQ	0.8	<LOQ	18%	-	-	-	-	-	-	-	-
	Carbamazepine	<LOQ	0.4	<LOQ	29%	-	-	-	-	<LOQ	<LOQ	<LOQ	38%
	CBZ-10,11-epoxide	<LOQ	0.2	<LOQ	41%	-	-	-	-	<LOQ	<LOQ	<LOQ	13%
	Citalopram	-	-	-	-	-	-	-	-	0.1	0.1	0.3	88%
	Cocaine	1	0.5	4	35%	<LOQ	<LOQ	<LOQ	29%	<LOQ	<LOQ	<LOQ	100%
	Cotinine	6	3	37	100%	<LOQ	<LOQ	<LOQ	57%	1	0.3	8	100%
	Diltiazem	<LOQ	-	<LOQ	18%	-	-	-	-	<LOQ	<LOQ	<LOQ	100%
	Fluconazole	<LOQ	0.1	0.3	29%	-	-	-	-	-	-	-	-
	Fluoxetine	<LOQ	-<LOQ	<LOQ	6%	-	-	-	-	<LOQ	0.3	0.7	100%
	Hydrochlorothiazide	<LOQ	-	<LOQ	100%	<LOQ	<LOQ	<LOQ	100%	<LOQ	0.1	0.4	100%
	Ketoprofen	-	-	-	-	7.4	7.4	7.4	14%	-	-	-	-
	Methadone	<LOQ	-	<LOQ	6%	-	-	-	-	<LOQ	<LOQ	<LOQ	50%
	N-Desmethylcitalopram	<LOQ	0.6	<LOQ	6%	-	-	-	-	<LOQ	<LOQ	<LOQ	13%
	Nicotine	-	-	-	-	-	-	-	-	<LOQ	<LOQ	<LOQ	100%
	O-Desmethylvenlafaxine	0.4	0.3	1.5	94%	-	-	-	-	<LOQ	0.1	<LOQ	100%
	Paroxetine	-	-	-	-	-	-	-	-	<LOQ	<LOQ	<LOQ	38%
	Pentobarbital	9	9	17	47%	3	0.3	12	71%	3	3	3.5	100%
	Propyphenazone	<LOQ	<LOQ	<LOQ	18%	-	-	-	-	-	-	-	-
	Quetiapine	-	-	-	-	-	-	-	-	<LOQ	<LOQ	<LOQ	75%
	Salbutamol	<LOQ	0.3	0.6	82%	-	-	-	-	<LOQ	<LOQ	<LOQ	63%
	Salicylic acid	-	-	-	-	5	3.2	18	100%	5	5	9.5	100%
	Sertraline	-	-	-	-	-	-	-	-	7	6.4	8	100%
	Sulfadimethoxine	<LOQ	0.2	<LOQ	6%	-	-	-	-	-	-	-	-
	Sulfapyridine	<LOQ	0.3	<LOQ	6%	-	-	-	-	-	-	-	-
	Tramadol	<LOQ	0.4	<LOQ	100%	<LOQ	0.2	<LOQ	100%	0.6	0.6	0.9	100%
	Trimethoprim	<LOQ	0.7	<LOQ	18%	-	-	-	-	3	3	3	13%
	Valsartan	1	1	1	24%	-	-	-	-	-	-	-	-
	Venlafaxine	<LOQ	0.3	<LOQ	94%	-	-	-	-	<LOQ	0.1	<LOQ	100%
	Zolpidem	<LOQ	<LOQ	<LOQ	6%	-	-	-	-	-	-	-	-
OPFR	TMPP	<LOQ	0.2	0.3	24%	-	-	-	-	-		-	-
	TPP	-	-	-	-	-	-	-	-	<LOQ	<LOQ	<LOQ	13%
PFAS	iPFNS	-	-	-	-	-	-	-	-	37	37	37	25%
	IpPFNA	<LOQ	<LOQ	<LOQ	12%	<LOQ	<LOQ	<LOQ	86%	<LOQ	2	5	83%
	PFBA	6	4.8	13	94%	6	6	9	100%	-	-	-	-
	PFBS	1	1	2.5	29%	0.8	0.9	1	86%	2	2	2.5	100%
	PFDA	2	1.4	3.5	82%	0.7	0.8	1	100%	0.8	1	1	75%
	PFDoDA	21	21	21	12%	<LOQ	<LOQ	<LOQ	29%	-	-	-	-
	PFDS	-	-	-	-	18	18	20	57%	20	15	41	50%
	PFHpA	0.4	0.3	0.9	65%	<LOQ	<LOQ	<LOQ	43%	<LOQ	0.8	<LOQ	63%
	PFHpS	10	4.5	27	41%	22	22	22	14%	-	-	-	-
	PFHxA	<LOQ	0.5	2	82%	<LOQ	2	4	86%	6	5	16	88%
	PFHxDA	<LOQ	<LOQ	<LOQ	41%	22	17	43	86%	<LOQ	0.9	<LOQ	50%
	PFHxS	2	1.8	5	94%	0.9	0.9	1.5	100%	0.06	0.02	0.2	88%
	PFNA	<LOQ	<LOQ	<LOQ	94%	-	-	-	-	<LOQ	<LOQ	<LOQ	100%
	PFOA	2	1.8	3	94%	12	9.6	28	100%	<LOQ	0.1	0.2	88%
	PFODA	73	73	74	35%	13	13	13	43%	-	-	-	-
	PFOS	17	9.7	60	88%	3.5	1	19	100%	<LOQ	<LOQ	<LOQ	100%
	PFPeA	-	-	-	-	<LOQ	<LOQ	<LOQ	100%	-	-	-	-
	PFTeDA	-	-	-	-	86	86	91	29%	-	-	-	-
	PFTrDA	191	195	200	24%	13	12.6	13	29%	-	-	-	-
	PFUnDA	0.5	0.2	2	76%	<LOQ	0.1	0.2	86%	<LOQ	<LOQ	<LOQ	88%
Pesticides	Acetamiprid	-	-	-	-	<LOQ	5	7	100%	<LOQ	3.6	15	88%
	Alachlor	-	-	-	-	<LOQ	<LOQ	<LOQ	14%	4	4	4	13%
	Atrazine-desisopropryl	<LOQ	<LOQ	<LOQ	29%	<LOQ	<LOQ	<LOQ	43%	<LOQ	<LOQ	<LOQ	13%
	Carbendazim	<LOQ	<LOQ	<LOQ	12%	1	0.5	4	100%	<LOQ	<LOQ	<LOQ	100%
	Carbofuran-3-hydroxy	-	-	-	-	5	4.7	8	43%	12	12	19	100%
	Diazinon	-	-	-	-	<LOQ	<LOQ	<LOQ	14%	-	-	-	-
	Dichlofenthion	42	42	43	29%	-	-	-	-	-	-	-	-
	Diuron	-	-	-	-	<LOQ	<LOQ	<LOQ	29%	<LOQ	<LOQ	<LOQ	75%
	DMA	<LOQ	7	15	18%	17	17	19	43%	15	14	18	88%
	Etofenprox	-	-	-	-	<LOQ	<LOQ	<LOQ	29%	-	-	-	-
	Fenthion	51	51	51	6%	51	52	52	100%	52	52	52	100%
	Imidacloprid	<LOQ	2	2	65%	-	-	-	-	-	-	-	-
	Methiocarb	24	24	24	6%	24	24	24	86%	24	24	24	100%
	Molinate	<LOQ	0.8	0.8	24%	-	-	-	-	<LOQ	<LOQ	<LOQ	13%
	Propanil	<LOQ	<LOQ	<LOQ	12%	-	-	-	-	<LOQ	<LOQ	<LOQ	13%
	Propazine	<LOQ	<LOQ	<LOQ	18%	-	-	-	-	-	-	-	-
	Spinosyn A	6	6	6	35%	-	-	-	-	-	-	-	-
	Spinosyn D	-	-	-	-	-	-	-	-	3	3	3	13%
	Tebuconazole	<LOQ	<LOQ	<LOQ	6%	<LOQ	2	<LOQ	86%	<LOQ	<LOQ	<LOQ	25%
	Terbuthylazine-2-hydroxy	<LOQ	<LOQ	<LOQ	12%	-	-	-	-	<LOQ	<LOQ	<LOQ	50%
	Terbutryn	8	8	8	12%	8	8	8	43%	-	-	-	-

The presence of PFTrDA and PFTeDA has been previously reported in *Perca fluviatillis* liver (22 ng g^−1^ and 12 ng g^−1^, respectively) [5]. The results for PFTrDA were similar to those obtained by us in *L. gibbosus* homogenate (13 ng g^−1^), but it is higher for PFTeDA. Propanil is a widely used herbicide in rice crops, is susceptible to biodegradation, is not persistent, and is easily transformed into various sub-products [44]. Salmo gairdneri has been shown to readily metabolize propanil forming at least ten sub-products [45]. Its presence in the analyzed samples does not suggest a harmful effect. On the other hand, fenthion is a broad-spectrum organophosphate pesticide used on numerous crops. The U.S. Environmental Protection Agency (EPA) has classified it as a restricted-use pesticide due to its toxic effects on birds, reptiles, and fish [46]. A significant decrease in protein was demonstrated in the middle mantle of *Lamellidens corrianus* when exposed to nominal (7 ng g^−1^) and lethal (14 ng g^−1^) concentrations of fenthion [47]. This compound was detected in 100% of the *L. gibbosus* and *C. fluminea* samples at concentrations above lethal concentrations. The concentrations detected suggest its general use in rice crops present in the Albufera, since it is a pesticide that allows the control of the rice borer (*Chilo suppressalis*), which is one of the most important pests in the rice fields of Valencia [48]. In this context, it would be interesting to extend the studies of fenthion bioaccumulation in these species.

In the case of PhACs, similar concentrations of acetaminophen, caffeine, carbamazepine, and fluoxetine have been previously reported in *P. clarkii* and clams *Ruditapes decussatus* caught in their habitat [17, 47]. PhACs such as acetaminophen (widely used over-the-counter analgesic); hydrochlorothiazide (used for the hypertension treatment); and tramadol (opioid painkiller) were the compounds with the highest detection frequency, detected in 100% of the samples analyzed.

As regards C*. fluminea,* 24 of the 78 compounds reported in Table 3 were detected in all samples of this species, but most of them were not quantifiable. The compounds with the highest mean concentrations in this species were fenthion (52 ng g^−1^), sertraline (7 ng g^−1^), and PFBS (2 ng g^−1^). The high detection frequency in this species may be related to its ability to filter water and its anatomy, which could facilitate the absorption of compounds. *C. fluminea* has been shown to have a high accumulation rate of contaminants such as heavy metals [49]. Due to their biofiltration capacity, they have been able to eliminate up to 30% of carbamazepine and lorazepam concentrations during PhACs removal trials from wastewater [50]. Although *C. fluminea* was the species with the highest detection frequency, *P. clarkii* reported the highest maximum concentrations, especially for the PFAS group, so biomonitoring of this class of compounds with this species would be pretty interesting for future studies.

## Conclusions

An ultrasound-assisted extraction with MeCN + 0.1% F.A as solvent, followed by a clean-up step with Z-sep/C_18_, and subsequent quantification by HPLC-MS/MS provided the best compromise of high precision and matrix effects for the studied CECs in invasive species in a coastal wetland. The method allowed the simultaneous extraction of four groups of contaminants with different physicochemical properties (PhACs, pesticides, PFASs, and OPFRs) in different species. This method proved suitable for the correct recovery of most compounds with a low RSD. The application of the method allowed the detection of 78 compounds from different CECs families in freshwater species samples. The results obtained for *C. fluminea* and *P. clarkii* showed their capacity to accumulate contaminants and its potential to be used in biomonitoring studies.

### Supplementary Information

Below is the link to the electronic supplementary material.

Supplementary file1 (PDF 291 KB)

## Declarations

### Source of biological material

Samples were provided by the Fish Research Centre “El Palmar,” Generalitat Valenciana (Valencia, Spain).

### Statement on animal welfare

No applications are required for testing with invertebrates. Fish experiments were performed according to the “Directive 2010/63/EU of the European Parliament and of the Council of 22 September 2010 on the protection of animals used for scientific purposes” and the Spanish legislation (Royal Decree 53/2013, from the 1st of February, which establishes the basic regulations that are applicable for the protection of animals used for experiments and other scientific uses, including teaching).
