# Supplementary material for: An effective method for the simultaneous extraction of 173 contaminants of emerging concern in freshwater invasive species and its application
Source: Anal Bioanal Chem. 2023 Sep 30;415(29-30):7085–101. doi: 10.1007/s00216-023-04974-3 (PMC10684701; doi:10.1007/s00216-023-04974-3)
Supplement: Supplementary file 1 — Supplementary file1 (PDF 291 KB) [file 216_2023_4974_MOESM1_ESM.pdf]

SUPPLEMENTARY INFORMATION

Table S1.1. Pharmaceuticals (PhACs) list. 2

Table S1.2. Organophosphorus flame retardants (OPFRs) list. 4

Table S1.3. Pesticides list. 4

Table S1.4. Perfluoroalkyl substances (PFAS) list. 5

Table S2.1. Quality parameters for quantification purposes of PhACs 7

Table S2.2. Quality parameters for quantification purposes of OPFRs 9

Table S2.3. Quality parameters for quantification purposes of Pesticides. 9

Table S2.4. Quality parameters for quantification purposes of PFAS 11

Table S3. Solvent and clean-up test results 13

Table S4. Validation parameters 15

Fig S1. Matrix effect before and after the clean-up step 18

Table S1.1. Pharmaceuticals (PhACs) list. CE: Collision energy; RT: Retention time.

PhACs	Mode	Precursor mass (*m/z*)	Confirming fragment (*m/z*)	CE (V)	RT (min)	Log P
5-methyl-1H-Benzotriazole	+	134.0713	79.0542	25	7.29	1.81
Acetaminophen	+	152.0706	110.0600	20	2.94	0.91
Acridone	+	196.0757	167.0721	30	10.41	4.20
Alprazolam	+	309.0901	281.0713	37	12.56	3.02
Amantadine	+	152.1434	135.1167	15	4.69	1.47
Atenolol	+	267.1703	190.0861	25	2.67	0.43
Atorvastatin	-	557.2457	278.1350	50	13.12	5.39
Benzotriazole	+	120.0556	65.0386	15	5.00	1.30
Benzoylecgonine	+	290.1387	168.1018	26	5.65	-0.59
Bezafibrate	+	362.1154	316.1096	14	13.41	3.99
Bisphenol-A	-	227.1078	211.0760	31	12.96	4.04
Bromazepam	+	316.0080	209.0945	31	10.42	2.54
Caffeine	+	195.0877	138.0661	27	4.48	-0.55
Carazolol	+	299.1754	116.1068	40	8.74	2.71
Carbamazepine	+	237.1022	194.0963	23	11.70	2.77
CBZ-10.11-epoxide	+	253.0972	180.0806	20	9.46	1.97
Chloramphenicol	-	321.0051	152.0353	15	8.93	0.88
Chlorpromazine	+	319.1030	86.0964	19	12.49	4.54
Ciprofloxacin	+	332.1405	288.1506	25	5.76	-0.81
Citalopram	+	325.1711	109.0446	26	10.78	3.76
Clarithromycin	+	748.4841	158.1174	34	12.43	3.24
Clofibric acid	-	213.0324	126.9955	30	6.91	2.90
Coca-ethylene	+	318.1700	196.1330	25	8.76	2.64
Cocaine	+	304.1543	182.1174	30	7.34	2.28
Codeine	+	300.1594	165.0697	42	3.50	1.34
Cotinine	+	177.1022	80.0494	28	1.70	0.21
Diazepam	+	285.0789	193.0885	35	13.69	3.08
Diclofenac	-	294.0094	221.1544	-	12.31	4.26
Diltiazem	+	415.1686	178.0319	24	11.24	2.73
Erythromycin	+	734.4685	158.1174	29	11.29	2.60
Fenofibrate	+	361.1201	233.0364	12	16.07	5.28
Fipronil	-	434.9314	249.9580	20	14.97	4.49
Fipronil Desulfynil	-	386.9644	350.9877	10	15.14	4.53
Fipronil sulfide	-	418.9365	261.9570	25	15.36	5.62
Fipronil sulfone	-	450.9263	183.0270	25	15.34	4.60
Fluconazole	+	307.1113	310.1219	20	6.87	0.56
Flumequine	+	262.0874	244.0766	32	11.93	2.42
Fluoxetine	+	310.1413	265.1577	30	12.42	4.17
Furazolidone	+	226.0459	95.0366	18	6.18	0.87
Furosemide	-	329.0004	204.9845	-	7.58	1.75
Hydrochlorothiazide	-	295.9572	268.9455	29	4.14	-0.58
Indomethacin	-	356.0695	297.0561	-	12.55	3.53
Ketoprofen	+	255.1016	105.0334	25	13.23	3.61
Lamotrigine	+	256.0151	172.9667	40	6.12	1.93
Loratadine	+	383.1521	337.1099	-	14.30	5.20
Lorazepam	+	321.0192	275.0138	19	12.48	4.06

PhACs	Mode	Precursor mass (*m/z*)	Confirming fragment (*m/z*)	CE (V)	RT (min)	Log P
Losartan	+	423.1695	207.0917	20	12.95	4.06
Mefenamic acid	-	240.1030	196.1134	19	13.12	5.40
Metformin	+	130.1087	60.0560	15	1.14	-0.92
Methadone	+	310.2165	265.1587	18	12.29	5.01
Metoprolol	+	268.1907	116.1068	21	6.54	1.76
Midazolam	+	326.0855	291.1166	38	10.30	3.97
Morphine	+	286.1438	201.0909	40	1.86	0.90
N-acethyl SMX	+	296.0700	134.0600	24	8.81	0.86
Nalidixic acid	+	233.0921	205.0606	32	11.37	1.01
N-Desmethylcitalopram	+	311.1554	109.0447	-	10.55	3.38
Nicotine	+	163.1230	117.0573	22	1.44	1.17
Norfluoxetine	+	296.1257	105.0700	18	12.28	3.70
O-Desmethylvenlafaxine	+	264.1958	246.1851	21	5.68	2.29
Omeprazole	+	346.1220	198.0583	-	9.28	2.43
Oseltamivir	+	313.2122	166.0864	12	8.78	1.16
Oseltamivir-CBX	+	285.1809	138.0551	38	5.19	-1.84
Oxazepam	+	287.0582	241.0524	25	12.21	2.92
Oxytetracycline	+	461.1555	426.1171	35	5.39	-4.87
Paroxetine	+	330.1500	192.1183	27	11.86	3.15
Pentobarbital	-	225.1245	125.9001	30	11.47	1.89
Propyphenazone	+	231.1492	189.1021	30	12.24	2.35
Quetiapine	+	384.1740	253.0794	30	10.36	2.81
Salbutamol	+	240.1594	148.0756	-	2.37	1.40
Salicylic acid	-	137.0244	93.0345	16	2.99	1.98
Sertraline	+	306.0811	158.9762	20	12.47	5.15
Sitagliptin	+	408.1254	174.0524	24	7.70	1.26
Sotalol	+	273.1268	213.0690	24	2.43	-0.40
Sulfadimethoxine	+	311.0809	156.0771	-	10.04	0.23
Sulfamethazine	+	279.0910	204.0436	22	5.98	0.65
Sulfamethoxazole	+	254.0594	156.0115	20	7.84	0.79
Sulfapyridine	+	250.0645	156.0112	22	4.60	1.01
Temazepam	+	301.0738	255.0684	-	12.99	2.19
Tramadol	+	264.1958	58.0654	15	6.50	2.45
Triclocarban	-	312.9708	159.9724	35	15.35	4.93
Trimethoprim	+	291.1452	230.1161	29	4.83	1.28
Valsartan	+	436.2343	207.0915	15	13.68	4.59
Valsartan acid	-	265.0731	165.0706	35	3.95	2.56
Venlafaxine	+	278.2115	58.0654	22	8.39	2.74
Verapamil	+	455.2904	165.0909	27	12.26	5.04
Warfarin	-	307.0976	161.0244	24	11.58	2.74
Zolpidem	+	308.1757	235.1230	30	8.16	3.02

Table S1.2. Organophosphorus flame retardants (OPFRs) list. CE: Collision energy; RT: Retention time.

OPFRs	Mode	Precursor mass (*m/z*)	Confirming fragment (*m/z*)	CE (V)	RT (min)	Log P
TCEP Tris(2-chloroethyl) phosphate	+	284.9612	100.15	20	10.7	2.11
TBEP Tris (2-butoxyethyl) phosphate	+	399.2506	-	20	-	3.94
TPP Tripropyl phosphate	+	225.125	84.33	20	15	1.87
TDBPP Tris(2.3-dibromopropyl) phosphate	+	692.5881	120.04	20	17.2	3.65
TEHP Tris(2-ethylhexyl) phosphate	+	435.3598	113.02	20	22.8	6.07
TMPP Tricresyl phosphate	+	369.125	192.08	20	18.4	6.34
TPhP Triphenyl phosphate	+	327.0781	117.05	20	16.8	4.59
TDCIPP Tris(1.3-dichloro-2-propyl) phosphate	+	428.8912	80.13	20	16.7	3.8
TCIPP Tris(2-chloroisopropyl) phosphate	+	327.0081	80.01	20	14.9	1.44
CDP Cresyl diphenyl phosphate	+	341.0937	167.02	20	17.4	2.59
TnBP tributyl phosphate	+	267.172	-	20	-	4.51

Table S1.3. Pesticides list. CE: Collision energy; RT: Retention time.

Pesticides	Mode	Precursor mass (*m/z*)	Confirming fragment (*m/z*)	CE (V)	RT (min)	Log P
Acetamiprid	+	223.0745	223.0739	20	2.57	0.80
Alachlor	+	270.1255	69.1177	15	9.26	3.09
Atrazine	+	216.1011	174.0539	20	6.33	2.70
Atrazine-deethyl	+	188.0698	146.0226	20	3.46	1.51
Atrazine-Deisopropryl	+	174.0541	146.0229	20	2.63	1.15
Azinphos-ethyl	+	346.0444	132.0443	20	9.06	3.18
Azinphos-Methyl	+	318.0131	132.0442	20	7.18	2.96
Buprofezine	+	306.1635	201.1055	20	12.62	4.93
Carbendazim	+	192.0768	60.0505	20	2.21	1.48
Carbofuran	+	222.1125	165.0908	20	4.71	1.80
Carbofuran 3-OH	+	238.1074	163.0751	20	2.72	1.45
Chlorfenvinphos	+	358.9768	159.0419	20	10.51	3.80
Chlorpyriphos	+	349.9336	197.9270	20	13.58	4.00
Chlothianidin	+	250.0160	168.0463	20	2.66	0.70
Coumaphos	+	363.0217	306.9590	20	10.54	3.86
Diazinon	+	305.1083	153.1019	35	10.54	3.69
Dichlofenthion	+	313.9695	279.2323	5	13.20	5.14
Dimethoate	+	230.0069	198.9645	20	2.98	0.70
Diuron	+	233.0243	72.0444	35	6.94	2.87
DMA (2,4-dimethylaniline)	+	122.0976	107.0745	20	1.39	1.68
DMF (N-(2,4-dimethylphenyl formamide)	+	60.0570	-	20	-	-
DMPF N-(2.4-dimethylphenyl)-Nmethylformamidine	+	150.0913	123.0799	20	3.99	-
Ethion	+	384.9949	230.9737	20	13.23	5.07

Pesticides	Mode	Precursor mass (*m/z*)	Confirming fragment (*m/z*)	CE (V)	RT (min)	Log P
Etofenprox	+	394.2377	359.2004	20	15.13	7.05
Fenthion	+	279.0273	108.0443	10	-	4.84
Fenthion sulfone	+	311.0171	297.0011	20	5.37	2.25
Fenthion sulfoxide	+	295.0222	279.9984	20	4.55	1.92
Fipronil	+	436.9460	-	30	-	4.00
Hexythiazox	+	353.1085	228.0242	20	13.66	2.67
Imazalil	+	297.0556	255.0089	20	4.88	2.56
Imidacloprid	+	256.0596	209.0586	20	2.47	0.57
Isoproturon	+	207.4149	165.1020	20	6.28	2.50
Methiocarb	+	226.0896	170.1034	20	7.79	3.18
Methoalachlor	+	284.1412	253.1179	20	9.38	3.40
Molinate	+	188.1104	126.0912	20	8.25	2.86
Omethoate	+	214.0997	196.0188	20	1.40	-0.74
Parathion-ethyl	+	292.0403	123.1172	10	7.98	3.83
Prochloraz	+	376.0381	308.0002	20	10.62	3.50
Propanil	+	218.0134	161.9867	10	8.23	2.29
Propazine	+	230.1167	188.0696	20	7.63	3.95
Pyriproxyfen	+	322.1488	227.1066	20	13.10	5.37
Simazine	+	202.0854	146.0226	20	4.54	2.30
Spinosad A	+	732.4681	142.1223	20	9.80	4.10
Spinosad C	+	-	-	20	-	-
Spinosad D	+	746.4838	142.1225	20	10.73	-
Tebuconazole	+	308.1524	70.0397	20	10.30	3.70
Terbumeton	+	226.1662	170.1034	20	5.40	3.04
Terbumeton deethyl	+	198.1349	142.0723	20	3.50	-
Terbuthylazine	+	230.1167	174.0538	30	7.90	3.40
Terbuthylazine-2OH	+	212.1506	-	20	2.40	-
Terbuthylazine-deethyl	+	202.0854	146.0226	30	4.99	2.30
Terbutryn	+	242.1439	187.0833	20	8.15	3.66
Thiabendazole	+	202.0433	175.0337	20	2.55	2.39
Thiomethoxam	+	292.02656	211.0646	20	2.17	-0.13

Table S1.4. Perfluoroalkyl substances (PFAS) list. CE: Collision energy; RT: Retention time.

PFASs	Mode	Precursor mass (*m/z*)	Product ions (*m/z*)	CE (V)	RT (min)	Log P
Perfluorobutanoic acid (PFBA)	-	213	169	-12	3.18	--
Perfluoropentenoic acid (PFPeA)	-	263	219	-5	8.9	--
Perflurohexanoic acid (PFHxA)	-	313	269/119	-12/-10	6.74	3.48
Perfluroheptanoic acid (PFHpA)	-	363	319/169	-12/-10	7.94	4.15
Perfluorooctanoic acid (PFOA)	-	413	369/169	-14/-14	8.87	4.81
Perfluorononanoic acid (PFNA)	-	463	419/169	-14/-14	9.65	5.48

PFASs	Mode	Precursor mass (*m/z*)	Product ions (*m/z*)	CE (V)	RT (min)	Log P
Perfluoro-7-methyloctanoic acid (i.p-PFNA)	-	463	419/269	-14/-14	9.94	--
Perfluorodecanoic acid (PFDA)	-	513	469/269	-16/-16	10.31	--
Perfluoroundecanoic acid (PFUnDA)	-	563	519/269	-18/-18	10.88	--
Perfluorododecanoic acid (PFDoA)	-	613	569/269	-5/-10	11.38	--
Perfluorotridecanoic acid (PFTrDA)	-	663	619/169	-12/-24	11.88	--
Perfluorotetradecanoic acid (PFTeDA)	-	714	669/169	-12/-25	12.18	--
Perfluorohexadecanoic acid (PFHxDA)	-	814	769/169	-8/-28	12.79	--
Perfluorooctadecanoic acid (PFODA)	-	914	869/169	-8/-29	13.38	--
Perflurobutane sulfonate (PFBS)	-	299	99/80	-58/-58	5.54	--
Perfluorohexane sulfonate (PFHxS)	-	399	99/80	-78/-66	8.04	--
Perfluroheptane sulfonate (PFHpS)	-	449	99/80	-88/-88	8.89	--
Perfluoroctane sulfonate (PFOS)	-	499	99/80	-108/-108	9.63	4.49
Perfluoro-7-methyloctane sulfonate (ipPFNS)	-	427	99/80	-45/-78	10.10	--
Perfluorodecane sulfonate (PFDS)	-	599	99/80	-118/-118	10.83	--

Table S2.1. Quality parameters for quantification purposes of PhACs

PhACs	IS	*Procambarus Clarkii*		*Corbicula fluminea*		*Lepomis gibbosus*	
		R^2	Linearity range (ng g^{-1})	R^2	Linearity range (ng g^{-1})	R^2	Linearity range (ng g^{-1})
5-methyl-1H_Benzotriazole	Benzotriazole-D4	0.999	0.5-500	0.999	0.5-500	0.999	0.5-500
Acetaminophen	Acetaminophen-D4	0.990	10-500	0.997	2-500	0.999	2-500
Acridone	Carbamazepine-D10	0.994	0.1-500	0.996	0.2-200	0.999	0.1-200
Alprazolam	Alprazomal-D5	0.999	1-500	0.995	0.5-500	0.999	1-500
Amantadine	Trimethoprim-D9	0.997	1-500	0.998	0.2-500	0.996	0.2-500
Atenolol	Atenolol-D7	1.000	2-500	1.000	0.2-500	0.999	0.5-500
Benzoylecgonine	Sulfamethazine-D4	0.994	2-500	0.990	1-200	0.993	0.5-200
Benzotriazole	Benzotriazole-D4	0.998	1-500	0.999	0.5-500	0.996	1-500
Bezafibrate	Bezafibrate-D4	1.000	1-500	0.999	1-500	0.998	1-500
Bisphenol-A	Bisphenol-A-D8	0.999	0.2-500	0.998	0.5-500	0.998	0.5-500
Bromazepam	Bromazepam-D4	0.995	1-500	0.992	1-500	0.991	2-500
Caffeine	Caffeine-13C3	0.995	10-500	0.997	0.5-500	0.997	0.5-500
Carazolol	Carbamazepine-D10	0.990	0.5-500	0.998	0.2-500	0.999	1-500
Carbamazepine	Carbamazepine-D10	0.997	0.2-500	0.995	0.2-200	0.999	0.2-200
CBZ-10,11-epoxide	Carbamazepine-D10	0.999	0.1-500	0.998	0.1-200	0.994	0.2-200
Chloramphenicol	Ibuprofen-D3	0.992	1-500	0.994	0.5-200	0.997	0.5-500
Chlorpromazine	Chlorpromazine-D3	0.998	1-500	0.999	0.5-500	0.999	0.5-500
Ciprofloxacin	Lamotrigine-13CD3	0.990	2-500	0.993	0.1-500	0.997	0.5-500
Citalopram	Carbamazepine-D10	0.996	1-500	0.997	0.5-200	0.994	2-500
Clarithromycin	Carbamazepine-D10	0.993	2-500	0.996	0.5-200	0.992	2-500
Clofibric Acid	Furosemide-D5	0.997	2-500	0.992	1-200	0.999	1-500
Coca-ethylene	Cocaine-D5	0.994	0.5-500	0.997	0.1-500	0.992	0.2-500
Cocaine	Cocaine-D5	0.998	1-500	0.997	0.1-500	0.999	1-500
Codeine	Codeine-D3	0.998	2-500	0.999	0.2-500	0.999	0.1-500
Cotinine	Cotinine-D3	0.998	0.1-500	0.999	0.5-500	0.998	1-500
Diazepam	Bezafibrate-D4	0.998	0.1-500	0.999	0.2-500	0.998	0.5-500
Diclofenac	Diclofenac-13C6	0.999	1-500	0.999	2-500	1.000	10-500
Diltiazem	Carbamazepine-D10	0.996	0.5-500	0.998	0.2-500	0.995	0.5-500
Erythromycin	Erythromycin-13CD3	0.998	1-500	0.998	0.5-500	1.000	1-500
Fenofibrate	Fenofibrate-D6	0.995	10-500	0.998	10-500	0.999	10-500
Fipronil	Fipronil-13C3	0.995	1-500	0.999	1-200	0.992	2-200
Fipronil desulfynil	Fipronil-13C3	0.996	1-500	0.996	1-200	0.995	1-500
Fipronil sulfide	Fipronil-13C3	0.994	10-500	0.996	1-500	0.998	10-500
Fipronil sulfone	Fipronil-13C3	0.999	2-500	0.996	1-200	0.995	10-500
Fluconazole	Fluconazole-13C3	0.999	0.1-500	0.998	0.5-500	0.999	0.5-500
Flumequine	Carbamazepine-D10	0.995	0.2-500	0.995	0.1-500	0.994	0.2-500
Fluoxetine	Fluoxetine-D5	0.999	1-500	0.998	0.1-500	0.999	0.5-500

PhACs	IS	*Procambarus Clarkii*		*Corbicula fluminea*		*Lepomis gibbosus*	
		R^2	Linearity range (ng g^{-1})	R^2	Linearity range (ng g^{-1})	R^2	Linearity range (ng g^{-1})
Furazolidone	Carbamazepine-D10	0.998	1-500	0.996	0.5-100	0.992	0.5-500
Furosemide	Furosemide-D5	0.998	1-500	0.999	10-500	0.999	2-500
Hydrochlorothiazide	Hydrochlorothiazide-13CD2	0.999	0.1-500	1.000	0.2-500	0.997	0.2-500
Indomethacin	Indomethacin-D4	0.998	10-500	0.999	10-500	0.999	10-500
Ketoprofen	Ketoprofen-D3	0.997	2-500	0.999	10-500	0.999	10-500
Lamotrigine	Lamotrigine-13CD3	0.999	1-500	0.999	0.5-500	0.994	0.5-500
Loratadine	Carbamazepine-D10	0.997	1-500	0.990	0.5-500	0.994	1-500
Lorazepam	Lorazepam-D4	0.996	1-500	0.998	0.5-500	0.999	1-500
Losartan	Ibesartan-D6	0.998	1-500	0.973	10-500	0.998	10-500
Mefenamic acid	Ibuprofen-D3	0.998	1-500	0.996	1-500	0.998	1-500
Metformin	Metformin-D6	0.988	20-500	0.998	0.5-500	0.999	2-500
Methadone	Carbamazepine-D10	0.991	1-500	0.997	0.2-200	0.994	1-500
Metoprolol	Metoprolol-D7	0.999	1-500	0.999	1-500	0.999	0.2-500
Midazolam	Midazolam-13C6	0.998	0.5-500	0.996	0.5-500	0.999	0.2-500
Morphine	Morphine-D3	0.996	2-500	0.999	1-500	0.998	2-500
N-acethyl_SMX	Venlafaxine-D6	0.998	0.5-500	0.994	0.5-500	0.991	1-500
Nalidixin_acid	Venlafaxine-D6	0.997	0.2-500	0.998	0.1-200	0.995	0.2-500
N-Desmethylcitalopram	Carbamazepine-D10	0.994	0.5-500	0.992	0.5-500	0.990	1-500
Nicotine	Nicotine-D4	0.998	20-500	0.999	2-500	0.996	10-500
Norfluoxetine	Norfluoxetine-D6	0.997	10-500	1.000	1-500	1.000	10-500
O-desmethylvenlafaxine	Venlafaxine-D6	0.996	0.5-200	0.993	0.1-200	0.994	0.2-200
Omeprazole	Carbamazepine-D10	0.995	10-200	0.998	20-500	0.504	10-500
Oseltamivir	Oseltamivir-D3	0.998	2-500	0.999	0.2-500	0.998	1-500
Oseltamivir-CBX	Oseltamivir-D3	0.998	2-500	0.998	0.2-200	0.996	2-500
Oxazepam	Oxazepam-D5	0.999	0.1-500	0.999	0.5-500	0.997	0.5-500
Oxytetracycline	Trimethoprim-D9	0.997	10-500	0.993	10-500	0.987	10-500
Paroxetine	Paroxetine-D4	0.997	2-500	0.998	0.1-500	0.999	0.2-500
Pentobarbital	Pentobarbital-D5	0.992	1-500	0.998	0.5-500	1.000	1-500
Propyphenazone	Carbamazepine-D10	0.999	0.1-200	0.999	0.1-200	0.997	0.2-200
Quetiapine	Bromazepam-D4	0.996	0.5-500	0.999	0.2-500	0.999	0.5-500
Salbutamol	Salbutamol D6	0.993	0.1-200	0.990	0.5-500	0.997	0.2-500
Salicylic_acid	Sotalol-D6	0.996	0.1-500	0.997	2-500	0.996	0.1-500
Sertraline	Sertraline-D3	0.996	1-500	1.000	0.1-500	0.997	1-100
Sitagliptin	Sitagliptin-D4	0.998	2-500	1.000	0.5-500	1.000	1-500
Sotalol	Sotalol-D6	0.996	2-200	0.999	0.2-500	0.998	0.2-500
Sulfadimethoxine	Bromazepam-D4	0.997	0.1-500	0.995	0.2-500	0.996	0.2-500
Sulfamethazine	Sulfamethazine-D4	0.997	1-500	0.999	2-500	0.999	0.5-500
Sulfamethoxazole	Venlafaxine-D6	0.999	1-500	0.999	0.5-500	0.999	0.5-500

PhACs	IS	*Procambarus Clarkii* R²	*Procambarus Clarkii* Linearity range (ng g^{-1})	*Corbicula fluminea* R²	*Corbicula fluminea* Linearity range (ng g^{-1})	*Lepomis gibbosus* R²	*Lepomis gibbosus* Linearity range (ng g^{-1})
Sulfapyridine	Trimethoprim-D9	0.999	1-500	0.998	0.5-500	0.997	0.2-500
Temazepam	Oxazepam-D5	0.999	0.5-500	0.999	0.2-500	0.999	0.2-500
Tramadol	Tramadol-13CD3	0.997	0.5-500	0.999	0.1-500	0.999	0.1-500
Triclocarban	Fipronil-13C3	0.993	10-500	0.997	1-500	0.985	2-500
Trimethoprim	Trimethoprim-D9	0.996	1-500	0.998	0.1-500	0.998	0.2-500
Valsartan	Valsartan-D3	0.999	0.1-500	0.998	2-500	1.000	10-500
Valsartan acid	Valsartan acid D4	0.996	2-500	0.999	2-500	0.997	10-500
Venlafaxine	Venlafaxine-D6	0.998	0.1-500	0.999	0.1-500	0.999	0.1-500
Verapamil	Fluoxetine-D5	0.996	1-500	0.999	0.5-500	0.992	10-500
Warfarin	Pentobarbital-D5	0.998	1-500	0.991	0.5-200	0.992	0.2-200
Zolpidem	Venlafaxine-D6	0.996	0.5-500	0.994	0.5-500	0.996	1-500

Table S2.2. Quality parameters for quantification purposes of OPFRs

OPFRs	IS	*Procambarus Clarkii* R²	*Procambarus Clarkii* Linearity range (ng g^{-1})	*Corbicula fluminea* R²	*Corbicula fluminea* Linearity range (ng g^{-1})	*Lepomis gibbosus* R²	*Lepomis gibbosus* Linearity range (ng g^{-1})
TPP	D12-TCEP	n.d.	0.5-500	0.998	0.5-500	0.996	0.5-500
TDBPP	D15-TPhP	1,000	1-500	0.999	0.5-500	0.999	0.5-500
CDP	D15-TPhP	n.d.	20-500	0.998	20-500	0.997	20-500
TnBP	D15-TPhP	n.d.	0.2-500	0.999	0.2-500	0.998	0.2-500
TBEP	D15-TPhP	0.990	0.2-500	0.991	0.2-500	0.990	0.2-500
TMPP	D15-TPhP	0.990	0.5-500	0.995	0.5-500	0.992	0.2-500
TEHP	D15-TPhP	0.995	1-500	0.996	2-500	0.996	1-500
TCEP	D12-TCEP	0.998	0.2-500	0.995	0.2-500	0.990	0.2-500
TClPP	D18-TClPP	1,000	2-500	0.998	2-500	0.999	2-500
TDClPP	D15-TDClPP	0.998	2-500	0.999	1-500	0.997	1-500
TPhP	D15-TPhP	0.996	0.2-500	0.997	0.2-500	0.995	0.2-500

Table S2.3. Quality parameters for quantification purposes of Pesticides. The IS used were Chlorfenvinphos-d10 and Chlorpyrifos-d10. For the other analytes, quantification was performed with an external standard.

Pesticides	*Procambarus Clarkii* R²	*Procambarus Clarkii* Linearity range (ng g^{-1})	*Corbicula fluminea* R²	*Corbicula fluminea* Linearity range (ng g^{-1})	*Lepomis gibbosus* R²	*Lepomis gibbosus* Linearity range (ng g^{-1})
Acetamiprid	0.998	10-500	0.997	10-500	0.996	10-500
Alachlor	0.997	2-500	0.997	10-500	0.997	20-500

Pesticides	*Procambarus Clarkii*		*Corbicula fluminea*		*Lepomis gibbosus*	
	R^2	Linearity range (ng g^{-1})	R^2	Linearity range (ng g^{-1})	R^2	Linearity range (ng g^{-1})
Atrazine	0.998	10-500	0.998	10-500	0.999	10-500
Atrazine-desethyl	0.998	10-500	0.999	10-500	0.999	2-500
Atrazine-desisoproprуl	0.995	10-500	0.997	10-500	0.987	10-500
Azinphos-ethyl	0.997	20-500	0.998	2-500	0.999	20-500
Azinphos-methyl	0.994	10-500	0.996	20-500	0.997	10-500
Buprofezin	0.999	20-500	0.999	20-500	0.999	20-500
Carbendazim	0.997	2-500	0.997	10-500	0.996	1-500
Carbofuran	0.998	10-500	0.999	20-500	0.999	10-500
Carbofuran-3-hydroxy	0.998	10-500	0.998	10-500	0.999	10-500
Chlorfenvinphos	0.996	20-500	0.997	10-500	0.999	10-500
Chlorpyrifos	0.994	10-500	0.998	10-500	0.999	20-500
Chlothianidin	0.992	10-500	0.999	10-500	0.998	10-500
Coumaphos	0.997	20-500	0.998	10-500	0.999	2-500
Diazinon	0.996	20-500	0.998	10-500	0.997	20-500
Dichlofenthion	0.993	20-500	0.995	20-500	0.991	20-500
Dimethoate	0.997	10-500	0.998	20-500	0.998	2-500
Diuron	0.997	20-500	0.997	20-500	0.997	20-500
DMA	0.996	20-500	0.998	10-500	0.995	20-500
DMF	0.992	10-500	0.996	10-500	0.996	10-500
DMPF	0.997	10-500	0.998	10-500	0.996	10-500
Ethion	0.999	1-500	0.999	1-500	0.999	2-500
Etofenprox	0.994	20-500	0.997	20-500	0.999	20-500
Fenthion	0.992	20-500	0.996	10-500	0.997	20-500
Fenthion-sulfone	0.998	10-500	0.998	2-500	0.998	1-500
Fenthion sulfoxide	0.995	10-500	0.996	10-500	0.991	2-500
Fipronil	0.994	0.5-500	0.997	0.2-500	0.999	0.2-500
Hexythiazox	0.996	10-500	0.998	2-500	0.998	10-500
Imazalil	0.997	20-500	0.999	2-500	0.999	20-500
Imidacloprid	0.998	10-500	0.999	10-500	0.999	10-500
Isoproturon	0.997	10-500	0.998	2-500	0.997	2-500
Methiocarb	0.998	10-500	0.998	10-500	0.993	10-500
Metolachlor	0.998	10-500	0.999	10-500	0.998	2-500
Molinate	0.996	2-500	0.998	10-500	0.997	2-500
Omethoate	0.997	20-500	0.998	10-500	0.998	20-500
Parathion-ethyl	0.995	20-500	0.997	20-500	0.992	20-500
Prochloraz	0.997	20-500	0.999	10-500	0.999	10-500
Propanil	0.997	20-500	0.998	20-500	0.991	20-500
Propazine	0.997	10-500	0.998	2-500	0.996	2-500
Pyriproxyfen	0.996	20-500	0.997	20-500	0.999	20-500

Pesticides	*Procambarus Clarkii* R^2	*Procambarus Clarkii* Linearity range (ng g^{-1})	*Corbicula fluminea* R^2	*Corbicula fluminea* Linearity range (ng g^{-1})	*Lepomis gibbosus* R^2	*Lepomis gibbosus* Linearity range (ng g^{-1})
Simazine	0.997	10-500	0.998	10-500	0.997	2-500
Spinosyn A	0.995	10-500	0.996	10-500	0.994	10-500
Spinosyn C	0.994	10-500	0.994	10-500	0.994	2-500
Spinosyn D	0.995	10-500	0.997	2-500	0.995	10-500
Tebuconazole	0.997	10-500	0.998	10-500	0.997	10-500
Terbumeton	0.995	0.5-500	0.997	0.5-500	0.999	0.2-500
Terbumeton deethyl	0.996	0.5-500	0.994	0.5-500	0.999	0.2-500
Terbuthylazine	0.998	0.5-500	0.998	0.5-500	0.998	0.2-500
Terbuthylazine-2-hydroxy	0.997	2-500	0.998	2-500	0.998	2-500
Terbuthylazine-desethyl	0.998	2-500	0.999	10-500	0.999	2-500
Terbutryn	0.992	10-500	0.995	10-500	0.992	2-500
Thiabendazole	0.996	10-500	0.996	10-500	0.999	10-500
Thiamethoxam	0.997	10-500	0.998	10-500	0.999	10-500

Table S2.4. Quality parameters for quantification purposes of PFAS

PFAS	IS	*Procambarus Clarkii* R^2	*Procambarus Clarkii* Linearity range (ng g^{-1})	*Corbicula fluminea* R^2	*Corbicula fluminea* Linearity range (ng g^{-1})	*Lepomis gibbosus* R^2	*Lepomis gibbosus* Linearity range (ng g^{-1})
PFPeA	PFBA-D	0.990	10-500	0.991	10-500	0.992	10-500
PFBS	PFHxS-D	0.995	1-500	0.998	2-500	0.997	1-500
PFHpA	PFBA-D	0.996	0.5-500	0.993	2-500	0.998	2-500
PFHpS	PFHxS-D	0.991	0.2-500	0.995	1-500	0.998	0.5-500
IpPFNA	PFNA-D	0.992	10-200	0.990	10-500	0.998	10-500
FOUEA	PFDoDA-D	0.993	10-500	0.994	10-500	0.992	10-500
iPFNS	PFOS-D	0.995	0.5-500	0.998	20-500	0.990	0.5-500
PFDS	PFOS-D	0.990	10-500	0.990	10-500	0.994	10-500
PFTrDA	PFUnDA-D	0.992	0.2-500	0.997	0.2-500	1.000	0.2-500
PFTeDA	PFUnDA-D	0.993	20-500	0.990	20-500	0.990	20-500
PFHxDA	PFUnDA-D	0.990	0.5-500	0.990	20-500	0.994	20-500
PFODA	PFUnDA-D	0.998	10-500	0.999	10-500	0.992	10-500
PFBA	PFBA-D	0.991	10-500	0.996	10-500	0.995	10-500
PFHxA	PFHxA-D	0.995	1-500	0.995	10-500	0.996	10-500
PFHxS	PFHxS-D	0.991	0.1-500	0.990	0.1-500	0.997	0.1-500
PFOA	PFOA-D	0.990	0.2-500	0.992	0.2-500	0.997	0.2-500
PFOS	PFOS-D	0.993	1-500	0.997	10-500	0.992	2-500
PFNA	PFNA-D	0.997	10-500	0.998	10-500	0.995	10-500

**PFAS**	**IS**	***Procambarus Clarkii***		***Corbicula fluminea***		***Lepomis gibbosus***	
		**R^2**	**Linearity range (ng g^{-1})**	**R^2**	**Linearity range (ng g^{-1})**	**R^2**	**Linearity range (ng g^{-1})**
PFDA	PFDA-D	0.995	0.2-500	0.990	0.2-500	0.992	0.2-500
PFUnDA	PFUnDA-D	0.990	0.5-500	0.991	0.5-500	0.993	0.5-500
PFDoDA	PFDoDA-D	1.000	20-500	0.997	20-500	0.995	20-500

Table S3. Solvent and clean-up test results

		EXTRACTION SOLVENT TEST																							
		Procambarus clarkii												*Anodonta cygnea*											
	CECs	ACN + 0.1% FA			iPrOH/ACN (1:3) + 0.1% FA			H_2O:MeOH (7:3) + 1% F.A			MeOH			ACN + 0.1% FA			iPrOH/ACN (1:3) + 0.1% FA			H_2O:MeOH (7:3) + 1% F.A			MeOH		
		Recovery (%)	RSD (%)	Matrix effect (%)	Recovery (%)	RSD (%)	Matrix effect (%)	Recovery (%)	RSD (%)	Matrix effect (%)	Recovery (%)	RSD (%)	Matrix effect (%)	Recovery (%)	RSD (%)	Matrix effect (%)	Recovery (%)	RSD (%)	Matrix effect (%)	Recovery (%)	RSD (%)	Matrix effect (%)	Recovery (%)	RSD (%)	Matrix effect (%)
**OPFRs**	TPP	131	32	107	63	9	133	17	36	34	100	0.1	-97	131	19	-92	63	4	-89	61	17	110	45	11	-59
	TDBPP	79	80	139	135	2	119	135	23	-66	100	1	-97	25	18	-67	130	7	-8	22	27	140	35	34	-71
	CDP	<LOQ	-	-99	<LOQ	-	-93	<LOQ	-	-82	100	1	-56	<LOQ	-	-88	<LOQ	-	-91	<LOQ	-	-99	87	9	-27
	TnBP	136	16	96	90	4	118	68	31	-67	100	0.1	-97	128	21	-89	120	25	-92	77	6	140	95	11	-80
	TBEP	109	10	43	93	15	126	55	37	-94	100	0.01	-97	87	1	-46	126	31	-80	84	21	141	99	0.3	-97
	TMPP	88	33	67	91	17	65	10	2	-74	100	0.01	-97	68	15	-97	59	8	-96	50	43	-96	69	23	-75
	TEHP	107	16	126	97	29	106	22	39	65	100	0.1	-97	74	12	-70	37	29	-73	62	23	110	39	9	-85
	TCEP	100	1	141	97	4	129	100	0.1	167	82	21	-68	100	0	166	100	0	166	99	2	132	80	14	-41
	TClPP	100	14	53	92	10	47	95	1	-43	22	8	133	100	1	-47	100	0	-47	74	13	136	79	5	128
	TDClPP	67	27	-85	76	33	-87	103	1	-98	37	17	-54	100	0	-97	100	0	-97	58	33	-31	67	23	-51
	TPhP	99	1	46	98	0.4	45	99	0.1	35	136	46	-16	100	0	35	100	0.1	34	91	4	76	77	11	-55
**PFAS**	PFPeA	116	38	96	85	25	70	50	21	-30	73	12	-35	68	9	145	64	36	167	101	8	141	66	17	71
	PFBS	82	24	-62	109	36	-68	53	9	-63	25	31	-69	91	16	-64	41	36	-50	56	31	-50	<LOQ	-	-86
	PFHpA	89	12	-10	65	9	-7	43	12	-4	76	3	-9	79	17	30	53	29	29	74	24	7	78	2	45
	PFHpS	82	35	-27	78	16	-37	16	4	-44	146	32	-68	66	23	-40	21	21	-61	15	32	-34	86	12	-8
	ipPFNA	82	18	-35	67	13	-62	6	13	-42	141	23	-79	72	10	-25	39	36	-46	30	23	-25	70	4	6
	FOUEA	92	13	-15	80	33	-53	30	1	-18	132	6	-75	82	14	-13	70	2	-48	92	1	-73	73	4	41
	ipPFNS	31	6	-71	43	-	-93	<LOQ	-	-93	<LOQ	-	-84	<LOQ	-	-91	<LOQ	-	-95	<LOQ	-	-93	73	18	-42
	PFDS	40	11	-91	101	11	-97	24	6	-89	133	12	-96	69	40	-95	92	1	-97	73	3	-96	77	28	-52
	PFTrDA	72	10	-88	93	1	-92	48	1	-84	151	21	-95	80	2	-90	95	0.5	-92	73	2	-89	74	6	-50
	PFTeDA	<LOQ	-	-96	<LOQ	-	-83	<LOQ	-	-91	<LOQ	-	-84	<LOQ	-	-96	<LOQ	-	-92	<LOQ	-	-97	58	22	-71
	PFHxDA	62	1	-95	<LOQ	-	-98	<LOQ	-	-89	<LOQ	-	-96	<LOQ	-	-97	<LOQ	-	-97	<LOQ	-	-80	<LOQ	-	-87
	PFODA	88	1	-80	100	0.1	-82	78	0.01	-77	101	1	-82	99	4	-82	99	1	-82	99	0.2	-82	41	15	-36
	PFBA	94	13	44	78	19	41	63	8	77	100	9	58	63	16	146	64	34	126	82	15	159	65	13	158
	PFHxA	74	37	-17	75	7	16	130	29	-64	76	5	13	93	6	28	56	21	52	82	13	31	76	5	60
	PFHxS	113	9	-18	70	12	-22	36	23	-16	98	26	-42	89	15	-7	54	21	-13	62	29	-35	65	11	16
	PFOA	108	5	48	83	33	42	63	5	73	92	5	36	92	1	79	83	16	65	93	9	47	74	5	118
	PFOS	113	17	-54	45	38	-67	13	30	-69	<LOQ	-	-94	74	25	-66	31	15	-81	30	9	-75	51	18	-15
	PFNA	82	18	-44	62	20	-63	4	5	-40	<LOQ	-	-94	76	9	-23	47	22	-53	29	9	-35	53	31	-69
	PFDA	87	10	7	90	8	-18	67	2	3	121	9	-24	92	3	-4	93	5	-18	86	4	-14	89	2	52
	PFUnDA	40	4	-80	70	14	-95	16	12	-81	<LOQ	-	-98	65	15	-88	56	8	-95	29	35	-88	55	39	-61
	PFDoDA	<LOQ	-	-94	<LOQ	-	-96	<LOQ	-	-88	<LOQ	-	-96	91	4	-96	<LOQ	-	-96	<LOQ	-	-95	71	19	-59
**PESTICIDES**	Acetamiprid	52	31	141	131	31	110	<LOQ	-	-95	<LOQ	-	-90	76	33	-5	17	-	-10	45	33	-36	24	24	-20
	Alachlor	60	14	82	92	12	145	<LOQ	-	-84	3	-	-40	<LOQ	-	-93	<LOQ	-	-41	100	-	-80	29	13	114
	Atrazine	34	5	49	107	66	3	<LOQ	-	-80	<LOQ	-	-98	139	33	-86	<LOQ	-	-60	21	8	-34	11	35	-51
	Atrazine-Deethyl	70	5	38	129	6	25	<LOQ	-	-96	<LOQ	-	-84	56	9	-27	<LOQ	-	-40	40	29	-17	21	12	-24
	Atrazine-Deisoproryl	74	31	25	112	35	-0.4	<LOQ	-	-64	<LOQ	-	-78	63	13	-2	17	29	-7	128	37	-50	40	29	-29
	Azinphos-ethyl	<LOQ	-	-99	<LOQ	-	-83	<LOQ	-	-76	109	39	-72	<LOQ	-	-96	<LOQ	-	-91	<LOQ	-	-90	140	20	-80
	Azinphos-Methyl	26	34	114	105	7	141	<LOQ	-	-92	<LOQ	-	-96	<LOQ	-	-85	<LOQ	-	-90	<LOQ	-	-99	<LOQ	-	-82
	Buprofezine	<LOQ	-	-93	<LOQ	-	-81	<LOQ	-	-93	<LOQ	-	-83	<LOQ	-	-81	<LOQ	-	-89	<LOQ	-	-91	<LOQ	-	-96
	Carbendazim	54	7	74	118	7	28	<LOQ	-	-77	<LOQ	-	-99	61	25	-7	20	-	-23	50	13	7	41	15	1
	Carbofuran	11	-	60	72	14	67	<LOQ	-	-69	<LOQ	-	-95	44	12	-50	<LOQ	-	-60	21	17	-67	<LOQ	-	-67
	Carbofuran 3-OH	34	36	141	106	6	140	82	19	-92	118	-	171	75	30	30	33	40	28	48	9	-13	52	28	117
	Chlorfenvinphos	<LOQ	-	198	69	-	141	<LOQ	-	-79	139	6	-80	47	-	-90	<LOQ	-	-80	64	-	-28	140	13	-64
	Chlorpyriphos	66	7	5	37	28	-58	49	14	-22	99	29	-41	137	24	135	55	5	140	<LOQ	-	115	68	22	34
	Chlothianidin	60	37	126	79	14	96	56	12	-16	80	7	-30	72	9	94	48	26	64	112	6	-10	62	7	63
	Coumaphos	<LOQ	-	-16	141	17	-69	<LOQ	-	-99	86	2	-97	<LOQ	-	-84	<LOQ	-	-99	<LOQ	-	-91	57	7	-90
	Diazinon	<LOQ	-	-97	<LOQ	-	-80	<LOQ	-	-80	169	16	-84	<LOQ	-	-89	<LOQ	-	-84	<LOQ	-	-82	73	13	-57
	Dichlofenthion	<LOQ	-	-90	<LOQ	-	-99	<LOQ	-	-84	<LOQ	-	-90	<LOQ	-	-92	<LOQ	-	-78	<LOQ	-	-86	<LOQ	-	-84
	Dimethoate	59	9	-12	143	17	-41	<LOQ	-	-82	<LOQ	-	-84	94	23	-73	<LOQ	-	-63	22	39	-61	<LOQ	-	-85
	Diuron	<LOQ	-	-80	<LOQ	-	-84	<LOQ	-	-95	<LOQ	-	-86	<LOQ	-	-90	<LOQ	-	-90	<LOQ	-	-96	<LOQ	-	-82
	DMA	<LOQ	-	-93	<LOQ	-	-90	<LOQ	-	-93	100	2	-97	<LOQ	-	-99	<LOQ	-	-82	<LOQ	-	-99	119	35	-94
	DMF	77	9	-65	29	12	-71	<LOQ	-	-97	93	5	-97	46	22	-55	<LOQ	-	-51	20	20	-59	141	5	-46
	DMPF	66	6	74	105	8	33	<LOQ	-	-91	<LOQ	-	-93	113	18	62	47	25	17	58	3	8	135	36	-3
	Ethion	120	18	-28	89	6	19	22	23	-12	138	28	-93	41	34	-38	57	13	19	108	38	23	125	31	-86
	Etofenprox	<LOQ	-	-98	<LOQ	-	-99	<LOQ	-	-93	<LOQ	-	-79	<LOQ	-	-90	<LOQ	-	-79	<LOQ	-	-80	<LOQ	-	-84
	Fenthion	<LOQ	-	-15	141	14	-97	<LOQ	-	-90	101	20	-85	<LOQ	-	-89	<LOQ	-	-86	<LOQ	-	-97	45	28	-53
	Fenthion sulfone	46	37	-67	97	7	141	<LOQ	-	-89	53	9	-4	59	28	-49	<LOQ	-	-21	<LOQ	-	-90	68	17	15
	Fenthion sulfoxide	65	36	168	120	7	135	<LOQ	-	-98	<LOQ	-	-77	70	31	132	56	14	116	33	13	-35	39	22	9
	Fipronil	30	25	-87	133	9	140	63	25	-31	47	21	-75	31	22	-98	10	30	-68	45	30	3	140	2	-92
	Hexythiazox	49	19	-93	47	14	-90	141	10	-97	93	5	-95	58	20	-95	80	23	-97	151	3	-96	117	39	-97
	Imazalil	<LOQ	-	-96	<LOQ	-	-84	<LOQ	-	-99	34	0	-93	<LOQ	-	-87	<LOQ	-	-93	<LOQ	-	-99	99	0	-97
	Imidacloprid	54	41	48	76	10	140	67	5	3	85	14	13	86	10	139	53	14	105	102	11	31	73	10	86
	Isoproturon	54	32	38	117	7	141	<LOQ	-	-84	59	19	-47	55	9	-8	<LOQ	-	-8	33	35	-28	76	19	57
	Methiocarb	45	14	192	56	18	192	137	5	3	58	3	17	77	14	43	61	13	41	94	7	44	78	2	54
	Methoalachlor	36	15	169	100	10	32	<LOQ	-	-81	11	-	-66	117	26	-73	<LOQ	-	-13	153	-	-42	24	38	-40
	Molinate	100	17	-53	156	17	-75	<LOQ	-	-80	143	17	19	<LOQ	-	-95	139	-	-96	<LOQ	-	-76	61	4	134
	Omethoate	<LOQ	-	-92	<LOQ	-	-91	<LOQ	-	-84	98	16	-94	<LOQ	-	-96	<LOQ	-	-95	<LOQ	-	-92	62	7	-65
	Parathion-ethyl	<LOQ	-	-80	<LOQ	-	-95	<LOQ	-	-92	<LOQ	-	-84	<LOQ	-	-99	<LOQ	-	-89	<LOQ	-	-93	<LOQ	-	-72
	Prochloraz	<LOQ	-	-3	117	-	-79	<LOQ	-	-87	99	4	-97	<LOQ	-	-84	<LOQ	-	-90	<LOQ	-	-84	108	34	-97
	Propanil	<LOQ	-	-98	<LOQ	-	-86	<LOQ	-	-83	<LOQ	-	-92	<LOQ	-	-89	<LOQ	-	-81	<LOQ	-	-80	<LOQ	-	-89
	Propazine	56	41	68	113	20	12	46	39	-45	80	6	-30	105	11	-44	66	20	-16	62	7	52	71	20	14
	Pyriproxyfen	<LOQ	-	-84	<LOQ	-	-72	<LOQ	-	-95	<LOQ	-	-91	<LOQ	-	-93	<LOQ	-	-91	<LOQ	-	-82	<LOQ	-	-99
	Simazine	67	41	-33	106	9	-24	<LOQ	-	-97	<LOQ	-	-79	78	8	-75	<LOQ	-	-80	57	37	-70	48	7	-46
	Spinosad A	13	44	138	56	18	141	46	37	-61	19	1	-87	133	15	-95	41	18	-82	49	34	-84	24	5	-53
	Spinosad C	32	14	-98	95	15	129	105	17	137	113	1	-96	72	14	122	37	-	109	96	16	122	12	22	-74
	Spinosad D	74	38	133	58	12	137	132	12	-29	101	1	-97	<LOQ	-	-65	40	40	-38	64	9	35	100	1	-97
	Tebuconazole	26	6	-84	50	10	131	57	39	-39	48	11	2	80	5	-34	34	5	-13	72	13	37	63	10	45
	Terbumeton	46	35	-82	86	9	127	70	6	-30	59	4	27	106	38	-28	33	15	-5	73	9	78	63	23	30
	Terbumeton deethyl	69	13	141	84	19	134	52	6	53	88	2	-5	85	19	122	50	22	100	106	9	111	63	2	146
	Terbuthylazine	50	25	9	114	14	-35	34	20	-64	72	16	-51	97	9	-68	14	35	-56	53	29	29	73	36	-8
	Terbuthylazine-2OH Results	91	14	73	98	10	75	79	39	140	104	0.1	-38	96	20	118	75	11	120	<LOQ	-	-82	104	0.3	-36
	Terbuthylazine-deethyl	90	5	39	91	6	39	64	12	160	104	9	62	99	6	127	66	22	102	89	10	134	70	6	120
	Terbutryn	41	6	24	118	10	-30	58	24	-68	53	24	-46	124	17	-83	55	25	-77	59	10	20	70	36	-20
	Thiabendazole	66	15	136	73	17	140	60	6	61	85	1	17	58	4	90	59	12	94	99	4	82	71	1	125
	Thiametosan	54	31	-47	85	18	-25	74	17	-37	74	5	-24	78	22	125	45	15	85	114	7	-10	72	18	59
**PhACs**	5-methyl-1 H Benzotriazole	86	17	-6	55	5	-3	67	13	-21	73	4	-22	84	3	-7	67	15	-15	68	11	-5	65	3	-15
	Acetaminophen	32	19	-34	17	-	-60	0	-	-	10	3	-57	84	8	-2	60	13	-31	74	15	-46	73	4	-24
	Acridone	92	10	-10	58	3	-21	55	11	-27	68	1	-28	80	7	3	66	22	-17	57	15	-19	65	3	-18
	Alprazolam	94	9	55	59	0.2	45	70	7	37	67	2	52	74	7	18	65	20	19	65	10	57	71	1	38
	Amantadine	81	13	-35	57	5	-38	79	12	-60	56	11	-56	57	20	-28	63	15	-5	68	15	-34	60	2	-7
	Atenolol	54	11	-30	61	22	-53	68	9	-56	60	15	-58	46	8	45	60	18	49	70	9	4	66	2	29
	Atorvastatin	83	10	-16	60	7	-38	19	22	-26	141	2	-67	14	-	-76	0	-	-	45	3	-48	8	21	-66
	Benzotriazole	62	19	35	55	0.3	-4	42	8	8	39	5	18	78	4	4	61	21	1	50	11	12	33	2	68
	Benzoylecgonine	66	9	53	60	2	17	50	9	11	96	1	-15	52	6	32	58	18	61	69	13	10	70	2	53
	Bezafibrate	71	13	41	56	9	47	41	12	20	82	1	4	53	4	61	63	24	38	51	18	20	67	3	31
	Bisphenol-A	86	10	-24	47	11	-36	55	7	-36	62	2	-33	80	4	-20	73	12	-25	57	12	-3	67	1	-4

		CLEAN-UP STEP TEST																	
		Procambarus clarkii									*Anodonta cygnea*								
	CECs	Z-Sep			Z-Sep+			Z-Sep/C18			Z-Sep			Z-Sep+			Z-Sep/C18		
		Recovery (%)	RSD (%)	Matrix effect (%)	Recovery (%)	RSD (%)	Matrix effect (%)	Recovery (%)	RSD (%)	Matrix effect (%)	Recovery (%)	RSD (%)	Matrix effect (%)	Recovery (%)	RSD (%)	Matrix effect (%)	Recovery (%)	RSD (%)	Matrix effect (%)
**OPFRs**	TPP	77	12	-65	98	0.3	-97	75	15	-72	100	0.1	-97	100	0.1	-97	63	14	-83
	TDBPP	104	1	-98	100	0.2	-97	6	18	-91	100	0.3	-97	100	0.6	-97	114	22	-71
	CDP	95	1	-52	100	0.5	-55	<LOQ	-	-93	100	0.8	-55	100	0.5	-55	<LOQ	-	-82
	TnBP	107	22	-88	99	0.2	-97	90	22	-85	100	0.1	-97	100	0.1	-97	95	9	-91
	TBEP	99	0.1	-97	100	0.01	-97	117	1	-96	100	0.02	-97	100	0.01	-97	36	17	-31
	TMPP	100	1	-97	100	0.1	-97	128	24	-97	100	0.1	-97	100	0.04	-97	110	25	-98
	TEHP	99	0.1	-97	100	0.2	-97	127	34	-54	100	0.03	-97	100	0.01	-97	139	34	-85
	TCEP	78	4	-39	53	22	-1	100	0.4	166	125	20	-15	52	21	-47	100	0.02	-80
	TClPP	64	35	1	49	18	-36	99	2	-46	32	26	-71	<LOQ	-	-99	100	0.3	-47
	TDClPP	55	21	-93	17	5	2	100	0.5	-97	17	0.7	-85	111	9	-83	100	0.5	-97
	TPhP	70	13	-86	12	-	-48	100	0.1	34	125	34	240	124	13	29	100	0.2	34
**PFAS**	PFPeA	18	31	141	98	2	-68	87	27	99	68	35	140	25	26	136	78	5	-1
	PFBS	<LOQ	-	-68	<LOQ	-	-92	75	24	-58	9	9	-67	<LOQ	-	-71	50	26	-62
	PFHpA	57	31	-26	71	24	-55	101	22	-3	72	9	12	36	13	20	58	4	12
	PFHpS	89	-	-10	62	12	-24	83	11	3	85	25	-64	74	27	-61	48	15	-22
	ipPFNA	81	25	-26	43	8	-15	91	13	-22	64	5	-19	11	24	-20	46	5	-4
	FOUEA	66	16	-17	43	3	-32	85	11	-10	73	4	-28	52	8	-44	69	5	-23
	ipPFNS	75	20	-51	64	53	-62	117	31	-69	106	22	-82	11	23	-72	39	20	-71
	PFDS	88	27	-72	<LOQ	-	-89	102	20	-96	67	28	-81	37	15	-92	28	22	-88
	PFTrDA	57	30	-74	70	16	-92	117	9	-89	100	3	-87	79	5	-94	81	21	-86
	PFTeDA	124	-	-91	<LOQ	-	-96	150	9	-97	<LOQ	-	-94	<LOQ	-	-96	96	10	-96
	PFHxDA	<LOQ	-	-96	<LOQ	-	-94	162	15	-96	<LOQ	-	-94	<LOQ	-	-88	<LOQ	-	-93
	PFODA	65	34	-66	93	1	-81	105	11	-82	81	15	-74	100	0	-82	102	12	-81
	PFBA	38	21	92	13	15	-37	61	13	108	60	9	123	62	19	80	76	11	107
	PFHxA	28	7	7	101	7	-68	125	15	-65	79	12	26	54	26	15	69	5	22
	PFHxS	80	17	-17	74	15	-2	76	13	19	66	12	-14	54	17	-17	59	3	-11
	PFOA	107	11	61	84	4	70	103	6	81	98	4	62	61	3	63	87	8	61
	PFOS	65	19	-71	<LOQ	-	-72	123	28	-72	61	20	-69	<LOQ	-	-56	62	15	-53
	PFNA	174	31	-82	61	-	-82	90	13	-32	<LOQ	-	-81	<LOQ	-	-86	46	5	-16
	PFDA	96	17	13	76	2	25	106	4	14	103	5	5	68	1	19	90	2	11
	PFUnDA	167	-	-89	<LOQ	-	-81	115	22	-78	<LOQ	-	-93	<LOQ	-	-83	49	22	-74
	PFDoDA	100	-	-81	<LOQ	-	-84	118	8	-93	22	10	-85	<LOQ	-	-96	32	29	-87
**PESTICIDES**	Acetamiprid	67	9	124	110	7	80	49	15	24	29	16	-10	23	7	-11	30	27	-47
	Alachlor	122	39	-52	<LOQ	-	-24	61	9	23	65	-	-20	117	14	-80	97	31	-1
	Atrazine	81	33	-20	80	7	42	71	15	-24	<LOQ	-	-63	14	6	-28	61	17	5
	Atrazine-Deethyl	51	28	63	54	29	-21	26	34	-25	24	15	-37	24	13	-29	32	28	-33
	Atrazine-Deisoproryl	54	19	43	36	22	-37	72	4	36	41	3	-37	44	25	-21	72	15	49
	Azinphos-ethyl	140	11	-76	106	21	-77	85	30	81	34	4	-61	45	17	-39	157	11	62
	Azinphos-Methyl	50	32	-19	40	23	4	69	28	-29	<LOQ	-	-82	<LOQ	-	-98	61	7	-18
	Buprofezine	<LOQ	-	-92	<LOQ	-	-93	84	15	-92	<LOQ	-	-93	<LOQ	-	-90	59	30	-81
	Carbendazim	52	4	121	56	29	42	56	25	-1	51	21	-11	38	6	17	50	13	-1
	Carbofuran	12	-	74	<LOQ	-	-15	15	22	-2	27	14	-68	<LOQ	-	-68	68	15	-26
	Carbofuran 3-OH	<LOQ	-	111	49	21	124	58	13	43	36	-	-11	96	-	-80	73	17	27
	Chlorfenvinphos	48	31	-53	7	39	115	81	27	-90	113	25	-66	133	36	-55	99	15	-91
	Chlorpyriphos	55	33	-63	41	25	-87	<LOQ	-	-91	51	34	61	93	15	38	<LOQ	-	-95
	Chlothianidin	65	19	127	92	38	119	72	7	140	65	9	81	69	10	94	68	9	74
	Coumaphos	88	34	-89	44	33	-68	113	8	-92	45	2	-94	65	28	-67	55	28	-61
	Diazinon	60	30	-31	41	30	-13	102	10	-91	68	30	-65	80	36	-58	59	29	-78
	Dichlofenthion	<LOQ	-	-86	<LOQ	-	-84	54	17	-90	<LOQ	-	-81	<LOQ	-	-92	50	37	-89
	Dimethoate	48	13	-18	64	4	-75	58	11	36	<LOQ	-	-84	<LOQ	-	-83	61	5	-6
	Diuron	<LOQ	-	-87	<LOQ	-	-94	78	16	-22	<LOQ	-	-99	<LOQ	-	-93	85	35	95
	DMA	100	12	-97	99	1	-97	<LOQ	-	-91	80	1	-97	143	37	-97	<LOQ	-	-91
	DMF	65	29	-9	4	10	-30	62	13	-20	25	22	-84	22	12	-77	68	13	-28
	DMPF	38	18	136	60	11	24	50	3	19	70	18	7	45	9	15	51	11	31
	Ethion	128	12	-94	63	13	-95	<LOQ	-	-80	83	35	-88	121	12	-92	<LOQ	-	-74
	Etofenprox	<LOQ	-	-80	<LOQ	-	-81	73	33	-95	<LOQ	-	-99	<LOQ	-	-84	45	16	-91
	Fenthion	147	3	-94	53	24	-62	135	31	-81	131	8	-95	105	20	-91	74	10	-80
	Fenthion sulfone	64	26	136	63	29	140	72	21	70	65	14	18	71	26	16	74	17	14
	Fenthion sulfoxide	94	25	140	78	19	141	52	19	-13	50	27	107	20	-	5	48	17	-42
	Fipronil	50	32	-31	52	38	-39	97	29	161	61	13	-86	29	18	-56	52	9	114
	Hexythiazox	66	15	-96	57	8	-95	56	24	-92	128	4	-97	95	6	-97	86	30	-88
	Imazalil	99	1	-97	36	0	-93	73	13	-88	100	0.1	-97	100	-	-97	105	26	-79
	Imidacloprid	68	23	145	79	14	143	71	13	154	74	10	111	63	8	124	72	6	80
	Isoproturon	97	11	126	74	17	141	69	12	54	108	10	32	33	33	63	66	4	23
	Methiocarb	49	23	107	42	15	137	51	7	46	103	9	27	74	5	78	86	8	43
	Methoalachlor	35	8	179	50	18	125	60	28	-26	16	8	-72	22	22	-7	72	20	3
	Molinate	88	26	114	47	6	138	27	20	15	107	8	198	110	25	-4	58	-	-58
	Omethoate	61	17	-37	68	22	-62	52	7	-43	59	18	-61	47	13	-60	68	22	-63
	Parathion-ethyl	<LOQ	-	-99	<LOQ	-	-84	97	24	-75	<LOQ	-	-92	<LOQ	-	-84	61	43	-47
	Prochloraz	100	1	-97	67	1	-96	73	15	-87	136	6	-97	60	2	-96	79	34	-67
	Propanil	<LOQ	-	-87	<LOQ	-	-98	78	20	-40	<LOQ	-	-80	<LOQ	-	-89	78	13	-33
	Propazine	89	28	-7	77	14	141	75	30	-69	68	6	-9	56	19	74	64	9	-18
	Pyriproxyfen	<LOQ	-	-98	<LOQ	-	-82	102	17	-93	<LOQ	-	-83	<LOQ	-	-75	74	36	-84
	Simazine	60	24	-9	60	21	-34	36	9	-63	31	24	-52	24	36	-39	47	24	-67
	Spinosad A	23	1	-89	9	2	19	<LOQ	-	-85	20	1	-83	56	19	-89	<LOQ	-	-85
	Spinosad C	40	1	-91	43	19	-97	<LOQ	-	-91	97	30	-95	34	1	-97	<LOQ	-	-96
	Spinosad D	100	0	-97	96	1	-97	<LOQ	-	-72	100	0.1	-97	89	0.2	-97	<LOQ	-	-99
	Tebuconazole	35	4	53	10	31	140	115	30	-76	90	22	-58	32	4	53	58	37	-14
	Terbumeton	75	10	48	55	13	137	105	15	-64	70	20	-12	58	7	89	60	16	-3
	Terbumeton deethyl	78	20	137	70	12	99	73	10	109	75	4	138	57	7	104	76	13	108
	Terbuthylazine	89	20	-47	73	10	139	105	29	-79	65	21	-34	57	5	82	55	16	-28
	Terbuthylazine-2OH Results	100	0	-35	100	4	-35	<LOQ	-	-90	100	0.3	-35	100	0.2	-35	<LOQ	-	-84
	Terbuthylazine-deethyl	92	28	125	79	35	123	98	6	200	94	16	166	73	11	125	109	3	181
	Terbutryn	61	21	-52	48	17	135	49	11	-96	70	33	-79	62	14	42	26	20	-54
	Thiabendazole	65	27	46	50	39	113	72	9	18	121	15	-23	60	6	116	81	4	81
	Thiametosan	68	10	107	76	6	124	66	10	127	74	5	85	58	8	98	72	14	44
**PhACs**	5-methyl-1 H Benzotriazole	73	12	-30	74	37	-22	80	4	-30	64	3	-9	57	8	13	57	2	14
	Acetaminophen	33	52	-62	13	19	-62	15	2	-66	64	4	-38	53	7	-14	58	1	-35
	Acridone	79	10	-43	89	3	-41	87	3	-44	64	3	-28	51	4	-6	58	2	-13
	Alprazolam	70	10	19	48	11	28	71	3	18	71	4	1	37	5	10	53	3	48
	Amantadine	78	67	-49	29	4	-22	57	12	-54	56	3	-15	30	3	37	52	6	30
	Atenolol	35	2	-56	13	10	-47	42	4	-68	53	12	-1	36	3	17	59	2	18
	Atorvastatin	5	22	-0.2	1	9	7	20	6	16	0	-	-77	0	-	-27	0	-	-73
	Benzotriazole	76	8	-13	90	27	-13	83	7	-14	68	3	13	61	3	33	61	3	35
	Benzoylecgonine	22	16	-12	8	11	-14	42	7	-11	40	13	-5	16	4	10	57	3	18
	Bezafibrate	17	6	34	7	20	29	35	10	44	31	20	31	15	5	14	49	3	62
	Bisphenol-A	79	17	-6	67	5	3	100	9	-3	80	7	-17	46	6	7	63	9	9

	CECs	EXTRACTION SOLVENT TEST – *Procambarus clarkii* – ACN + 0.1% FA Recovery (%)	ACN + 0.1% FA RSD (%)	ACN + 0.1% FA Matrix effect (%)	iPrOH/ACN (1:3) + 0.1% FA Recovery (%)	iPrOH/ACN (1:3) + 0.1% FA RSD (%)	iPrOH/ACN (1:3) + 0.1% FA Matrix effect (%)	H_2O:MeOH (7:3) + 1% F.A Recovery (%)	H_2O:MeOH (7:3) + 1% F.A RSD (%)	H_2O:MeOH (7:3) + 1% F.A Matrix effect (%)	MeOH Recovery (%)	MeOH RSD (%)	MeOH Matrix effect (%)
PhACs	Bromazepam	85	19	43	61	7	140	39	2	207	93	4	158
	Caffeine	101	10	129	70	21	110	73	1	70	111	4	30
	Carazolol	133	14	-45	71	8	-48	90	10	-33	41	7	-12
	Carbamazepine	92	9	7	56	7	-10	66	10	-25	67	1	-16
	Carbamazepine-10,11-epoxide	100	4	22	57	5	58	59	14	35	79	1	16
	Chloramphenicol	93	7	-11	62	4	-6	69	10	-9	61	3	-1
	Chlorpromazine	176	19	-65	137	21	-92	54	26	-28	39	2	-22
	Ciprofloxacin	3	22	12	31	19	12	40	13	-22	23	7	-26
	Citalopram	136	13	-57	70	9	-59	97	14	-46	43	5	-20
	Clarithromycin	104	12	-47	62	3	-47	107	9	-48	48	2	-21
	Clofibric acid	56	9	7	60	2	24	46	4	39	69	5	36
	Cocaethylene	103	10	-34	64	1	-34	87	14	-46	55	3	-33
	Cocaine	98	7	-25	59	5	-34	91	14	-57	62	3	-45
	Codeine	77	9	37	54	5	4	60	2	-32	79	6	-38
	Cotinine	69	22	11	69	26	-26	75	11	-42	76	2	-49
	Diazepam	94	10	-41	61	7	-54	58	10	-42	60	3	-34
	Diclofenac	87	2	61	103	11	38	89	2	33	83	3	30
	Diltiazem	129	13	-51	71	8	-49	98	12	-41	49	6	-16
	Erythromycin	81	5	-18	61	0.4	-36	12	23	-51	765	7	-95
	Fenofibrate	159	28	-77	234	13	-94	2	12	-25	361	5	-93
	Fipronil Desulfynil	136	25	-49	95	4	-85	14	70	-15	164	2	-69
	Fipronil Sulfide	136	25	-49	95	4	-85	19	24	-15	164	2	-69
	Fipronil Sulfone	149	20	-46	105	6	-86	13	27	-2	197	8	-72
	Fipronil	99	16	-27	74	7	-62	33	38	-14	90	3	-34
	Fluconazole	89	10	12	62	4	22	89	13	20	64	3	36
	Flumequine	17	7	30	51	11	17	64	11	5	31	10	23
	Fluoxetine	175	20	-66	136	25	-92	80	22	-33	34	3	-6
	Furazolidone	89	12	17	55	8	2	81	9	-62	30	7	-27
	Furosemide	79	11	-1	57	6	-16	37	8	1	70	2	-9
	Hydrochlorothizade	84	24	-2	63	11	-13	48	7	-24	75	2	-37
	Indomethacin	68	10	-17	63	6	-49	8	14	-34	146	5	-73
	Irgasan	155	70	-53	183	48	-80	0	-	9	180	78	-91
	Ketoprofen	65	12	51	59	16	45	47	12	27	76	3	19
	Lamotrigine	90	13	-25	60	4	-61	58	11	-57	77	5	-61
	Loratadine	140	16	-63	161	17	-92	31	69	-25	80	4	-50
	Lorazepam	104	9	6	61	5	14	42	14	37	57	18	7
	Losartan potassium	71	13	19	56	5	-10	40	13	-26	106	3	-45
	Mefenamic acid	75	7	-9	69	3	-70	0	-	-16	389	2	-87
	Metformin	20	27	-95	0	-	-	0	-	-	0	-	-
	Methadone	155	16	-69	77	6	-79	102	9	-45	38	7	-17
	Metoprolol	93	5	0	61	1	12	74	10	-11	66	4	-5
	Midazolam	96	21	-39	69	7	-68	77	9	-40	58	5	-27
	Morphine	62	12	-28	55	14	-63	61	8	-74	65	4	-74
	N-acethyl SMX	60	8	8	61	5	29	58	16	11	72	2	9
	Nalidixic acid	9	14	6	51	9	-11	66	12	-23	28	11	-12
	N-Desmethylcitalopram	140	13	-60	72	8	-67	107	14	-50	37	8	-19
	Norfluoxetine	186	18	-62	162	29	-91	66	30	2	33	3	28
	O-desmethylvenlafaxine	80	6	-11	58	3	-35	71	11	-55	71	6	-56
	Omeprazole	98	7	36	95	6	-68	0	-	-	0	1	-100
	Oseltamivir phosphate	102	16	-27	66	9	96	84	9	139	58	3	187
	Oseltamivir-CBX	6	20	6	60	4	40	60	7	40	79	3	27
	Oxazepam	98	9	-5	69	0.5	28	46	10	51	63	3	38
	Oxytetracycline	0	-	-	18	64	32	65	21	19	0	-	-
	Paroxetine	148	19	-50	131	27	-90	75	17	-9	26	4	34
	Pentobarbital	102	4	-15	66	2	-18	70	8	-21	76	2	-20
	Propyphenazone	93	9	-8	54	4	-24	74	11	-32	66	2	-23
	Salbutamol	56	9	57	56	11	2	71	6	-29	65	5	-27
	Salicylic acid	71	5	-6	59	3	-13	175	11	-13	35	5	57
	Sertraline	142	18	-37	135	31	-88	43	39	7	51	7	-9
	Sitagliptin	104	8	-25	63	4	27	67	4	75	63	1	83
	Sotalol	72	8	-17	59	5	-35	82	7	-55	61	6	-49
	Sulfadimethoxine	84	7	20	63	4	83	30	5	119	129	6	9
	Sulfamethazine	95	0	36	67	6	50	21	9	101	140	3	-8
	Sulfamethoxazole	80	8	15	60	6	27	29	13	36	122	4	-30
	Sulfapyridine	91	1	12	63	7	29	18	8	75	143	5	-26
	Temazepam	101	8	92	60	3	81	58	9	84	63	2	90
	Tramadol	92	6	-19	60	6	-32	72	11	-50	68	3	-50
	Triclocarban	190	31	183	151	32	-65	0	-	-	4789	2	-96
	Trimethoprim	88	8	-15	60	0.3	-30	64	6	-43	74	1	-46
	Valsartan	29	14	93	49	11	85	26	15	49	131	4	-16
	Valsartan acid	5	33	-15	37	0.2	-28	38	6	-41	65	14	-48
	Venlafaxine	97	9	-35	60	0.5	-37	83	13	-51	59	4	-42
	Verapamil	138	16	-52	77	14	-62	94	8	-21	47	3	8
	Warfarin	86	10	147	130	9	41	51	6	164	60	2	172
	Zolpidem	95	11	-13	61	0.4	-30	74	11	-35	70	2	-31

CECs	EXTRACTION SOLVENT TEST – *Anodonta cygnea* – ACN + 0.1% FA Recovery (%)	ACN + 0.1% FA RSD (%)	ACN + 0.1% FA Matrix effect (%)	iPrOH/ACN (1:3) + 0.1% FA Recovery (%)	iPrOH/ACN (1:3) + 0.1% FA RSD (%)	iPrOH/ACN (1:3) + 0.1% FA Matrix effect (%)	H_2O:MeOH (7:3) + 1% F.A Recovery (%)	H_2O:MeOH (7:3) + 1% F.A RSD (%)	H_2O:MeOH (7:3) + 1% F.A Matrix effect (%)	MeOH Recovery (%)	MeOH RSD (%)	MeOH Matrix effect (%)
Bromazepam	57	15	22	56	16	24	69	22	98	65	3	80
Caffeine	69	3	16	65	19	27	65	5	7	65	7	23
Carazolol	66	30	-52	59	11	-21	65	10	10	46	2	17
Carbamazepine	79	5	10	68	22	-8	64	13	-19	67	4	-3
Carbamazepine-10,11-epoxide	74	3	24	64	19	38	68	9	32	67	3	42
Chloramphenicol	83	1	0	65	22	4	59	9	19	68	4	-5
Chlorpromazine	63	32	-62	69	32	-92	48	14	14	38	7	13
Ciprofloxacin	23	13	-9	60	15	14	65	12	-1	6	23	35
Citalopram	69	33	-57	61	10	-38	72	14	-7	56	4	-13
Clarithromycin	69	24	-32	60	16	-15	73	15	-17	61	2	-26
Clofibric acid	49	3	11	57	22	13	49	14	40	67	1	26
Cocaethylene	70	21	-22	65	18	-11	74	13	-18	65	6	-14
Cocaine	73	17	-10	65	21	-8	73	14	-28	65	6	-19
Codeine	62	6	43	63	17	29	72	8	-6	61	1	32
Cotinine	64	6	15	59	21	-3	68	2	-35	72	7	-6
Diazepam	78	12	-28	66	12	-36	62	14	-22	68	3	-26
Diclofenac	82	2	-1	64	7	16	79	11	37	88	5	47
Diltiazem	70	32	-52	61	13	-29	73	11	-7	61	4	-12
Erythromycin	69	9	-6	69	20	-40	57	22	-88	66	3	-24
Fenofibrate	67	31	-69	88	22	-90	39	19	-42	66	2	-15
Fipronil Desulfynil	70	18	-51	62	9	-82	55	23	-23	79	3	-2
Fipronil Sulfide	70	18	-51	62	9	-82	55	24	-23	79	3	-2
Fipronil Sulfone	66	19	-48	64	5	-85	50	3	-38	75	5	12
Fipronil	75	15	-28	67	4	-52	59	4	-13	71	4	-2
Fluconazole	75	2	16	63	24	13	71	12	6	66	1	15
Flumequine	67	2	19	66	22	4	57	11	24	38	4	18
Fluoxetine	62	32	-63	76	14	-86	66	14	29	47	3	14
Furazolidone	75	4	23	73	20	7	52	13	4	65	9	-25
Furosemide	52	16	2	50	15	-34	35	12	7	48	11	-16
Hydrochlorothizade	79	1	10	61	22	3	54	9	7	59	2	-5
Indomethacin	66	4	-3	62	11	-34	28	24	-34	62	3	-16
Irgasan	62	22	-13	89	30	-81	21	49	-70	56	5	104
Ketoprofen	56	9	47	64	25	34	57	11	25	70	1	30
Lamotrigine	77	11	-35	65	17	-44	66	11	-49	67	9	-47
Loratadine	65	29	-63	82	17	-87	76	14	-9	63	4	-13
Lorazepam	71	10	-18	61	16	-18	56	4	59	63	2	22
Losartan potassium	67	9	-18	63	10	-60	62	11	-50	65	2	-38
Mefenamic acid	68	2	-12	70	1	-64	19	28	-47	56	6	14
Metformin	43	15	-84	58	4	-94	60	10	-95	63	12	-95
Methadone	63	35	-67	56	5	-61	71	13	-5	57	4	-18
Metoprolol	66	9	-1	58	18	34	75	10	27	64	3	34
Midazolam	62	15	-49	55	7	-63	73	12	-15	58	3	-29
Morphine	61	4	-15	49	32	-38	68	2	-54	58	12	-51
N-acethyl SMX	61	4	17	62	23	16	70	12	25	68	3	14
Nalidixic acid	58	5	14	66	16	-7	62	14	-10	37	4	-4
N-Desmethylcitalopram	67	32	-60	60	9	-46	71	14	-2	53	2	-13
Norfluoxetine	72	30	-66	83	14	-86	65	13	110	44	4	52
O-desmethylvenlafaxine	68	11	1	74	4	1	72	15	-38	68	3	-12
Omeprazole	69	3	25	70	27	-68	0	-	-	65	3	99
Oseltamivir phosphate	66	16	-33	49	15	36	72	11	227	67	2	96
Oseltamivir-CBX	14	14	17	52	15	25	70	13	65	64	5	48
Oxazepam	69	12	-15	59	19	-15	56	6	66	61	1	21
Oxytetracycline	20	11	17	20	12	19	58	22	12	0	-	-
Paroxetine	63	29	-60	72	12	-82	57	15	73	36	2	49
Pentobarbital	83	6	-3	87	15	-8	79	11	-12	86	3	-3
Propyphenazone	80	2	6	66	21	-9	69	13	-15	70	3	-14
Salbutamol	50	7	57	62	17	52	74	9	-1	65	2	40
Salicylic acid	55	2	-2	51	14	1	161	58	148	65	11	25
Sertraline	74	33	-41	104	11	-85	65	16	68	42	2	81
Sitagliptin	73	15	-22	58	19	7	65	8	122	56	5	29
Sotalol	64	3	41	64	20	36	71	7	5	65	3	24
Sulfadimethoxine	84	2	25	64	24	23	68	18	28	66	3	61
Sulfamethazine	87	0.2	10	67	23	13	76	20	-5	66	5	86
Sulfamethoxazole	82	2	13	65	24	8	71	19	-7	66	2	31
Sulfapyridine	78	1	14	64	25	1	73	18	-31	64	4	72
Temazepam	77	8	65	66	18	53	57	13	87	66	4	76
Tramadol	73	8	-3	65	22	-4	74	11	-30	65	4	-14
Triclocarban	66	33	153	61	20	-74	15	55	-17	38	3	842
Trimethoprim	66	14	-9	61	20	-2	71	6	-26	61	1	-11
Valsartan	15	8	39	48	21	-4	53	15	-23	64	2	24
Valsartan acid	4	7	8	42	16	-17	47	6	-16	62	3	-5
Venlafaxine	71	19	-24	62	17	-9	77	10	-24	64	2	-15
Verapamil	67	34	-52	56	7	-34	71	12	23	59	3	4
Warfarin	84	2	12	69	26	22	50	12	151	70	3	50
Zolpidem	45	17	13	59	17	-14	75	12	-22	66	3	-16

CECs	CLEAN-UP STEP TEST – *Procambarus clarkii* – Z-Sep Recovery (%)	Z-Sep RSD (%)	Z-Sep Matrix effect (%)	Z-Sep+ Recovery (%)	Z-Sep+ RSD (%)	Z-Sep+ Matrix effect (%)	Z-Sep/C18 Recovery (%)	Z-Sep/C18 RSD (%)	Z-Sep/C18 Matrix effect (%)
Bromazepam	82	20	-78	36	21	13	41	22	-62
Caffeine	100	11	59	94	3	88	93	3	71
Carazolol	78	31	-85	26	7	-34	107	23	-84
Carbamazepine	77	11	-26	81	4	-25	85	5	-29
Carbamazepine-10,11-epoxide	78	1	-6	67	4	2	82	4	-3
Chloramphenicol	77	3	-25	56	7	-30	89	14	-33
Chlorpromazine	125	41	-95	24	18	-67	124	31	-93
Ciprofloxacin	0	-	-34	0	-	-22	0	-	-31
Citalopram	78	33	-85	68	5	-35	100	29	-85
Clarithromycin	66	30	-79	17	9	-39	87	31	-80
Clofibric acid	14	4	-19	3	20	-27	30	5	-26
Cocaethylene	66	25	-53	69	4	-36	88	18	-62
Cocaine	66	16	-40	73	5	-34	80	10	-49
Codeine	54	17	-30	62	4	-36	62	6	-43
Cotinine	59	14	-36	58	7	-47	58	9	-38
Diazepam	80	23	-61	91	6	-40	89	11	-64
Diclofenac	44	10	-23	4	12	-18	53	11	-24
Diltiazem	72	38	-81	60	6	-35	104	31	-84
Erythromycin	60	10	-61	26	11	-40	78	7	-62
Fenofibrate	89	21	-84	66	19	-31	130	0.4	-81
Fipronil Desulfynil	87	39	-51	50	17	16	106	19	-57
Fipronil Sulfide	82	34	-48	50	17	23	101	10	-54
Fipronil Sulfone	83	34	-53	51	17	14	111	25	-61
Fipronil	78	16	-45	62	5	-5	97	16	-41
Fluconazole	51	15	-16	16	10	-8	70	8	-14
Flumequine	0.1	1	-7	0	-	-9	0.2	30	-7
Fluoxetine	124	44	-96	52	9	-63	147	23	-94
Furazolidone	73	3	-13	91	6	-20	73	5	-9
Furosemide	12	10	-25	16	9	-35	34	13	-31
Hydrochlorothizade	83	6	-23	86	7	-31	90	6	-27
Indomethacin	31	14	-16	0	-	-18	43	13	-20
Irgasan	59	4	89	29	1	287	93	5	-21
Ketoprofen	30	19	61	3	19	57	37	0	72
Lamotrigine	69	20	-54	97	4	-49	86	7	-60
Loratadine	91	14	-95	53	10	-65	115	17	-93
Lorazepam	41	16	-22	1	46	12	69	10	-17
Losartan potassium	60	13	75	53	10	117	66	12	75
Mefenamic acid	12	25	-31	1	14	-16	21	11	-33
Metformin	11	2	-95	30	9	-93	19	25	-97
Methadone	61	9	-92	45	8	-35	118	35	-92
Metoprolol	61	9	-40	14	10	-28	73	7	-45
Midazolam	71	55	-81	45	28	-41	65	12	-80
Morphine	31	6	-51	20	5	-54	56	5	-64
N-acethyl SMX	53	4	-17	44	7	-2	52	3	-16
Nalidixic acid	0	2	-29	0	3	-34	0	28	-34
N-Desmethylcitalopram	76	38	-87	69	4	-37	117	32	-88
Norfluoxetine	136	55	-96	51	9	-60	119	10	-92
O-desmethylvenlafaxine	60	10	-35	22	9	-37	63	3	-37
Omeprazole	67	9	-46	49	10	8	80	9	-37
Oseltamivir phosphate	0	-	-11	0	-	4	0	-	-10
Oseltamivir-CBX	60	17	-55	17	4	41	70	12	-57
Oxazepam	35	22	-22	3	10	13	66	11	-20
Oxytetracycline	0	-	-37	0	-	53	0	-	0.3
Paroxetine	0	-	-98	42	10	-54	141	4	-95
Pentobarbital	89	15	-17	78	5	-12	80	7	-9
Propyphenazone	79	10	-32	60	3	-34	83	3	-34
Salbutamol	15	38	-17	5	19	-26	28	2	-17
Salicylic acid	7	5	-42	0	-	-38	15	15	-39
Sertraline	128	60	-94	14	14	-75	120	25	-91
Sitagliptin	67	20	-46	65	5	-9	88	13	-52
Sotalol	58	12	-51	61	6	-49	69	2	-61
Sulfadimethoxine	70	3	-36	70	8	-21	71	4	-30
Sulfamethazine	96	48	-32	70	9	-14	78	2	-29
Sulfamethoxazole	69	8	-2	56	9	17	70	0.2	4
Sulfapyridine	71	5	-48	67	7	-26	80	7	-46
Temazepam	44	23	18	3	20	33	75	6	18
Tramadol	67	10	-31	35	7	-24	75	5	-35
Triclocarban	146	33	217	36	43	200	45	2	44
Trimethoprim	47	30	-49	71	3	-39	74	9	-52
Valsartan	1	55	77	2	50	74	5	18	102
Valsartan acid	0	-	-43	0	-	-51	0	-	-44
Venlafaxine	63	21	-52	15	5	-30	73	18	-58
Verapamil	87	38	-91	89	6	-36	132	34	-91
Warfarin	76	7	-7	25	1	-16	69	4	-0.2
Zolpidem	73	17	-46	85	5	-36	84	9	-50

CECs	CLEAN-UP STEP TEST – *Anodonta cygnea* – Z-Sep Recovery (%)	Z-Sep RSD (%)	Z-Sep Matrix effect (%)	Z-Sep+ Recovery (%)	Z-Sep+ RSD (%)	Z-Sep+ Matrix effect (%)	Z-Sep/C18 Recovery (%)	Z-Sep/C18 RSD (%)	Z-Sep/C18 Matrix effect (%)
Bromazepam	119	13	-86	53	3	1	64	8	-4
Caffeine	65	6	5	63	5	4	61	7	16
Carazolol	74	6	-70	27	5	0	62	2	-33
Carbamazepine	64	3	-18	47	5	1	59	0.3	1
Carbamazepine-10,11-epoxide	65	3	-4	58	2	10	60	3	25
Chloramphenicol	60	12	-31	49	8	-21	64	8	-34
Chlorpromazine	134	5	-92	23	9	-24	48	5	-60
Ciprofloxacin	2	13	-65	1	38	-3	1	1	19
Citalopram	109	4	-81	51	6	-7	64	1	-45
Clarithromycin	113	5	-74	23	5	-12	55	5	-27
Clofibric acid	27	16	-31	9	11	-13	55	10	-22
Cocaethylene	103	4	-61	47	5	-8	55	2	-17
Cocaine	91	4	-50	48	4	-2	54	2	-9
Codeine	58	6	-5	51	2	14	55	4	11
Cotinine	61	3	-39	49	3	-23	56	5	-36
Diazepam	71	1	-49	57	5	-9	52	6	-30
Diclofenac	41	11	-28	6	16	-21	48	6	-21
Diltiazem	114	4	-79	48	6	-11	61	2	-40
Erythromycin	93	4	-62	34	5	-8	57	2	-18
Fenofibrate	112	20	-77	69	1	6	39	5	-45
Fipronil Desulfynil	117	26	-58	74	23	26	57	3	18
Fipronil Sulfide	111	19	-56	80	12	17	57	3	25
Fipronil Sulfone	112	14	-62	78	25	12	55	5	5
Fipronil	100	8	-48	67	10	1	60	7	4
Fluconazole	49	8	1	16	9	11	53	3	30
Flumequine	1	25	-62	0	37	-17	2	8	18
Fluoxetine	130	20	-92	43	10	-19	61	1	-69
Furazolidone	56	2	7	53	7	8	51	2	19
Furosemide	17	17	-40	19	14	-21	35	9	-38
Hydrochlorothizade	62	2	-25	63	2	-17	61	4	-25
Indomethacin	38	27	-30	0	-	-14	49	1	-17
Irgasan	122	1	82	31	11	139	54	14	367
Ketoprofen	38	14	50	5	11	15	50	3	45
Lamotrigine	64	2	-47	51	4	-11	53	4	-32
Loratadine	129	19	-91	46	5	-29	42	9	-74
Lorazepam	61	8	-52	3	29	-22	52	5	-12
Losartan potassium	58	4	-19	44	6	7	52	7	-18
Mefenamic acid	21	12	-49	1	10	-22	27	3	-18
Metformin	46	15	-91	31	7	-85	41	9	-92
Methadone	120	8	-89	36	10	-13	61	2	-62
Metoprolol	63	4	-26	20	4	3	58	2	2
Midazolam	89	5	-79	35	4	-18	44	3	-46
Morphine	48	3	-55	24	9	-28	48	2	-55
N-acethyl SMX	63	5	-6	65	2	4	62	3	6
Nalidixic acid	1	7	-68	1	15	-25	1	8	-4
N-Desmethylcitalopram	99	6	-81	49	4	-4	65	2	-48
Norfluoxetine	122	14	-90	48	5	-13	61	4	-55
O-desmethylvenlafaxine	65	4	-31	64	1	-4	56	2	-5
Omeprazole	61	13	-51	48	6	8	50	12	-18
Oseltamivir phosphate	11	40	-3	22	7	10	37	3	14
Oseltamivir-CBX	68	4	-35	46	2	14	61	4	14
Oxazepam	53	14	-50	4	10	-21	52	1	-13
Oxytetracycline	0	-	1	0	-	4	0	-	46
Paroxetine	124	22	-90	41	8	-16	65	3	-62
Pentobarbital	58	12	-16	62	1	-4	62	5	-11
Propyphenazone	64	3	-19	32	8	1	55	2	-4
Salbutamol	32	17	2	19	5	27	51	4	19
Salicylic acid	9	3	-41	17	6	-31	36	18	-42
Sertraline	153	16	-87	20	4	-26	55	7	-38
Sitagliptin	68	3	-31	54	3	5	60	5	0
Sotalol	60	2	-6	53	0	10	57	4	15
Sulfadimethoxine	69	3	-32	69	4	7	63	6	-7
Sulfamethazine	66	3	-30	73	4	1	63	10	-5
Sulfamethoxazole	64	3	-5	72	4	15	61	6	18
Sulfapyridine	68	2	-28	68	5	12	64	15	-0.4
Temazepam	58	9	-23	2	14	17	51	3	36
Tramadol	69	2	-22	31	5	12	55	2	10
Triclocarban	212	59	120	106	32	867	51	40	708
Trimethoprim	64	4	-33	56	4	-10	58	4	-11
Valsartan	9	55	-13	13	3	-16	30	11	8
Valsartan acid	0	-	-47	54	8	-26	28	12	-30
Venlafaxine	87	3	-52	16	6	0	56	3	-10
Verapamil	115	7	-86	60	6	-9	66	4	-54
Warfarin	58	9	-14	23	8	-22	59	8	-15
Zolpidem	86	2	-52	49	5	-8	54	2	-17

*Nicotine — The nicotine standard was not available during the method development experiments, so it was only included in the validation.

Table S4. Validation parameters

Procambarus clarkii

CECs		10 ng g^{-1} Recovery (%)	10 ng g^{-1} RSD (%)	10 ng g^{-1} Matrix Effect (%)	100 ng g^{-1} Recovery (%)	100 ng g^{-1} RSD (%)	100 ng g^{-1} Matrix Effect (%)	LOD (ng g^{-1})	LOQ (ng g^{-1})
OPFRs	TPP	151	30	114	144	29	120	0.04	0.12
	TDBPP	105	79	146	92	82	152	0.19	0.57
	CDP	<LOQ	-	-	75	11	-86	4.00	12.00
	TnBP	132	15.5	103	149	18	109	0.05	0.14
	TBEP	109	8	50	122	7	56	0.06	0.17
	TMPP	72	32	74	101	35	80	0.08	0.25
	TEHP	65	14	133	120	13	139	0.31	0.93
	TCEP	112	-0.2	148	113	3	154	0.05	0.16
	TClPP	100	12	60	113	11	66	0.40	1.20
	TDClPP	48	26	-78	80	29	-72	0.42	1.26
	TPhP	99	1	53	112	2	59	0.06	0.18
PFAS	PFPeA	123	45	103	136	14	146	1.5	4.4
	PFBS	89	31	-55	102	17	-12	0.3	0.8
	PFHpA	96	19	-3	109	11	40	0.1	0.3
	PFHpS	89	42	-20	102	12	23	0.1	0.2
	ipPFNA	89	25	-28	102	14	15	0.7	2.0
	FOUEA	99	20	-8	112	12	35	1.5	4.4
	ipPFN	38	13	-44	71	18	-21	0.2	0.5
	PFDS	47	18	-54	50	11	-41	2.9	8.7
	PFTrDA	79	17	-61	102	10	-38	0.1	0.2
	PFTeDA	<LOQ	-	-	87	18	-46	4.1	12.3
	PFHxDA	69	8	-58	101	15	-45	0.1	0.3
	PFODA	95	8	-63	98	15	-30	1.8	5.5
	PFBA	101	20	51	104	11	94	0.9	2.7
	PFHxA	81	44	-10	95	14	33	0.3	1.0
	PFHxS	120	16	-11	92	10	32	0.0	0.0
	PFOA	115	12	55	105	17	98	0.1	0.2
	PFOS	120	24	-47	103	14	-4	0.3	0.8
	PFNA	89	25	-37	102	14	6	0.9	2.7
	PFDA	94	17	14	107	10	57	0.1	0.2
	PFUnDA	47	11	-53	90	17	-30	0.1	0.4
	PFDoDA	<LOQ	-	-	84	19	-44	4.3	12.9
PESTICIDES	Acetamiprid	59	29	148	104	24	155	1.0	3.0
	Alachlor	67	12	89	120	15	96	0.5	1.5
	Atrazine	41	3	56	68	8	63	1.0	5.0
	Atrazine-Deethyl	77	3.2	45	110	6.2	52	1.2	3.5
	Atrazine-Deisoproprył	81	29	32	108	15	39	1.1	3.3
	Azinphos-ethyl	<LOQ	-	-92	65	17	-85	5.0	15.0
	Azinphos-Methyl	33	32	121	51	17	128	3.1	9.2
	Buprofezine	<LOQ	-	-86	56	18	-79	5.0	15.0
	Carbendazim	61	5	81	108	10	88	0.6	1.8
	Carbofuran	35	-	67	45	19	74	2.0	6.0
	Carbofuran 3-OH	41	34	148	68	19	155	2.2	6.6
	Chlorfenvinphos	<LOQ	-	105	87	15	212	4.7	14.1
	Chlorpyriphos	73	5	12	102	10	19	2.2	6.6
	Chlothianidin	67	35	103	119	18	140	2.4	7.3
	Coumaphos	<LOQ	-	-9	62	15	-2	4.0	12.0
	Diazinon	<LOQ	-	-90	72	12	-83	3.8	11.4
	Dichlofenthion	<LOQ	-	-83	75	13	-76	5.0	15.0
	Dimethoate	66	7	-5	118	10	2	0.9	2.7
	Diuron	<LOQ	-	-73	86	15	-66	4.1	12.3
	DMA	<LOQ	-	-86	73	18	-79	4.8	14.4
	DMF	84	7	-58	119	12	-51	1.2	3.6
	DMPF	73	4	81	103	7	88	1.5	4.5
	Ethion	127	16	-21	125	21	-14	0.3	0.9
	Etofenprox	<LOQ	-	-91	87	17	-84	3.7	11.1
	Fenthion	<LOQ	-	-8	85	10	-1	4.5	14.5
	Fenthion sulfone	53	35	-60	92	22	-53	0.7	2.0
	Fenthion sulfoxide	72	34	175	87	19	182	0.7	2.1
	Fipronil	47	23	-80	109	18	-73	0.2	0.5
	Hexythiazox	56	17	-86	98	22	-79	0.9	2.7
	Imazalil	<LOQ	-	-89	88	22	-82	4.0	12.0
	Imidacloprid	61	39	55	107	44	62	1.5	4.5
	Isoproturon	61	30	45	108	33	52	0.8	2.5
	Isoproturon	13	13	-55	12	18	-48	2.0	6.0
	Methiocarb	52	12	119	90	15	206	1.2	3.5
	Methoalachlor	53	13	106	72	18	183	1.4	4.2
	Molinate	107	15	-46	98	18	-39	0.6	1.7
	Omethoate	<LOQ	-	-85	72	15	-78	5.0	15.0
	Parathion-ethyl	<LOQ	-	-73	78	12	-66	5.0	15.0
	Prochloraz	<LOQ	-	4	76	10	11	4.0	12.0
	Propanil	<LOQ	-	-91	73	18	-84	4.0	12.0
	Propazine	63	39	75	69	17	82	0.8	2.4
	Pyriproxyfen	<LOQ	-	-77	71	18	-70	3.5	10.5
	Simazine	74	39	-26	73	12	-19	0.8	2.4
	Spinosad A	40	42	105	55	10	152	2.0	6.0
	Spinosad C	39	12	-91	64	17	-84	1.5	4.6
	Spinosad D	81	36	110	102	19	147	0.7	2.2
	Tebuconazole	53	4	-77	61	9	-70	1.8	5.5
	Terbumeton	53	33	-75	92	16	-68	0.1	0.3
	Terbumeton deethyl	76	11	118	108	16	155	0.1	0.2
	Terbuthylazine	57	23	16	100	12	23	0.1	0.3
	Terbuthylazine-2OH	98	12	80	103	17	87	0.6	1.8
	Terbuthylazine-deethyl	97	3	46	99	6	53	0.6	1.9
	Terbutryn	48	4	31	81	9	38	1.3	3.8
	Thiabendazole	73	13	103	101	16	150	0.8	2.5
	Thiametosan	61	29	-40	107	14	-33	2.0	5.9
PhACs	5-methyl-1H-Benzotriazole	99	1	-35	78	2	-53	0.30	1.02
	Acetaminophen	97	1	-82	79	1	-88	0.68	2.25
	Acridone	113	0.2	-38	87	1.8	-45	0.45	1.51
	Alprazolam	98	3	-23	99	4	-46	0.18	0.59

Anodonta cygnea

CECs		10 ng g^{-1} Recovery (%)	10 ng g^{-1} RSD (%)	10 ng g^{-1} Matrix Effect (%)	100 ng g^{-1} Recovery (%)	100 ng g^{-1} RSD (%)	100 ng g^{-1} Matrix Effect (%)	LOD (ng g^{-1})	LOQ (ng g^{-1})
OPFRs	TPP	134	21	-89	144	24	-85	0.04	0.13
	TDBPP	28	15	-62	38	16	-60	0.71	2.14
	CDP	<LOQ	-	-	72	17	-81	4.00	12.00
	TnBP	131	23.5	-84	141	19.5	-82	0.05	0.14
	TBEP	90	2	-43	100	6	-39	0.07	0.20
	TMPP	71	17	-92	81	13	-90	0.08	0.25
	TEHP	77	14	-67	87	17	-63	0.26	0.78
	TCEP	103	3	171	113	2	173	0.06	0.18
	TClPP	103	3	-44	113	6	-40	0.39	1.17
	TDClPP	103	2	-92	113	2	-90	0.19	0.58
	TPhP	103	3	38	113	5	42	0.06	0.17
PFAS	PFPeA	75	16	152	88	8	195	2.4	7.2
	PFBS	98	23	-57	101	11	-14	0.2	0.7
	PFHpA	86	24	37	99	12	80	0.3	1.0
	PFHpS	73	30	-33	106	15	10	0.1	0.2
	ipPFNA	79	17	-18	92	9	25	0.8	2.3
	FOUEA	89	21	-6	92	11	37	2.0	6.0
	ipPFN	<LOQ	-	-	84	10	-41	4.1	12.3
	PFDS	76	27	-88	109	23	-45	1.8	5.4
	PFTrDA	87	9	-83	100	4	-40	0.0	0.1
	PFTeDA	<LOQ	-	-	87	11	-46	4.5	13.5
	PFHxDA	<LOQ	-	-	89	14	-47	4.3	12.9
	PFODA	106	11	-75	99	5	-32	1.7	5.0
	PFBA	70	23	153	93	11	196	1.3	3.8
	PFHxA	100	13	35	93	7	78	1.0	3.0
	PFHxS	96	22	-0.3	99	11	43	0.0	0.0
	PFOA	99	8	86	92	4	129	0.1	0.2
	PFOS	81	32	-59	94	16	-16	0.4	1.2
	PFNA	83	16	-16	96	8	27	1.0	2.9
	PFDA	99	10	3	94	5	46	0.1	0.2
	PFUnDA	72	22	-81	95	11	-38	0.1	0.2
	PFDoDA	<LOQ	-	-	89	5.7	-46	4.3	12.9
PESTICIDES	Acetamiprid	83	35	2	89	36	8	2.5	7.5
	Alachlor	<LOQ	-	-86	62	15	-80	3.4	10.2
	Atrazine	146	36	-79	152	36	-73	0.6	1.9
	Atrazine-Deethyl	63	9.8	-20	69	10.8	-14	1.4	4.3
	Atrazine-Deisoproprył	70	15	5	76	16	11	1.3	3.8
	Azinphos-ethyl	<LOQ	-	-89	65	14	-83	5.0	15.0
	Azinphos-Methyl	<LOQ	-	-78	59	12	-72	4.0	12.0
	Buprofezine	<LOQ	-	-74	64	14	-68	5.2	15.6
	Carbendazim	68	27	0	74	28	6	0.5	1.6
	Carbofuran	51	13	-43	57	14	-37	1.2	3.5
	Carbofuran 3-OH	82	33	37	88	18	43	1.1	3.3
	Chlorfenvinphos	54	-	-83	60	12	-77	3.0	9.0
	Chlorpyriphos	103	26	142	100	27	148	1.1	3.4
	Chlothianidin	79	10	101	85	11	107	2.0	6.1
	Coumaphos	<LOQ	-	-	67	10	-71	4.0	12.0
	Diazinon	<LOQ	-	-	73	13	-76	3.8	11.4
	Dichlofenthion	<LOQ	-	-	74	14	-79	5.0	15.0
	Dimethoate	101	24	-66	107	25	-60	0.6	1.8
	Diuron	<LOQ	-	-	82	15	-77	4.1	12.3
	DMA	<LOQ	-	-	79	12	-86	4.8	14.4
	DMF	53	24	-48	59	25	-42	1.8	5.4
	DMPF	120	19	69	106	20	75	3.0	9.0
	Ethion	48	37	-31	54	17	-25	0.8	2.3
	Etofenprox	<LOQ	-	-	65	16	-77	3.7	11.1
	Fenthion	<LOQ	-	-	81	15	-76	4.5	14.5
	Fenthion sulfone	66	29	-42	72	20	-36	0.5	1.6
	Fenthion sulfoxide	77	34	139	83	14	145	0.6	1.9
	Fipronil	38	23	-91	44	24	-85	0.2	0.5
	Hexythiazox	65	22	-88	71	23	-82	0.8	2.3
	Imazalil	<LOQ	-		82	18	-74	4.5	13.5
	Imidacloprid	93	13	146	99	13	152	1.0	2.9
	Isoproturon	62	10	-1	68	11	5	0.8	2.4
	Isoproturon	102	15	-15	108	16	-9	0.3	0.9
	Methiocarb	84	15	50	90	16	56	0.7	2.1
	Methoalachlor	124	29	-66	100	19	-60	0.5	1.4
	Molinate	<LOQ	-	-	76	15	-82	3.5	10.5
	Omethoate	<LOQ	-	-	82	14	-83	5.0	15.0
	Parathion-ethyl	<LOQ	-	-	72	12	-86	5.0	15.0
	Prochloraz	<LOQ	-	-	79	10	-71	4.0	12.0
	Propanil	<LOQ	-	-	76	9	-76	4.0	12.0
	Propazine	112	13	-37	118	14	-31	0.4	1.3
	Pyriproxyfen	<LOQ	-	-	89	8	-80	3.5	10.5
	Simazine	85	11	-68	91	11	-62	0.7	2.1
	Spinosad A	100	16	-88	106	17	-82	0.4	1.3
	Spinosad C	79	16	129	85	17	135	0.8	2.3
	Spinosad D	72	12	34	80	8	-52	1.1	3.3
	Tebuconazole	87	8	-27	93	8	-21	0.7	2.1
	Terbumeton	113	39	-21	109	40	-15	0.1	0.2
	Terbumeton deethyl	92	21	129	98	22	135	0.1	0.2
	Terbuthylazine	104	10	-61	110	11	-55	0.1	0.2
	Terbuthylazine-2OH	103	23	125	109	23	131	0.6	1.7
	Terbuthylazine-deethyl	106	7	134	112	8	140	0.6	1.7
	Terbutryn	101	19	-76	107	20	-70	0.5	1.4
	Thiabendazole	65	5	97	71	6	103	0.9	2.8
	Thiametosan	85	24	132	91	25	138	1.4	4.2
PhACs	5-methyl-1H-Benzotriazole	94	5	-18	109	9	-29	0.05	0.16
	Acetaminophen	94	3	-34	104	3	-45	0.18	0.62
	Acridone	96	2.5	-16	114	3.6	-35	0.07	0.23
	Alprazolam	97	1	-36	118	7	-37	0.06	0.20

Leopomis gibbosus

CECs		10 ng g^{-1} Recovery (%)	10 ng g^{-1} RSD (%)	10 ng g^{-1} Matrix Effect (%)	100 ng g^{-1} Recovery (%)	100 ng g^{-1} RSD (%)	100 ng g^{-1} Matrix Effect (%)	LOD (ng g^{-1})	LOQ (ng g^{-1})
OPFRs	TPP	70	11	140	76	14	142	0.09	0.26
	TDBPP	142	7	126	148	9	128	0.14	0.42
	CDP	<LOQ	-	-	71	35	-84	4.00	12.00
	TnBP	97	9.1	125	103	11.1	127	0.06	0.19
	TBEP	100	17	133	106	20	135	0.06	0.18
	TMPP	98	22	72	104	24	74	0.06	0.18
	TEHP	104	31	113	110	34	115	0.19	0.58
	TCEP	104	9	136	110	11	138	0.06	0.17
	TClPP	99	12	54	105	15	56	0.41	1.22
	TDClPP	83	38	-80	89	40	-78	0.24	0.72
	TPhP	105	2	52	111	5	54	0.06	0.17
PFAS	PFPeA	92	22	79	98	28	83	2.0	5.9
	PFBS	116	29	-59	122	34	-55	0.2	0.6
	PFHpA	72	6	2	78	12	6	0.4	1.2
	PFHpS	85	9	-28	91	14	-24	0.1	0.2
	ipPFNA	74	10	-53	80	16	-49	0.8	2.4
	FOUEA	87	26	-44	93	31	-40	1.2	3.6
	ipPFN	50	-	-95	56	18	-50	0.1	0.4
	PFDS	108	4	-88	114	9	-64	1.3	3.8
	PFTrDA	100	2	-83	106	4	-49	0.04	0.1
	PFTeDA	<LOQ	-	-	86	10	0.10	4.5	13.5
	PFHxDA	<LOQ	-	-	85	12	-55	4.3	12.9
	PFODA	107	7	-73	113	2	-69	1.6	4.9
	PFBA	85	16	50	91	22	54	1.1	3.2
	PFHxA	82	-	25	88	5	29	1.2	3.6
	PFHxS	77	9	-13	83	15	-9	0.01	0.0
	PFOA	90	26	51	96	31	55	0.1	0.2
	PFOS	52	35	-58	58	41	-54	0.6	1.9
	PFNA	69	13	-54	75	18	-50	1.2	3.5
	PFDA	97	5	-9	103	11	-5	0.1	0.2
	PFUnDA	77	7	-86	83	12	-62	0.1	0.2
	PFDoDA	<LOQ	-	-87	82	10.0	-83	4.3	12.9
PESTICIDES	Acetamiprid	118	28	117	104	28	123	2.3	7.0
	Alachlor	99	10	152	105	17	158	5.2	15.7
	Atrazine	114	63	10	120	23	16	0.8	2.4
	Atrazine-Deethyl	116	4.5	32	102	11.5	38	0.7	2.0
	Atrazine-Deisoproprył	119	32	7	125	22	13	0.8	2.3
	Azinphos-ethyl	<LOQ	-	-	<LOQ	-	-70	5.0	15.0
	Azinphos-Methyl	112	4	148	118	4	154	0.9	2.7
	Buprofezine	<LOQ	-	-	<LOQ	-	-68	5.2	15.6
	Carbendazim	125	4	35	101	4	41	0.3	0.9
	Carbofuran	79	12	74	85	19	80	0.8	2.3
	Carbofuran 3-OH	113	3	147	119	3	153	0.8	2.4
	Chlorfenvinphos	76	-	148	82	15	154	2.1	6.4
	Chlorpyriphos	44	25	-51	50	25	-45	3.7	11.2
	Chlothianidin	86	12	103	92	19	109	1.9	5.7
	Coumaphos	108	14	-62	104	14	-56	0.6	1.8
	Diazinon	<LOQ	-	-	74	10	-67	3.8	11.4
	Dichlofenthion	<LOQ	-	-	72	10	-86	5.0	15.0
	Dimethoate	100	15	-34	106	22	-28	0.4	1.2
	Diuron	<LOQ	-	-	82	12	-71	4.1	12.3
	DMA	<LOQ	-	-	80	11	-77	4.8	14.4
	DMF	36	9	-64	42	9	-58	1.0	3.1
	DMPF	112	6	40	118	13	46	3.2	9.7
	Ethion	96	3	26	102	3	32	0.4	1.1
	Etofenprox	<LOQ	-	-	75	14	-86	3.7	11.1
	Fenthion	<LOQ	-	-	104	11	-84	4.5	14.5
	Fenthion sulfone	104	5	148	110	12	154	0.3	1.0
	Fenthion sulfoxide	107	4	142	103	4	148	0.4	1.2
	Fipronil	100	7	147	106	14	153	0.0	0.1
	Hexythiazox	54	11	-83	60	11	-77	0.9	2.8
	Imazalil	<LOQ	-	-	78	15	-71	4.5	13.5
	Imidacloprid	83	7	147	89	7	153	1.1	3.2
	Isoproturon	124	5	148	120	12	154	0.4	1.2
	Isoproturon	71	1	115	77	1	121	0.7	2.1
	Methiocarb	63	16	199	69	23	205	0.9	2.8
	Methoalachlor	107	7	39	113	7	45	0.6	1.7
	Molinate	103	15	-68	109	22	-62	0.4	1.1
	Omethoate	<LOQ	-	-	72	17	-78	5.0	15.0
	Parathion-ethyl	<LOQ	-	-	79	16	-82	5.0	15.0
	Prochloraz	124	12	-42	100	18	-66	2.8	8.4
	Propanil	<LOQ	-	-	<LOQ	14	-73	4.0	12.0
	Propazine	120	17	19	106	17	25	0.4	1.2
	Pyriproxyfen	<LOQ	-	-	<LOQ	15	-59	3.5	10.5
	Simazine	113	6	-17	119	6	-11	0.5	1.6
	Spinosad A	63	16	148	69	23	154	0.9	2.8
	Spinosad C	102	12	136	108	12	142	0.6	1.8
	Spinosad D	65	10	144	71	17	150	0.9	2.8
	Tebuconazole	57	7	138	63	7	144	1.1	3.2
	Terbumeton	93	7	134	99	14	140	0.1	0.2
	Terbumeton deethyl	91	16	141	97	16	147	0.1	0.2
	Terbuthylazine	121	12	-28	107	19	-22	0.0	0.1
	Terbuthylazine-2OH	105	7	82	111	7	88	0.6	1.7
	Terbuthylazine-deethyl	98	4	46	104	11	52	0.6	1.8
	Terbutryn	125	7	-23	101	7	-17	0.5	1.4
	Thiabendazole	80	15	147	86	22	153	0.8	2.3
	Thiametosan	92	15	-18	98	15	-12	1.3	3.9
PhACs	5-methyl-1H-Benzotriazole	92	13	-39	94	10	-43	0.06	0.19
	Acetaminophen	69	10	-24	82	17	-43	0.17	0.57
	Acridone	101	11.5	-25	92	8.5	-41	0.07	0.24
	Alprazolam	96	22	10	97	10	-24	0.29	0.96

Corbicula fluminea

CECs		10 ng g^{-1} Recovery (%)	10 ng g^{-1} RSD (%)	10 ng g^{-1} Matrix Effect (%)	100 ng g^{-1} Recovery (%)	100 ng g^{-1} RSD (%)	100 ng g^{-1} Matrix Effect (%)	LOD (ng g^{-1})	LOQ (ng g^{-1})
OPFRs	TPP	70	9	-82	76	11	-76	0.09	0.26
	TDBPP	137	10	-1	143	10	5	0.15	0.44
	CDP	<LOQ	-	-	69	13	-78	4.00	12.00
	TnBP	127	28.0	-85	133	28.0	-79	0.05	0.14
	TBEP	133	36	-73	139	38	-67	0.05	0.14
	TMPP	66	11	-89	72	11	-83	0.09	0.27
	TEHP	44	34	-66	50	36	-60	0.45	1.36
	TCEP	107	3	173	113	3	179	0.06	0.17
	TClPP	107	5	-40	113	7	-34	0.37	1.12
	TDClPP	107	3	-90	113	3	-84	0.19	0.56
	TPhP	107	5	41	113	7	47	0.06	0.17
PFAS	PFPeA	71	33	174	77	32	176	2.5	7.6
	PFBS	48	31	-43	54	30	-41	0.4	1.1
	PFHpA	60	26	36	66	25	38	0.5	1.5
	PFHpS	28	16	-54	34	15	-52	0.2	0.6
	ipPFNA	46	33	-39	52	32	-37	1.3	3.9
	FOUEA	77	3	-41	83	4	-39	1.0	3.0
	ipPFN	<LOQ	-	-88	78	11	-56	4.0	12.0
	PFDS	99	4	-30	105	5	-48	1.4	4.1
	PFTrDA	102	3	-35	108	4	-43	0.0	0.1
	PFTeDA	<LOQ	-	-85	89	-	-83	4.5	13.5
	PFHxDA	<LOQ	-	-90	72	-	-88	4.3	12.9
	PFODA	106	4	-75	112	5	-73	1.7	5.0
	PFBA	71	31	133	77	30	135	1.3	3.8
	PFHxA	63	16	59	69	15	61	1.6	4.8
	PFHxS	61	18	-6	67	17	-4	0.0	0.0
	PFOA	90	11	72	96	10	74	0.1	0.2
	PFOS	38	12	-74	44	11	-72	0.8	2.5
	PFNA	54	17	-46	60	16	-44	1.5	4.5
	PFDA	100	2	-11	106	1	-9	0.1	0.2
	PFUnDA	63	3	-58	69	2	-86	0.1	0.3
	PFDoDA	<LOQ	-	-89	75	-	-87	4.0	12.0
PESTICIDES	Acetamiprid	41	21	-13	57	28	-7	2.4	7.2
	Alachlor	46	18	121	62	8	127	2.6	7.8
	Atrazine	48	32	-44	54	39	-38	2.9	8.7
	Atrazine-Deethyl	58	17.4	-17	64	7.4	-11	3.2	9.5
	Atrazine-Deisoproprył	57	26	-22	53	33	-16	1.9	5.8
	Azinphos-ethyl	107	25	-73	113	15	-67	0.4	1.2
	Azinphos-Methyl	<LOQ	-	-	95	-	-69	4.0	12.0
	Buprofezine	<LOQ	-	-	74	-	-83	5.2	15.6
	Carbendazim	48	12	8	54	19	14	0.8	2.3
	Carbofuran	<LOQ	-	-	72	-	-54	4.0	12.0
	Carbofuran 3-OH	59	25	124	65	32	130	1.5	4.6
	Chlorfenvinphos	147	18	-57	103	8	-51	1.1	3.3
	Chlorpyriphos	75	19	41	81	26	47	2.2	6.5
	Chlothianidin	69	12	70	75	2	76	2.4	7.1
	Coumaphos	64	4	-83	70	11	-77	1.4	4.2
	Diazinon	80	18	-20	86	8	-44	1.1	3.4
	Dichlofenthion	<LOQ	-	-	91	12	-71	5.0	15.0
	Dimethoate	<LOQ	-	-	82	10	-72	4.1	12.3
	Diuron	<LOQ	-	-	74	10	-69	4.8	14.4
	DMA	125	20	-87	102	20	-81	2.9	8.6
	DMF	117	2	-39	103	9	-33	2.4	7.3
	DMPF	112	21	4	107	21	10	2.5	7.6
	Ethion	102	18	-79	102	15	-73	0.3	0.8
	Etofenprox	<LOQ	-	-	78	20	-71	3.7	11.1
	Fenthion	52	15	-46	58	12	-40	0.7	2.1
	Fenthion sulfone	75	22	22	81	12	28	0.5	1.4
	Fenthion sulfoxide	46	19	16	52	25	22	1.1	3.2
	Fipronil	107	7	-85	103	3	-79	0.0	0.1
	Hexythiazox	124	16	-90	100	13	-84	0.4	1.2
	Imazalil	106	5	-90	92	5	-84	0.5	1.4
	Imidacloprid	80	7	93	86	14	99	1.1	3.4
	Isoproturon	83	14	64	89	14	70	0.6	1.8
	Isoproturon	<LOQ	-	-	84	16	-71	3.7	11.1
	Methiocarb	85	7	61	91	3	67	0.7	2.1
	Methoalachlor	31	25	-33	37	12	-27	1.9	5.8
	Molinate	68	9	141	74	1	147	0.9	2.7
	Omethoate	69	4	-28	75	11	-52	2.4	7.2
	Parathion-ethyl	<LOQ	-	-	91	25	-59	5.0	15.0
	Prochloraz	115	21	-90	121	18	-84	1.5	4.4
	Propanil	<LOQ	-	-	72	24	-76	4.0	12.0
	Propazine	78	17	21	84	24	27	0.6	1.9
	Pyriproxyfen	<LOQ	-	-	70	12	-86	3.5	10.5
	Simazine	55	4	-39	61	11	-33	1.1	3.3
	Spinosad A	31	10	-46	67	13	-40	1.9	5.8
	Spinosad C	19	19	-67	55	16	-61	3.1	9.3
	Spinosad D	107	6	-90	113	4	-84	0.6	1.7
	Tebuconazole	70	7	52	76	14	58	0.9	2.6
	Terbumeton	70	21	37	76	18	43	0.1	0.3
	Terbumeton deethyl	70	1	153	76	6	159	0.1	0.3
	Terbuthylazine	80	21	-1	86	11	5	0.1	0.2
	Terbuthylazine-2OH	111	3	-29	117	4	-23	0.5	1.6
	Terbuthylazine-deethyl	77	11	127	83	1	133	0.8	2.3
	Terbutryn	77	23	-13	83	20	-7	0.8	2.3
	Thiabendazole	78	6	132	84	4	138	0.8	2.3
	Thiametosan	79	15	66	85	22	72	1.5	4.6
PhACs	5-methyl-1H-Benzotriazole	88	6	-39	92	5	-36	0.36	1.21
	Acetaminophen	87	5	-22	96	1	-30	0.42	1.42
	Acridone	93	2.8	-15	100	4.2	-18	0.25	0.83
	Alprazolam	112	8	-12	96	15	-3	0.42	1.39

	CECs	*Procambarus clarkii*								*Anodonta cygnea*							
		10 ng g^{-1}			100 ng g^{-1}			LOD (ng g^{-1})	LOQ (ng g^{-1})	10 ng g^{-1}			100 ng g^{-1}			LOD (ng g^{-1})	LOQ (ng g^{-1})
		Recovery (%)	RSD (%)	Matrix Effect (%)	Recovery (%)	RSD (%)	Matrix Effect (%)			Recovery (%)	RSD (%)	Matrix Effect (%)	Recovery (%)	RSD (%)	Matrix Effect (%)		
PhACs	Amantadine	89	12	-66	52	1	-52	0.57	1.91	80	3	60	116	23	22	0.20	0.68
	Atenolol	87	5	-64	77	0	-70	0.16	0.53	93	4	-10	113	4	-30	0.05	0.15
	Atorvastatin	<20	-	-100	<20	-	-100	0.48	1.60	<20	-	-100	<20	-	-67	-	-
	Benzotriazole	86	1	-36	63	1	-53	1.04	3.46	90	6	1	114	6	-22	0.15	0.49
	Benzoylecgonine	102	1	-46	78	2	-55	0.69	2.30	102	6	-20	115	2	-28	0.05	0.17
	Bezafibrate	91	2	64	97	3	27	0.14	0.48	94	4	9	116	15	-20	0.06	0.20
	Bisphenol-A	85	5	-20	85	6	-40	0.16	0.55	87	8	11	118	3	-26	0.04	0.13
	Bromazepam	92	4	300	115	2	136	0.56	1.88	113	1	157	82	12	18	0.09	0.29
	Caffeine	83	4	-40	71	2	-57	0.61	2.02	72	5	-7	109	6	-31	0.09	0.31
	Carazolol	114	0.2	-86	118	11.1	-91	0.57	1.88	83	0.7	-48	120	0.7	-62	0.29	0.96
	Carbamazepine	97	1	-33	86	1	-45	0.23	0.77	101	7	-21	110	8	-32	0.11	0.38
	CBZ-10,11-epoxide	91	1	-10	73	0	-24	0.12	0.38	97	9	-9	93	18	-29	0.13	0.45
	Chloramphenicol	94	1	-23	76	5	-19	0.88	2.94	96	9	-2	117	12	-22	0.13	0.44
	Chlorpromazine	<20	-	-77	81	38	-92	0.31	1.02	43	25	-96	110	60	-86	0.09	0.31
	Ciprofloxacin	<20	36	-7	<20	4	-76	0.48	1.60	<20	-	-93	<20	9	-73	0.01	0.05
	Citalopram	82	8	-84	87	9	-88	0.30	1.00	36	9	-17	98	5	-68	0.30	1.01
	Clarithromycin	73	10	-66	103	6	-77	0.46	1.52	28	2	-68	83	17	-45	0.07	0.23
	Clofibric acid	93	2	28	96	2	-5	0.15	0.50	61	12	7	96	9	-23	0.14	0.45
	Coca-ethylene	113	15	-62	71	2	-74	0.65	2.16	107	16	-58	111	1	-46	0.04	0.12
	Cocaine	98	34	-56	57	11	-61	0.28	0.92	93	3	-41	118	4	-39	0.06	0.19
	Codeine	<20	-	-100	67	1	-65	0.30	1.01	103	6	-23	99	13	-27	0.05	0.18
	Cotinine	97	4	-63	69	1	-70	0.24	0.81	106	1	-51	117	3	-57	0.18	0.59
	Diazepam	93	1	-49	104	7	-58	0.18	0.59	178	4	-45	174	0.4	-47	0.35	1.16
	Diclofenac	95	13	-12	56	14	-13	0.16	0.53	93	4	4	99	12	-17	0.09	0.30
	Diltiazem	67	0.8	-78	78	4.4	-85	0.28	0.92	40	0.1	-16	97	8.5	-64	0.21	0.70
	Erythromycin	90	12	-54	92	1	-57	0.23	0.78	109	14	-53	114	15	-43	0.06	0.21
	Fenofibrate	<20	-	-	55	26	264	0.50	1.68	<20	-	-	<20	-	-100	-	-
	Fipronil	98	5	-43	98	9	-39	0.44	1.48	98	7	-26	116	7	-32	0.02	0.06
	Fipronil Desulfynil	99	9	-51	76	16	-69	0.29	0.98	101	9	-40	119	12	-45	0.01	0.02
	Fipronil Sulfide	92	11	-48	56	14	-14	0.75	2.50	107	11	-49	105	27	-42	-	-
	Fipronil Sulfone	91	9	-36	59	15	-2	0.19	0.63	118	15	-48	100	27	-37	1.66	5.52
	Fluconazole	61	5	10	78	1	-52	0.07	0.23	89	8	-26	98	2	-36	0.05	0.17
	Flumequine	<20	11	-4	<20	2	-51	0.53	1.76	82	7	-97	<20	11	-66	0.03	0.09
	Fluoxetine	143	32	-90	97	7	-93	0.33	1.10	70	20	-94	117	35	-88	0.16	0.55
	Furazolidone	67	14	-45	37	1	-46	0.28	0.95	99	7	-6	117	8	-16	0.14	0.47
	Furosemide	100	10	-26	89	6	-36	0.39	1.30	93	9	-14	114	17	-26	0.11	0.38
	Hydrochlorothiazide	89	16	-36	74	1	-43	0.20	0.66	101	8	-4	106	4	-22	0.12	0.40
	Indomethacin	102	1	-13	84	1	-16	0.37	1.24	108	1	-19	115	4	-27	0.15	0.50
	Ketoprofen	98	8	33	96	2	-3	0.48	1.61	93	5	3	108	18	-23	0.06	0.19
	Lamotrigine	93	16	-79	92	1	-84	0.10	0.34	101	3	-41	118	0	-45	0.04	0.15
	Loratadine	78	8	-62	104	7	-75	0.29	0.96	<20	53	-45	63	28	-82	0.50	1.68
	Lorazepam	99	10	1	89	9	-28	0.56	1.88	101	4	-44	116	5	-43	0.43	1.44
	Losartan	90	4	19	101	5	0	0.32	1.06	96	12	-54	93	5	-53	0.17	0.56
	Mefenamic acid	81	4	-19	104	3	-36	0.20	0.67	55	8	-35	54	4	-31	0.09	0.31
	Metformin	<20	-	-100	87	14	-98	0.72	2.39	100	15	-93	113	5	-91	0.09	0.31
	Methadone	75	20	-89	74	1	-91	0.52	1.74	45	11	-89	92	15	-74	0.42	1.40
	Metoprolol	97	2	-53	84	2	-63	0.19	0.62	100	2	-19	112	2	-34	0.18	0.60
	Midazolam	54	13	-82	76	19	-87	0.24	0.80	115	1	-86	118	10	-69	0.02	0.06
	Morphine	104	14	-89	75	3	-86	0.52	1.74	110	15	-63	116	4	-59	0.05	0.17
	N-acethyl SMX	115	4	-20	80	0	-21	0.27	0.90	107	6	-25	126	1	-26	0.09	0.29
	Nalidixic acid	<20	8	-22	<20	2	-56	0.39	1.29	44	1	-96	<20	0	-55	0.02	0.08
	N-Desmethylcitalopram	98	5	-98	110	9	-93	0.40	1.32	80	8	-73	120	8	-68	0.02	0.05
	Nicotine	<20	-	-100	47	1	-34	0.04	0.14	69	1	2	88	6	205	0.03	0.10
	Norfluoxetine	<20	-	-94	91	13	-93	0.50	1.67	79	37	-94	110	56	-85	0.17	0.57
	O-desmethylvenlafaxine	114	7	-56	71	2	-65	0.25	0.82	81	4	-22	107	16	-36	0.14	0.48
	Omeprazole	116	25	-49	278	6	-77	0.15	0.49	66	19	-84	35	11	-82	0.10	0.32
	Oseltamivir	102	6	-64	100	5	-67	0.39	1.31	91	2	-19	118	4	-40	0.10	0.32
	Oseltamivir-CBX	69	12	-17	69	2	-32	0.07	0.23	46	2	29	51	13	-27	0.07	0.22
	Oxazepam	96	12	32	79	1	-6	0.20	0.65	98	6	-43	118	8	-40	0.07	0.22
	Oxytetracycline	<20	-	17	<20	-	192	0.31	1.05	<20	-	-100	<20	-	83	-	-
	Paroxetine	<20	-	-92	94	6	-92	0.35	1.16	96	17	-90	83	37	-86	0.02	0.07
	Pentobarbital	91	3	-14	83	3	-13	0.92	3.07	97	7	6	113	6	-10	0.08	0.25
	Propyphenazone	100	2	-28	97	2	-48	0.04	0.12	97	8	-10	105	7	-23	0.12	0.39
	Quetiapine	71	18.1	-83	112	9.4	-87	0.27	0.90	101	0.1	-81	111	0.5	-71	0.01	0.03
	Salbutamol	97	6	-59	63	2	-61	0.19	0.64	76	2	-13	105	10	-25	0.14	0.47
	Salicylic acid	<LOQ	-	31	82	0.3	-33	6.66	22.19	20	8	18	<20	20	-18	0.16	0.52
	Sertraline	<20	-	-100	39	20	-84	0.15	0.50	<20	-	-100	44	48	-76	0.01	0.04
	Sitagliptin	99	4	-53	97	5	-64	0.34	1.13	94	5	-28	113	5	-38	0.10	0.33
	Sotalol	112	6	-67	77	1	-73	0.09	0.31	87	4	-14	114	5	-32	0.09	0.30
	Sulfadimethoxine	90	1	15	85	0	-9	0.15	0.50	99	7	-19	109	8	-35	0.23	0.76
	Sulfamethazine	98	2	-28	75	0	-33	0.39	1.30	99	20	-14	99	18	-32	0.05	0.18
	Sulfamethoxazole	98	2	-17	74	3	-36	0.30	1.00	103	13	-9	117	14	-26	0.07	0.25
	Sulfapyridine	103	5	-48	74	4	-47	0.28	0.94	72	11	-15	93	26	-29	0.11	0.36
	Temazepam	105	9	39	101	2	-7	0.19	0.65	100	3	-22	117	1	-18	0.04	0.14
	Tramadol	97	3	-48	85	1	-61	0.48	1.62	102	3	-12	86	2	-7	0.07	0.22
	Triclocarban	59	5	130	58	17	-84	0.67	2.24	<20	-	-100	34	54	99	0.81	2.69
	Trimethoprim	112	4	-69	86	2	-77	0.43	1.43	100	6	-28	114	31	-39	0.04	0.14
	Valsartan	101	14	114	87	5	70	0.18	0.58	93	19	-26	91	9	-43	0.06	0.21
	Valsartan acid	35	21	-43	67	2	-48	0.16	0.53	<20	-	-22	105	11	-38	0.16	0.52
	Venlafaxine	98	24	-63	81	9	-76	0.20	0.65	101	15	-48	111	3	-39	0.08	0.25
	Verapamil	95	25	-89	111	13	-91	0.32	1.07	44	2	-85	117	20	-72	0.18	0.62
	Warfarin	91	4	121	84	2	102	1.52	5.08	90	6	13	115	8	-7	0.09	0.30
	Zolpidem	117	8	-49	103	6	-60	0.43	1.44	117	15	-57	111	4	-45	0.03	0.09

	CECs	*Leopomis gibbosus*								*Corbicula fluminea*							
		10 ng g^{-1}			100 ng g^{-1}			LOD (ng g^{-1})	LOQ (ng g^{-1})	10 ng g^{-1}			100 ng g^{-1}			LOD (ng g^{-1})	LOQ (ng g^{-1})
		Recovery (%)	RSD (%)	Matrix Effect (%)	Recovery (%)	RSD (%)	Matrix Effect (%)			Recovery (%)	RSD (%)	Matrix Effect (%)	Recovery (%)	RSD (%)	Matrix Effect (%)		
PhACs	Amantadine	108	17	77	109	8	144	0.21	0.69	120	8	96	117	4	265	0.28	0.95
	Atenolol	97	27	-5	99	7	-30	0.09	0.29	100	5	3	100	4	-23	0.08	0.28
	Atorvastatin	<20	-	-100	<20	-	-77	-	-	<20	-	-	<20	-	-	-	-
	Benzotriazole	98	14	-33	100	9	-40	0.60	2.01	90	7	-32	101	3	-32	0.18	0.60
	Benzoylecgonine	43	7	21	100	9	-7	0.34	1.13	78	3	14	117	2	2	0.33	1.10
	Bezafibrate	99	21	14	98	6	-13	0.22	0.74	99	3	11	102	3	8	0.15	0.51
	Bisphenol-A	110	12	-42	97	17	-53	0.29	0.96	106	5	-31	99	9	-39	0.17	0.56
	Bromazepam	71	1	47	97	7	-2	0.44	1.48	100	11	-7	93	9	0	0.80	2.66
	Caffeine	86	16	44	97	7	15	0.41	1.37	92	7	60	100	4	32	0.30	1.01
	Carazolol	77	17.8	44	81	17.9	-4	0.10	0.34	87	10.0	92	87	18.1	48	0.29	0.97
	Carbamazepine	97	18	-1	101	7	-31	0.05	0.18	88	6	4	102	5	-11	0.30	0.99
	CBZ-10,11-epoxide	82	2	52	104	7	8	0.29	0.97	90	5	28	109	4	1	0.08	0.28
	Chloramphenicol	110	20	-1	118	10	-12	0.54	1.80	95	5	1	118	3	-15	0.25	0.83
	Chlorpromazine	86	7	300	81	5	213	0.16	0.54	100	16	300	97	20	300	0.10	0.33
	Ciprofloxacin	<20	-	-92	<20	20	-13	0.48	1.60	<20	-	31	<20	-	25	0.14	0.48
	Citalopram	119	19	-45	98	2	-40	0.49	1.65	89	10	-8	92	8	-7	0.13	0.42
	Clarithromycin	78	15	-45	80	14	-51	0.61	2.03	84	10	-11	80	18	-9	0.22	0.74
	Clofibric acid	89	19	-8	109	12	-10	0.28	0.92	94	4	-7	108	5	-5	0.19	0.64
	Coca-ethylene	117	5	-34	111	12	-31	0.40	1.32	97	10	-9	94	17	-9	0.33	1.09
	Cocaine	100	11	-16	98	6	-32	0.23	0.76	96	7	7	99	7	0	0.24	0.78
	Codeine	92	18	-23	100	8	-42	0.13	0.42	96	6	-41	98	4	-46	0.15	0.51
	Cotinine	78	12	-17	81	7	-34	0.25	0.84	91	5	-43	98	6	-44	0.16	0.54
	Diazepam	107	20	-2	110	11	-49	0.23	0.75	100	9	-13	87	20	-26	0.08	0.28
	Diclofenac	<20	-	-49	93	16	-45	0.13	0.43	83	16	-45	98	17	-35	0.16	0.54
	Diltiazem	91	12.8	48	89	3.6	-39	0.28	0.94	76	12.1	151	87	6.4	-9	0.06	0.19
	Erythromycin	97	28	-57	98	5	-66	0.05	0.16	95	5	-43	104	4	-34	0.21	0.70
	Fenofibrate	<20	-	80	99	3	5	0.49	1.63	<20	-	-100	103	1	119	0.32	1.07
	Fipronil	74	4	-29	99	1	61	0.28	0.93	78	5	1	100	9	150	0.13	0.42
	Fipronil Desulfynil	58	12	-12	119	7	-56	0.48	1.60	96	7	-13	105	3	-27	0.27	0.89
	Fipronil Sulfide	49	17	-31	117	0	-51	0.13	0.44	60	20	138	112	6	12	0.48	1.59
	Fipronil Sulfone	49	14	-31	107	1	-24	0.44	1.47	66	2	156	128	22	53	0.19	0.64
	Fluconazole	99	23	-7	98	7	-28	0.14	0.46	93	4	-8	99	6	-16	0.18	0.61
	Flumequine	54	11	-76	29	21	-25	0.54	1.80	28	7	-4	42	13	3	0.46	1.53
	Fluoxetine	101	9	300	102	21	175	0.12	0.40	95	7	300	102	7	293	0.19	0.65
	Furazolidone	62	3	300	73	5	-46	0.40	1.33	46	18	300	52	9	-17	0.07	0.23
	Furosemide	83	35	-39	89	19	-43	0.19	0.63	<20	-	-100	<20	-	-87	0.31	1.02
	Hydrochlorothiazide	65	16	-14	94	13	-24	0.24	0.81	80	8	-16	96	2	-21	0.06	0.20
	Indomethacin	<20	-	62	88	1	-62	0.28	0.93	<20	-	-44	109	12	-65	0.24	0.82
	Ketoprofen	93	2	0	95	6	-30	0.25	0.84	104	7	-4	103	2	-9	0.26	0.86
	Lamotrigine	116	2	-64	107	13	-70	0.43	1.44	96	10	-78	116	17	-74	0.23	0.78
	Loratadine	68	3	77	63	14	-7	0.51	1.70	62	9	70	62	6	27	0.95	3.15
	Lorazepam	71	9	39	101	16	-8	0.21	0.71	85	7	18	102	15	4	0.41	1.37
	Losartan	98	18	-78	74	12	-68	0.30	1.01	94	13	-94	89	2	-84	1.37	4.56
	Mefenamic acid	106	23	-65	115	18	-78	0.25	0.84	57	7	-54	98	15	-66	0.50	1.66
	Metformin	<20	-	-100	39	14	-98	0.25	0.84	<20	11	-94	92	3	-98	0.31	1.02
	Methadone	92	15	7	97	2	25	0.59	1.96	75	13	92	83	9	77	0.09	0.29
	Metoprolol	86	10	-2	98	8	-21	0.11	0.37	91	6	2	97	5	-2	0.14	0.46
	Midazolam	97	20	-61	86	3	-59	0.08	0.28	110	13	-71	104	19	-63	0.44	1.46
	Morphine	<20	-	-54	72	58	-66	0.26	0.86	<20	-	-83	80	2	-63	0.10	0.33
	N-acethyl SMX	92	20	10	105	11	-4	0.56	1.85	115	8	-2	118	7	-3	0.71	2.36
	Nalidixic acid	35	1	-79	<20	13	-36	0.76	2.53	<20	6	-22	32	6	-16	0.09	0.29
	N-Desmethylcitalopram	101	26	-48	88	2	-38	0.68	2.27	118	0	-28	92	0	-7	0.47	1.58
	Nicotine	<20	-	75	<20	6	300	0.52	1.74	47	9	66	110	5	300	0.21	0.70
	Norfluoxetine	<20	-	221	99	13	97	0.13	0.43	85	11	300	98	12	286	0.13	0.45
	O-desmethylvenlafaxine	65	13	-24	91	6	-39	0.30	1.01	91	5	-16	114	4	-22	0.25	0.85
	Omeprazole	<20	-	-100	<20	-	-99	-	-	<20	-	-100	<20	-	-100	-	-
	Oseltamivir	73	0.2	50	103	12	1	0.18	0.60	101	6	32	97	8	15	0.13	0.44
	Oseltamivir-CBX	<20	-	12	51	14	-15	0.62	2.08	59	8	-4	76	2	-13	0.07	0.22
	Oxazepam	107	14	39	103	11	-12	0.51	1.71	91	6	11	100	14	-2	0.22	0.72
	Oxytetracycline	<20	-	-100	<20	-	-25	0.97	3.25	<20	-	-10	<20	-	5	0.54	1.80
	Paroxetine	103	12	267	109	6	82	0.12	0.38	111	8	231	95	15	160	0.16	0.53
	Pentobarbital	90	7	-25	100	7	-34	0.18	0.59	98	5	-13	101	3	-18	0.19	0.64
	Propyphenazone	93	15	18	97	6	-28	0.18	0.58	86	3	30	98	4	9	0.06	0.21
	Quetiapine	94	23.3	-48	99	8.7	-22	0.05	0.16	96	11.3	-19	100	2.8	11	0.05	0.16
	Salbutamol	64	33	-22	88	9	-35	0.24	0.81	76	3	-26	92	4	-29	0.44	1.45
	Salicylic acid	90	18	33	75	3	-13	0.47	1.56	90	16	89	79	8	8	0.34	1.12
	Sertraline	74	7	300	80	8	299	0.04	0.12	95	10	300	100	9	300	0.07	0.24
	Sitagliptin	73	21	-18	97	11	-30	0.07	0.23	92	7	-7	101	5	-8	0.10	0.32
	Sotalol	103	14	-16	96	7	-31	0.17	0.56	92	8	-12	99	5	-25	0.13	0.43
	Sulfadimethoxine	89	20	24	72	11	-4	0.13	0.44	79	10	-23	91	10	-26	0.62	2.07
	Sulfamethazine	51	9	34	101	14	-3	0.14	0.47	82	13	-1	99	14	-9	0.18	0.59
	Sulfamethoxazole	93	19	9	99	10	-15	0.09	0.32	86	7	12	107	10	-6	0.12	0.39
	Sulfapyridine	102	10	12	111	19	10	0.35	1.15	118	11	13	114	10	6	0.27	0.91
	Temazepam	89	21	60	97	10	2	0.13	0.44	95	5	60	104	8	33	0.09	0.30
	Tramadol	77	6	77	99	10	80	0.10	0.33	87	10	87	99	5	139	0.16	0.53
	Triclocarban	<20	-	201	104	7	300	1.47	4.91	53	5	300	118	7	300	0.43	1.43
	Trimethoprim	86	17	-34	97	10	-50	0.21	0.69	93	6	-34	96	4	-38	0.18	0.58
	Valsartan	67	27	-24	95	8	-35	0.09	0.31	94	7	-63	99	6	-60	0.26	0.85
	Valsartan acid	<20	-	-74	99	3	-69	0.43	1.42	<20	-	-76	109	14	-70	0.32	1.08
	Venlafaxine	95	18	6	84	12	0.5	0.09	0.31	92	4	35	100	13	32	0.10	0.34
	Verapamil	114	21	-44	90	20	-39	0.65	2.16	106	11	18	105	7	12	0.15	0.50
	Warfarin	99	16	209	100	12	109	0.28	0.93	90	5	160	101	15	64	0.42	1.41
	Zolpidem	101	14	-48	79	18	-41	0.33	1.10	90	15	-42	100	1	-31	0.71	2.37

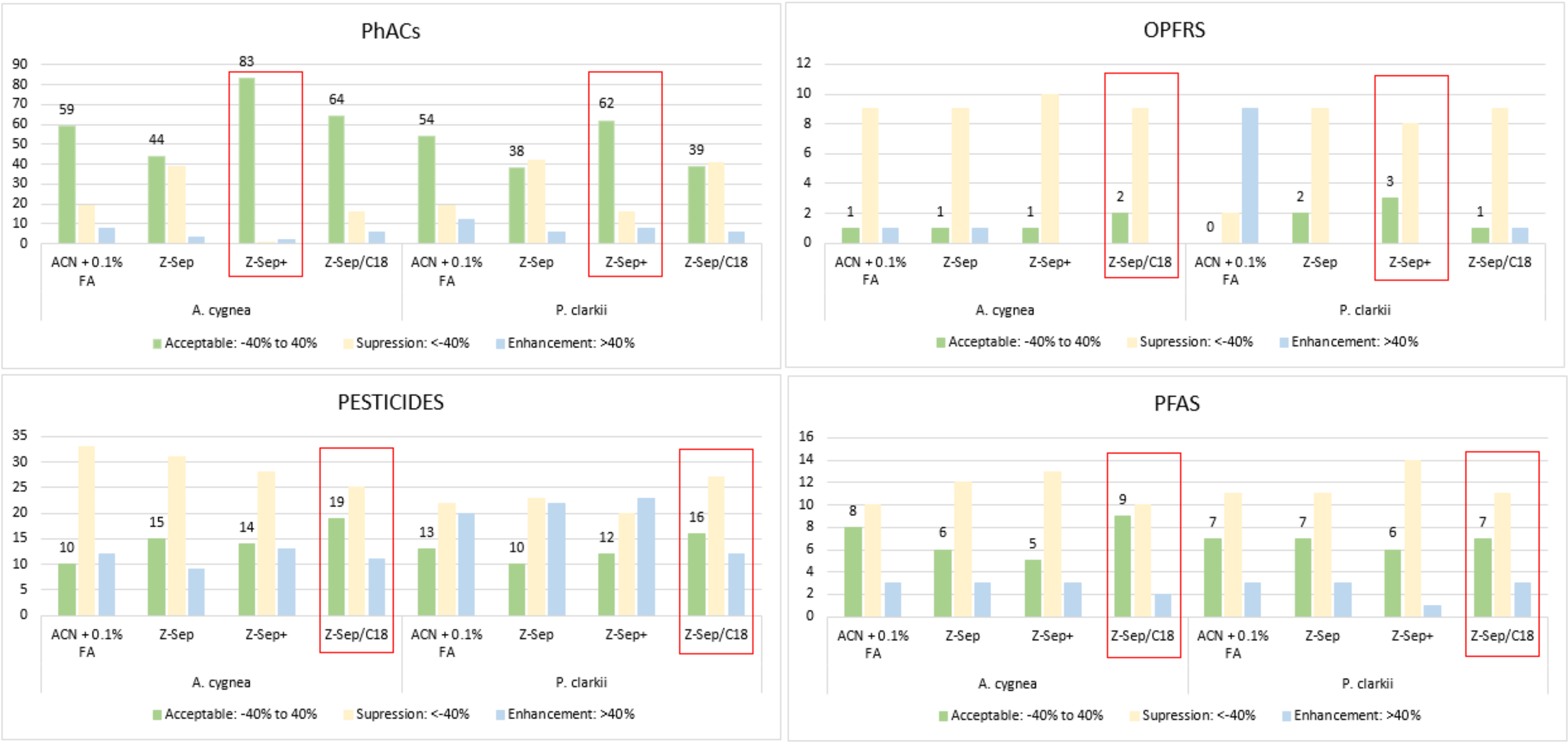

Fig. S1. Matrix effect before and after the clean-up step
